# Intragenic MicroRNAs Autoregulate Their Host Genes in Both Direct and Indirect Ways—A Cross-Species Analysis

**DOI:** 10.3390/cells9010232

**Published:** 2020-01-17

**Authors:** Maximilian Zeidler, Alexander Hüttenhofer, Michaela Kress, Kai K. Kummer

**Affiliations:** 1 Institute of Physiology, Medical University of Innsbruck, 6020 Innsbruck, Austria; 2 Institute of Genomics and RNomics, Medical University of Innsbruck, 6020 Innsbruck, Austria

**Keywords:** microRNA, intragenic, extragenic, autoregulation, species, intronic, exonic

## Abstract

MicroRNAs (miRNAs) function as master switches for post-transcriptional gene expression. Their genes are either located in the extragenic space or within host genes, but these intragenic miRNA::host gene interactions are largely enigmatic. The aim of this study was to investigate the location and co-regulation of all to date available miRNA sequences and their host genes in an unbiased computational approach. The majority of miRNAs were located within intronic regions of protein-coding and non-coding genes. These intragenic miRNAs exhibited both increased target probability as well as higher target prediction scores as compared to a model of randomly permutated genes. This was associated with a higher number of miRNA recognition elements for the hosted miRNAs within their host genes. In addition, strong indirect autoregulation of host genes through modulation of functionally connected gene clusters by intragenic miRNAs was demonstrated. In addition to direct miRNA-to-host gene targeting, intragenic miRNAs also appeared to interact with functionally related genes, thus affecting their host gene function through an indirect autoregulatory mechanism. This strongly argues for the biological relevance of autoregulation not only for the host genes themselves but, more importantly, for the entire gene cluster interacting with the host gene.

## 1. Introduction

All higher eukaryotic genomes harbor protein-coding genes that are required for cellular function, but these resemble only about 1% of the entire genome, whereas 99% do not encode for proteins [1]. Within those non-coding regions, non-coding RNAs (ncRNAs) are embedded, which exert distinct biological roles. MicroRNAs (miRNAs) are currently the most intensely investigated members of the ncRNA family and are generally accepted as post-transcriptional regulators of gene expression. As the number of newly discovered miRNAs is constantly increasing, and novel ways of miRNA processing are emerging, our understanding of the importance and the regulation of miRNA expression is becoming increasingly important [2].

miRNA genes are present on all chromosomes, with the exception of the human Y chromosome. In previous studies, it has been reported that more than 50% of miRNA-coding genes are located within the extragenic space, thereby possessing their own regulatory elements. The mechanisms regulating the expression of these extragenic miRNAs remain largely elusive. The remaining miRNA genes reside within host genes, and these intragenic miRNAs and their host mRNAs may be processed from the same RNA substrate [3,4]. Individual intragenic miRNAs are often co-expressed with their host gene [5,6,7,8,9,10,11]. Thus, mammalian intragenic miRNAs and their host genes are suggested to be derived from the same primary transcripts [2,5,12,13,14,15,16,17,18,19].

The relation of miRNAs with their host genes has been mainly demonstrated for cancer [20,21,22]. Especially those intragenic miRNAs exhibiting a high degree of conservation between species, appear to be coordinately regulated and expressed with their host genes, either with synergistic or antagonistic correlation patterns [23]. From the same study, 27 miRNA/host gene pairs emerged with cross-species conserved co-location, co-expression, and potential co-regulation [23]. In addition, human miRNA evolution is tightly influenced by the genomic context, especially by host genes. miRNAs within ancient host genes are significantly more broadly expressed than those within young genes, and young miRNAs within old genes are more broadly expressed than their extragenic counterparts. This suggests that young miRNAs have an initial advantage by residing in old genes and benefit from their hosts’ expression control and from the exposure to diverse cellular contexts and target genes [24,25,26]. Thus, host genes may provide expression constraints to intragenic miRNAs, and the genomic context and host genes are considered as driving factors for the expression and evolution of human miRNAs. However, miRNA coregulation with their host genes may be of wider relevance for multiple cellular processes, and molecular pathway analysis platforms have been established to tackle these interactions [3,4]. Since then, in addition to the 2654 known human miRNA sequences [27], 3707 new miRNAs have been identified [28], resulting in a total of 6361 miRNA sequences for which function and regulation of generation are only partially known.

Functionally, miRNAs regulate the expression of protein-coding or non-coding transcripts in a sequence-specific manner through the base pairing of the miRNA seed sequence or the 3′UTR of the target mRNA, by acting at interactor elements on promoter sequences or even binding to RNA segments within a given gene [29,30]. As a result of these interactions at miRNA recognition elements (MREs) and dependent on the degree of complementarity, mRNA stability and/or translation is impaired, resulting in a reduction in mRNA or protein expression levels [31].

These processes may not only occur at miRNA target genes, but intragenic miRNAs may interact with their own host genes through the same mechanisms and may not only involve miRNAs targeting their host genes, but also an interaction with functionally related genes and an indirect autoregulatory mechanism. Therefore, the aim of this study was to perform an in-depth analysis of the location and co-regulation of all to date available miRNA sequences and their host genes in an unbiased computational approach for four well-annotated species (human, mouse, zebrafish, and drosophila). Our data support a regulatory role of miRNAs for their host genes through direct and indirect mechanisms, arguing for the biological relevance of miRNA-to-host gene autoregulation.

## 2. Materials and Methods

### 2.1. Annotation and Mapping of MicroRNAs and Genes

**Figure 1:**
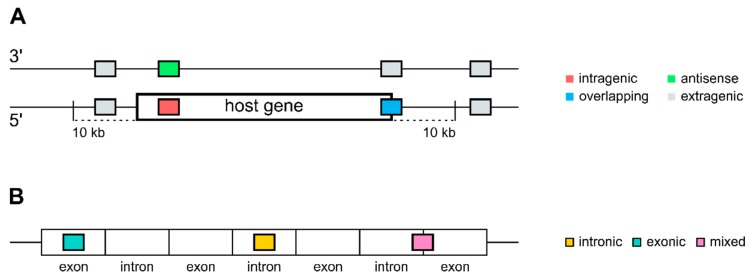
Schematic sketch of defined genomic locations of all miRNAs annotated in miRBase. (**A**) Intragenic (red), antisense (green), overlapping (blue), and extragenic miRNAs (grey) were defined based on the location of the microRNAs (miRNAs) with respect to all currently annotated genes. (**B**) Intragenic miRNAs on transcript level were further classified as intronic (yellow), exonic (turquoise), and mixed intronic/exonic miRNAs (pink).

miRNA genomic locations for the different species investigated were retrieved from miRBase (http://www.mirbase.org) release 22 [32,33,34,35], which is based on genome assemblies GRCh38 (human—*homo sapiens*—HSA), GRCm38 (mouse—*mus musculus*—MMU), GRCz11 (zebrafish—*danio rerio*—DRE), and BDGP6 (fruit fly—*drosophila melanogaster*—DME; Supplementary Figure S1). miRNAs were subsequently mapped onto gene annotations retrieved from the Ensembl genome database project (http://www.ensembl.org) Release 93 [36], which is based on genome assemblies GRCh38.p12 (HSA), GRCm38.p6 (MMU), BDGP6 (DME), and GRCz11 (DRE) by applying different custom written R (https://www.r-project.org) scripts: miRNAs that are located within the start- and end-coordinates of genes on the same strand were defined as intragenic; miRNAs that are located within genes but on the opposite strand were defined as antisense; miRNAs that are overlapping with genes but are not completely within a gene were defined as overlapping; and miRNAs that were neither intragenic, antisense nor overlapping were defined as extragenic (Figure 1). For the group of extragenic miRNAs, we, in addition, defined a group of miRNAs that were closely located to genes within 10 kilobases up- or downstream as near-gene miRNAs. Intragenic miRNAs were further classified into intronic, exonic, and mixed intronic–exonic (overlapping between introns and exons, or varying between different transcripts of a gene), depending on their location within genes. In addition to the four species investigated using the miRBase miRNA annotations, miRNA genomic locations from all 189 available species on Ensembl were retrieved and subsequently mapped onto the corresponding gene annotations.

### 2.2. Protein-Protein Interactions, Functional Enrichment and Pathway Analyses of Host Genes

Protein-protein interactions (PPI) for intragenic miRNA host genes were investigated using the STRING database (http://www.string-db.org) v11 [37], which includes direct and indirect protein associations collected from different databases. Interaction networks were prepared using medium confidence scores (0.40) and clustered using the Markov cluster algorithm (MCL; inflation parameter: 3). Disconnected nodes were hidden from the network for easier comprehension.

Functional enrichment and pathway analyses were performed using the g:GOSt gene group functional profiling tool from the g:Profiler web server (http://biit.cs.ut.ee/gprofiler), which is based on Ensembl release 96 and the genome assembly GRCh38 [38].

Classification systems tested were Gene Ontology (biological process, cellular component, and molecular function) and Kyoto Encyclopedia of Genes and Genomes (KEGG) functional annotation spaces, employing the ontology-focused multiple testing correction method g:SCS [39] with a significance threshold of *p* < 0.05. g:Profiler results were hierarchically clustered and filtered for the Best Per Parent pathway. Pathways for biological process (GO:BP), molecular function (GO:MF), and cellular components (GO:CC) were extracted and graphically illustrated.

### 2.3. Intragenic miRNA Target Prediction

To determine possible target relationships between intragenic, antisense, and overlapping miRNAs and host genes, the Diana Tools REST API (http://diana.imis.athena-innovation.gr/DianaTools) was accessed by applying custom-written Python (https://www.python.org) scripts. For miRNA target predictions, the microT-CDS v5 algorithm [40] was applied without setting a detection threshold, and miTG target prediction score and number of predicted binding sites were extracted for miRNA::host gene interactions. Genes that were not detected by microT-CDS were excluded from the analysis.

As a significant number of host genes were predicted to be targeted by both mature variants (i.e., -5p and -3p) of a given miRNA, a modified version of the DIANA miTG target prediction score (mDPS) was calculated. This mDPS includes the separate prediction scores for both mature variants, where the prediction score of the miRNA variant of higher target probability (i.e., -Xp) is combined with the squared product of the prediction score of higher probability with the prediction score of lower probability (i.e., -Yp):

mDPS = prediction-score(-Xp) + (prediction-score(-Xp) × prediction-score(-Yp))^2^ (1)

This allowed for a conservative incorporation of both separate prediction scores to reflect the increased probability of regulation of the host genes.

It was shown that enrichment analysis produced different results when applied to predicted targets or validated targets, and computational predictions may not overestimate the number and extent of meaningful miRNA-regulated processes [41,42]. To further increase the predictive validity of miRNA::host gene interactions, information about experimentally validated miRNA::gene targets was retrieved from the databases DIANA TarBase v8 [43], miRTarBase v7 [44] and starBase v2 [45]. Since the DIANA TarBase v8 also contains negative validations, only validations marked as positive were used for further analysis.

### 2.4. Iterative Randomized Model (IRM)

The specificity of miRNA::host gene pairs was assessed applying an iterative randomized model (IRM). Two separate IRM approaches were employed: IRM1 tested the list of intragenic, antisense, overlapping or near-gene miRNAs against a randomly sampled list of protein-coding genes from all protein-coding genes to elucidate specificity of miRNAs; IRM2 tested a randomly sampled list of non-intragenic miRNAs against the list of host genes for intragenic miRNAs to test for specificity of host gene targets. IRM sampling was performed over 100 iterations, and for each run, the mean mDPS and the mean target frequency were determined. Subsequently, Grubb’s outlier test was applied to identify significant divergence from the randomized sampled distribution.

### 2.5. Essentiality of Host Genes

Host genes from intragenic miRNAs per species were evaluated for their critical implications in cell survival and viability. The Online Gene Essentiality Database (OGEE, http://ogee.medgenius.info) was used to assign the essentiality status of each miRNA host gene for the separate species [46]. Only genes explicitly annotated as significantly essential in the OGEE database were considered, and the remaining genes were annotated as non-essential genes.

### 2.6. Community Detection

Indirect autoregulation of host genes by intragenic miRNAs was assessed by performing community detection for all genes currently enlisted in the StringDB database and determining the community affiliation of the host genes for both HSA and MMU. The PPI confidence score was set to the highest confidence (≥0.9).

Community detection was performed using the best-partition algorithm of the Python package “Louvain Community Detection” with the resolution parameter set to default. A community was defined as harboring at least 5 genes and 5 intragenic miRNAs. The effect of the intragenic miRNAs within a community was assessed by the number of validated targets found in the mirTarbase, Tarbase and Starbase database and normalized to the number of genes (“gene ratio”), the number of miRNAs (“miRNA ratio”) and the overall number of possible miRNA::gene interactions (“interaction ratio”) in the community.

An interaction network of the influence of intragenic miRNAs between communities was constructed applying the MultiDiGraph method from the Python package NetworkX. In- and out-degrees for each community were determined based on the interaction ratio:

Interaction ratio = (*v*/(*c_g_* × *c_m_*)) × 100 (2)

where *v* is the number of validated miRNA::gene interactions found in the community, *c_g_* the overall number of genes per community, and *c_m_* the overall number of intragenic miRNAs per community.

### 2.7. Data Analysis and Graphical Representation

For statistical analysis, GraphPad Prism v8 (Graphpad Software, San Diego, CA, USA) (Student’s *t*-test, Grubb’s outlier test, Kruskal–Wallis H-tests, Chi-squared test with Yates correction) and the Python v3.7 (Python Software Foundation, Beaverton, OR, USA) packages Matplotlib, Numpy, SciKit, Pandas, Requests, and Statsmodel were used as appropriate. The level of statistical significance was predefined at *p* < 0.05 for all statistical tests and corrected for multiple comparisons where appropriate.

For a graphical representation of the data, GraphPad Prism v8 and the Python 3.7 packages Seaborn, Matplotlib, Numpy, NetworkX, and Pandas were applied. Graphical post-processing was performed using CorelDraw X8 (Corel Corporation, Ottawa, ON, Canada).

## 3. Results

### 3.1. Distribution of Intragenic miRNAs Across Different Species

**Figure 2:**
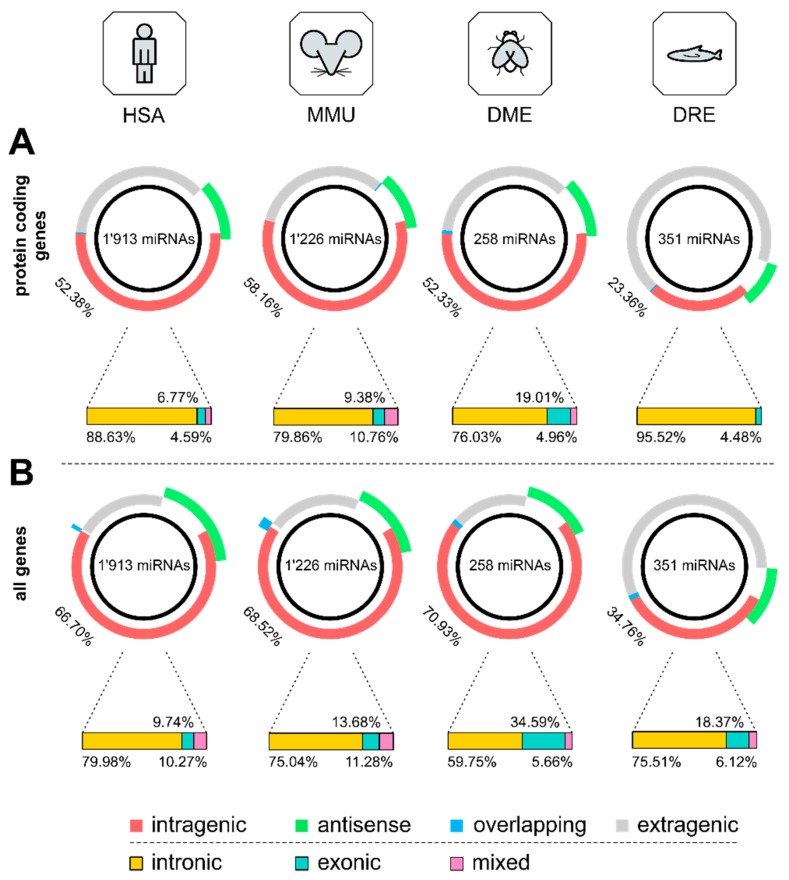
Percentage of intragenic, antisense, overlapping, and extragenic miRNAs with respect to protein-coding genes (**A**) and all-genes (**B**) in HSA, MMU, DME, and DRE are depicted in pie charts. The overall number of miRNAs annotated by miRBase is depicted for all species. The spatial distribution of miRNAs at gene transcript-level resolution identified intronic, exonic, and mixed intronic–exonic miRNAs across the species (**A**,**B**).

Analysis of genomic locations of miRNAs which were mapped onto gene annotations revealed that the majority of miRNAs was located within protein-coding genes (i.e., intragenic miRNA; HSA 52.38%, MMU 58.16%, DME 52.33%, DRE 23.36%), followed by a smaller fraction of miRNAs located on the antisense strand (i.e., antisense miRNA; HSA 12.08%, MMU 11.66, DME 12.02%, DRE 9.12%), and a small number of miRNAs at least in part overlapping with a gene (overlapping miRNA; HSA 0.31%, MMU 0.33%, DME 0.78%, DRE 0.28%; Figure 2A; Table 1). More detailed analysis of the location of intragenic miRNAs at the transcript level showed that the by far largest percentage of intragenic miRNAs were located in intronic regions (intronic miRNA; HSA 88.63%, MMU 79.86%, DME 76.03%, DRE 95.52%), whereas only a small number of miRNAs was located in exonic regions (exonic miRNA; HSA 6.77%, MMU 9.38%, DME 19.01%, DRE 4.48%) or variably located in introns or exons across different transcript variants, or even spanning multiple intronic and exonic regions (mixed miRNA; HSA 4.59%, MMU 10.76%, DME 4.96%, DRE 0.00%; Figure 2A, Table 1). This distribution of intragenic and intronic miRNAs based on protein-coding genes is slightly higher than previous reports stating that roughly half of the known miRNA genes are located within previously annotated protein-coding regions [4,47]. However, when extending mapping of miRNAs to not only protein-coding but all genes (i.e., including protein-coding genes, pseudogenes, lncRNAs, predicted genes, etc.), an even larger number of miRNAs was found to be located within annotated gene transcripts (HSA 66.70%, MMU 68.52%, DME 70.93%, DRE 34.76%), on the antisense strand (HSA 19.45%, MMU 15.58%, DME 13.95%, DRE 11.97%), or partially overlapping with a gene (HSA 0.84%, MMU 2.20%, DME 1.16%, DRE 1.42%; Figure 2B; Table 1). Analysis on transcript level showed that for all genes the proportion of miRNAs located in intronic gene regions was lower (HSA 79.98%, MMU 75.04%, DME 59.755, DRE 75.51%), whereas the number of exonic miRNAs (HSA 9.74%, MMU 13.68%, DME 34.59%, DRE 18.37%) and mixed miRNAs (HSA 10.27%, MMU 11.28%, DME 5.66%, DRE 6.12%) was higher than for only protein-coding transcripts (Figure 2B, Table 1).

**Figure 3:**
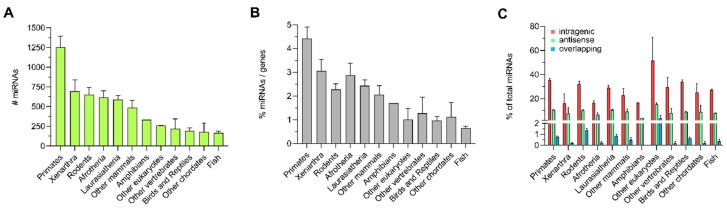
All 189 currently annotated species in the Ensembl catalog were subjected to miRNA location analysis and grouped according to the Ensembl species categories. Entire miRNA occurrence for all species categories in absolute numbers (**A**), as well as normalized to the total number of all-genes (**B**) compared between the groups. Percentage of intragenic, antisense, and overlapping miRNAs was determined for each species group and compared between the groups (**C**). Interspecies group comparisons were performed using a one-way ANOVA with a *p*-value threshold set to <0.05.

Interestingly, the distribution of miRNA locations in zebrafish significantly differed from human, mouse, and fly with only ~24% and ~35% of miRNAs located in protein-coding or all genes, respectively. To further investigate the number of intragenic miRNAs across different species and test if there were prominent species differences, we performed the same analysis with the entire Ensembl catalog of 189 available species. The number of different miRNA groups per species was determined, and the species clustered according to the respective Ensembl “order” assignment (a list of species, miRNA groups and classification can be found in Supplementary Table S1). The absolute number of miRNAs between the orders of animals was significantly different, with the order “Primates” exhibiting the highest number of miRNAs (Figure 3A; one-way ANOVA, F_(11,177)_ = 16.29, *p* < 0.0001). In addition, the average percentage of miRNAs compared to the total number of genes showed significant differences between the orders of animals, with the order “Primates” showing the highest proportion of miRNAs in the genome and the order “Fish” showing the lowest (Figure 3B; one-way ANOVA, F_(11,160)_ = 12.63, *p* < 0.0001). When focusing on the average distribution of the different types of miRNAs, in different orders of animals varying proportions of intragenic, antisense, and overlapping miRNAs were detected (Figure 3C; two-way ANOVA, species F_(11,531)_ = 6.451, *p* < 0.0001; miRNA types F_(2,531)_ = 246.3, *p* < 0.001; interaction F_(22,531)_ = 3.104, *p* < 0.0001). Interestingly, the percentage of intragenic miRNAs from the four species investigated in detail was considerably higher than the average percentage of the respective order (Figure 3C). These discrepancies between the species are likely due to the higher availability and quality of data sets derived from humans and animal models frequently used in biomedical research vs. the incompleteness of miRNA and gene annotations for other species [27,48].

### 3.2. miRNA Host Genes Show Functional Clusters and Pathway Enrichment

To test whether protein-coding genes, hosting intragenic miRNAs, were functionally related, the lists of host genes per species were subjected to protein–protein interaction (PPI) and pathway enrichment analyses. We first performed a PPI analysis using the STRING database, followed by Markov chain network clustering. This revealed a significant PPI enrichment for all four species (see Supplementary Table S2 for the network statistics per species). Cluster analysis revealed several functionally connected clusters of host genes with at least five nodes for HSA (35 clusters; Supplementary Figure S2), MMU (19 clusters, Supplementary Figure S4) and DME (2 clusters; Supplementary Figure S6), whereas no functional clusters of the respective complexity were found for DRE. The detected clusters were further analyzed for pathway enrichment, which revealed specific functions of the individual host gene clusters (see Supplementary Figure S3 for HSA, Supplementary Figure S5 for MMU, Supplementary Figure S7 for DME). Of those, 19 GO and 11 KEGG pathways were conserved in both HSA and MMU clusters, including pathways important for ubiquitination (GO:0000209: protein polyubiquitination, GO:0019005 SCF ubiquitin ligase complex, GO:0061630 ubiquitin-protein ligase activity, KEGG:04120 ubiquitin-mediated proteolysis), neuron and neurotransmitter related pathways (GO:0072583 clathrin-dependent endocytosis, KEGG:04360 axon guidance, KEGG:04721 synaptic vesicle cycle, KEGG:04724 glutamatergic synapse, KEGG:04725 cholinergic synapse) or RNA modification and translation (GO:0001732 formation of cytoplasmic translation initiation complex, GO:0016282 eukaryotic 43S preinitiation complex, GO:0033290 eukaryotic 48S preinitiation complex, GO:0003743 translation initiation factor activity, KEGG:03013 RNA transport, KEGG:03040 spliceosome).

The conservation of specific pathways is further supported by the observation that 143 of the protein-coding genes were found in both mouse and human in a direct literal comparison of gene names, indicating that a large proportion of important host genes appear to be conserved between HSA and MMU. Assignment of the homologous genes to the respective clusters revealed that HSA cluster 2 (Supplementary Figure S2) and MMU cluster 1 (Supplementary Figure S4) shared four genes (KLHL3, RNF130, HUWE1, and CCNF), whereas HSA cluster 4 and MMU cluster 6 shared three genes (COPZ1, COPZ2, and R3HDM1). Furthermore, the pathway analysis for the respective clusters showed that both HSA cluster 2 and MMU cluster 1 showed functional enrichment of ubiquitin-related pathways (e.g., GO:0000209 protein polyubiquitination, GO:0061630 ubiquitin-protein ligase activity), whereas both HSA cluster 4 and MMU cluster 6 showed functional enrichment of Golgi-related pathways (e.g., GO:0006890 retrograde vesicle-mediated transport, Golgi to endoplasmic reticulum).

**Figure 4:**
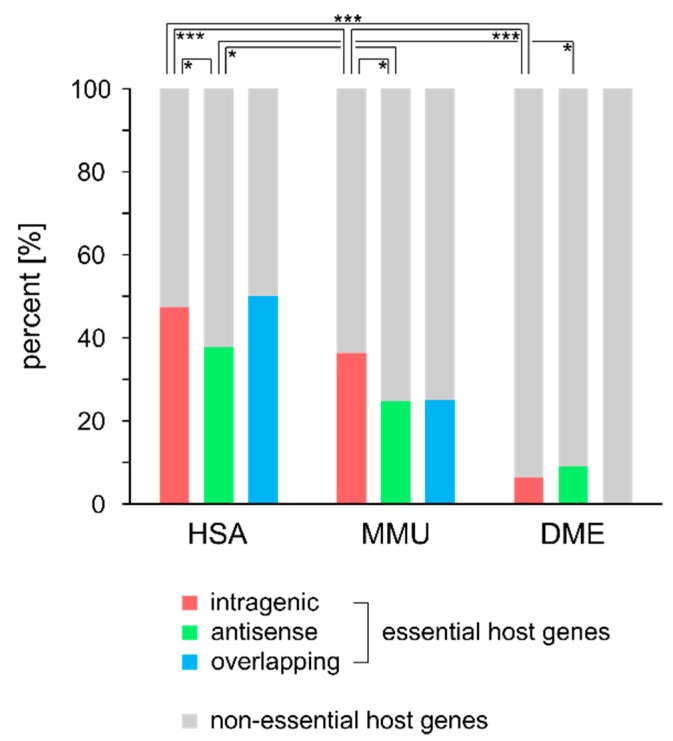
Percentage of essential intragenic, antisense, and overlapping miRNA host genes is shown for HSA, MMU, and DME. Frequency of essential intragenic, antisense, and overlapping host genes was compared intraspecies and interspecies using a Chi-square test with a *p*-value threshold set to <0.05 (*), 0.001 (***).

To further investigate the functional relevance of protein-coding genes hosting intragenic miRNAs, we classified these host genes into essential and non-essential genes based on the data available from the OGEE database (Supplementary Table S3). When comparing the proportion of essential vs. non-essential genes over the different miRNA types and species, HSA showed the highest proportion of miRNAs hosted in essential genes, whereas the majority of miRNAs in DME was hosted in non-essential genes (Figure 4; Chi-square, post-hoc tests Bonferroni corrected; HSA, χ^2^_(2)_ = 6.43, *p* = 0.040, intragenic vs. antisense, χ^2^_(1)_ = 6.38, *p* = 0.036; MMU, χ^2^_(2)_ = 6.11, *p* = 0.047, intragenic vs. antisense, χ^2^_(1)_ = 5.95, *p* = 0.044; DME, *p* = 0.80; intragenic, χ^2^_(2)_ = 84.67, *p* < 0.001, HSA vs. MMU, χ^2^_(1)_ = 18.53, *p* < 0.001, HSA vs. DME, χ^2^_(2)_ = 77.27, *p* < 0.001, MMU vs. DME, χ^2^_(1)_ = 44.20, *p* < 0.001; antisense, χ^2^_(2)_ = 11.61, *p* = 0.003, HSA vs. MMU, χ^2^_(1)_ = 5.95, *p* = 0.044, HSA vs. DME, χ^2^_(1)_ = 7.24, *p* = 0.021). To ensure that the proportion of essential genes hosting the different classes of miRNAs did not occur by chance, the absolute numbers of essential and non-essential genes were compared to the overall number of essential and non-essential genes found in the OGEE database for each species. Since the number of genes contained in the OGEE essentiality database highly varies across species (HSA: 21,566, MMU: 9042, DME: 14,218), the results obtained for HSA and DME are more likely representing the actual essentiality state for each gene, whereas the MMU essentiality state might be affected by the low coverage number. Comparing the proportion of essential genes for HSA and DME to the proportion of all currently tested genes revealed a significantly higher number of essential genes for intragenic miRNA harboring host genes (Chi-square; HSA, χ^2^_(2)_ = 79.47, *p* < 0.00091; DME χ^2^_(2)_ = 4.11, *p* = 0.043), while no differences were detected for host genes harboring antisense miRNAs (Chi-square; HSA, χ^2^_(2)_ = 1.82, *p* > 0.05; DME χ^2^_(2)_ = 1.22, *p* > 0.05; Table 2).

### 3.3. Intragenic miRNAs Target Their Host Genes

Considering the small proportion of protein-coding DNA regions in the genome, intragenic miRNA location may represent an evolutionary advantage. Thus, we hypothesized that intragenic miRNAs could function as autoregulatory switches, either on a host gene or functional level.

**Figure 5:**
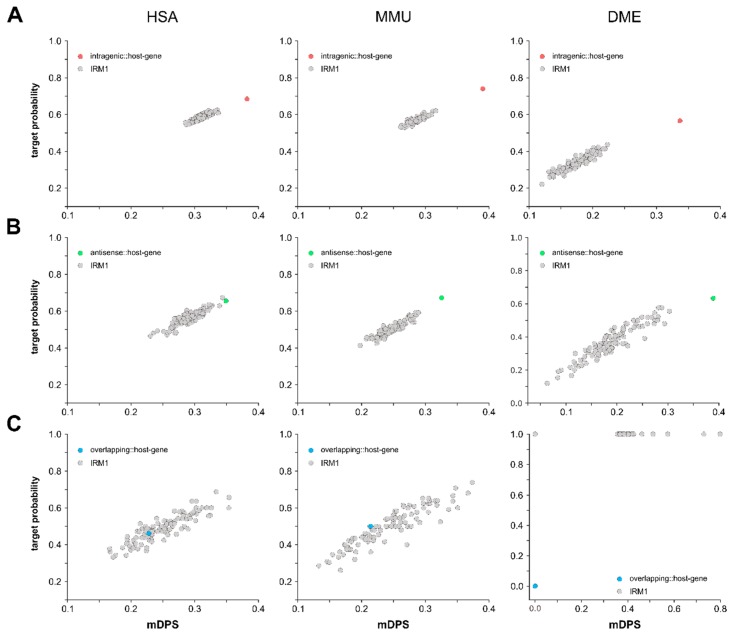
Intragenic (**A**), antisense (**B**), and overlapping (**C**) miRNA::gene targeting was assessed with DIANA’s microT-CDS v5 prediction tool (A–C). mDPS and target frequency was determined for the native groups (red, green, and blue dots) and compared to an iterative randomized model (IRM1), were each intragenic miRNA was randomly sampled against host genes 100 times (grey dots). For each iteration, the mean mDPS and the mean target frequency was determined, and Grubb’s outlier test was used to identify statistically significant allocation of the native group to the randomized model (A–C). The threshold for statistical significance was set to an α error of 0.05.

To elucidate a potential miRNA::host gene autoregulatory mechanism, we applied miRNA target predictions for the respective host genes using the microT-CDS algorithm. As both mature miRNA strands (i.e., -5p and -3p) could possibly target their host gene, we calculated a modified target prediction score (mDPS), in which the target probability for both strands was included in a weighted manner (see Methods section). As a second measure indicative of miRNA::host gene targeting, we calculated the target probability of all miRNAs with their respective host genes as average per species (Figure 5A–C). The respective average mDPS values and target probabilities are provided in Table 3. To test whether miRNA::host gene target probabilities and mDPS values were specific or occurred by chance due to overly permissive target predictions, we generated iterative randomized models (IRMs) for each species, by pairing the lists of each type of miRNAs with randomly assigned protein-coding genes (IRM1; 100 iterations). Outlier testing revealed that both the target probability, as well as the average mDPS of intragenic miRNAs for HSA, MMU, and DME, was significantly different from the IRM1 models (Figure 5A; Grubb’s test, *p* < 0.05), suggesting an evolutionary conserved autoregulatory pathway of miRNA::host gene regulation. Surprisingly, the antisense miRNA target probability and average mDPS for MMU and DME, but not HSA, was also significantly different from the respective IRM1 models (Figure 5B), suggesting a regulatory role not only of the miRNAs located on the sense strand but also of miRNAs located on the antisense strand of a gene. In contrast, overlapping miRNAs did not show specific targeting of their partially hosting genes (Figure 5C).

**Figure 6:**
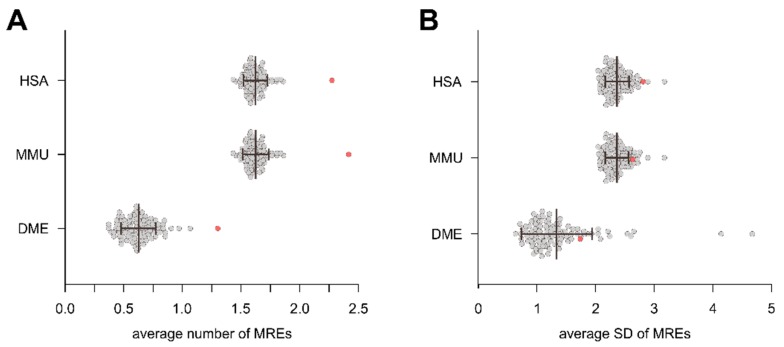
The number of harbored MREs targeted by the intragenic miRNA was compared to an additional IRM3 that randomly associated genes to intragenic miRNAs. (**A**) The mean MRE number for each iteration was determined and compared to the native intragenic miRNA::host gene group using Grubb’s outlier test. (**B**) The standard deviation of the average MRE number per iteration was not different for intragenic miRNA::host gene pairs and IRM modeled data. The threshold of statistical significance was set to <0.05.

A possible explanation for the increased target probability and mDPS values for intragenic miRNAs and their host genes could be an increase in the number of miRNA recognition elements (MREs) on the host gene mRNA specific for the respective intragenic miRNAs. We, therefore, evaluated the number of host gene MREs for the respective intragenic miRNAs, as well as from the randomly sampled IRM1 models. The average number of MREs for intragenic miRNAs on their host genes was significantly higher than on random genes for HSA, MMU, and DME (Figure 6A; average number of MREs, HSA—2.05, MMU—2.18, DME—1.30). To ensure that the analysis of MREs was not confounded by variance differences between the individual iterations and the native intragenic miRNA::host gene values, we determined the standard deviation (SD) for the iterations and the native values and found no significant difference for any of the species (Figure 6B).

**Figure 7:**
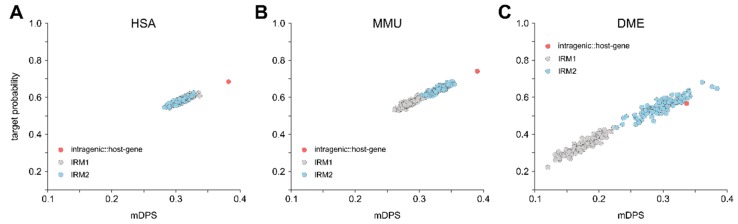
Host gene specificity was assessed by a second IRM, where each host gene was randomly sampled against a non-intragenic miRNA for 100 iterations. For each iteration, the mDPS and the mean target frequency were determined (sky blue dots) and compared to the native groups (red dots) and the IRM1 (grey dot cloud) for HSA (**A**), MMU (**B**) and DME (**C**). Grubb’s outlier test was used to assess the statistical allocation of the native group to the IRM2 + IRM1. The threshold of statistical significance was set to <0.05.

Although both the intragenic miRNA::host gene target probability and mDPS values, as well as the number of MREs specific for the respective intragenic miRNA was increased, a strong limitation of our model of an autoregulatory role of intragenic miRNAs would occur if the respective host genes were generally targeted by more miRNAs, independent of their type. Thus, to determine the specificity of host genes for their respective intragenic miRNAs, we generated an additional IRM that paired the host gene with randomly assigned non-intragenic miRNAs (IRM2; 100 iterations). The average target probability and mDPS values for individual iterations were determined and compared to IRM1 as well as the intragenic miRNA::host gene pairs. For both HSA and MMU, but not DME, the intragenic miRNA::host gene pair was significantly different from the IRM2 models (Figure 7A–C), suggesting high-level specificity of host genes for their intragenic miRNAs. Interestingly, there seems to be a separation between IRM1 and IRM2 for DME, which is slightly less obvious in MMU, and absent in HSA (Figure 7A–C). These results thus are consistent with an evolutionary pressure on bidirectional intragenic miRNA::host gene specificity.

Finally, experimentally validated intragenic miRNA::host gene interactions for HSA and MMU were retrieved from TarBase, miRTarBase, and starBase and compared to the host gene target predictions. This revealed that a respectable number of miRNA::host gene interactions were already experimentally validated (107 validations for HSA and 44 validations for MMU), which further strengthens the concept of a specific autoregulatory interaction between miRNAs and their host genes.

### 3.4. Relation between Proximity to Gene and Target Probability

**Figure 8:**
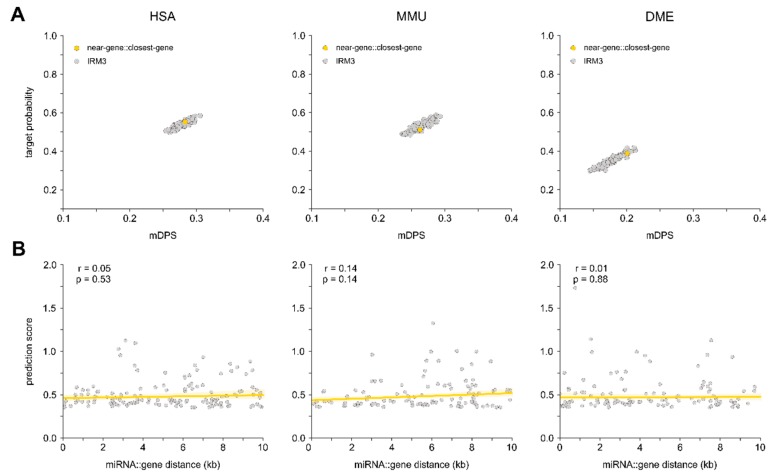
Extragenic miRNAs located 10kb up and downstream of a gene were defined as near-gene miRNAs. (**A**) Near-gene miRNA::closest-gene mDPS and target frequency (yellow dot) were determined and compared to an IRM, where each near-gene miRNA was randomly sampled against a protein-coding gene for 100 iterations (grey dot cloud). Grubb’s outlier test was used, and no significant allocation of the native group was identified. (**B**) Linear relationship between the mDPS and the distance of the miRNA to its closest gene was assessed, and the Pearson correlation coefficient, as well as the *p*-value, was determined for HSA, MMU, and DME.

We could so far show that intragenic miRNAs show an increased probability for targeting their own host genes, higher target prediction scores, and that host genes harbor more binding sites specific for the hosted miRNA. To evaluate whether this evolutionarily conserved interaction of miRNAs with their host genes is based on the placement of a miRNA within a gene, or if the simple distance of a miRNA to a gene is a determinant for autoregulation, we extended our analysis to miRNAs located in the vicinity of genes, specifically up to 10 kilobases up- and downstream of a gene (i.e., near-gene miRNAs; see Figure 1). These near-gene miRNAs underwent the same analysis as intragenic, antisense, and overlapping miRNAs. Near-gene miRNAs exhibited a significantly lower target probability for genes in their direct vicinity as compared to intragenic miRNAs for their host genes in HSA, MMU, and DME (Figure 8). Using IRM modeling, we found that for near-gene miRNAs, the probability of targeting genes in their vicinity or randomly selected genes was similar across all three species (Figure 8A; Grubb’s test *p* > 0.05). In addition, there was no significant correlation between the miRNA::gene distance and the mDPS suggesting no direct relationship between the miRNA::gene distance and the probability of mRNA targeting in HSA, MMU, and DME (Figure 8B). These data support the hypothesis that intragenic miRNAs possess the specific capability to directly autoregulate their host genes.

### 3.5. Indirect Host Gene Regulation by Modulation of Functional Pathways

In general, miRNAs target multiple different mRNAs, and host gene autoregulation might play an important but from a functional perspective minor role. Therefore, we investigated whether intragenic miRNAs are able to target other host genes within the networks, which were functionally linked to their host genes. Target gene analysis was performed for the entire set of intragenic miRNAs against all genes hosting intragenic miRNAs. This revealed a set of highly interactive intragenic miRNAs for both HSA and MMU that were predicted to target more than 75% of all host genes and, therefore, might play an important functional role in pathway regulation (Table 4). When comparing predicted miRNA::host gene interactions to the combined list of experimentally validated interactions, we found that in total 44.90% interactions for HSA (36,040 from 80,276 predicted, plus 8309 non-predicted) and 20.29% for MMU (17,024 of 83,887 predicted, plus 4362 non-predicted) have already been validated previously, however, not all intragenic miRNAs are represented in the above-used databases (HSA, 882 of 992 intragenic miRNAs; MMU, 278 of 705).

**Figure 9:**
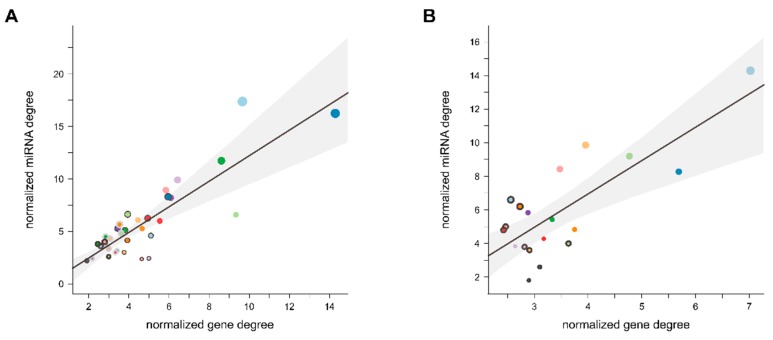
Indirect autoregulation of intragenic miRNAs for each intragenic host gene cluster in HSA (**A**) and MMU (**B**) was assessed. miTG prediction score for all possible intragenic miRNA::host gene interactions was determined and a combined network of host gene::host gene interaction and intragenic miRNA::host gene interaction was composed. Network analysis was performed, determining the degree centrality for each miRNA normalized to the total number of miRNA nodes and for each gene normalized to the total number of nodes in the network. A linear relationship between the mean normalized miRNA degree and the mean normalized gene degree for each cluster was assessed, and the summed target prediction score normalized to the total number of miRNA::gene interactions in each cluster is depicted as the dot-size for each cluster.

To investigate a possible indirect regulation of functional pathways, host gene networks from the previous PPI analysis were extended by intragenic miRNAs hosted by the respective cluster genes and targeting either their own host or other host genes within the same network. These connections were additionally weighted by the individual target prediction scores. Degree centrality for both intragenic miRNA network nodes (miRNA degree), as well as host gene nodes (gene degree), was calculated by summing the number of incoming network edges per node, averaged and normalized per cluster based on the number of miRNA nodes (for miRNA degree) or the number of total nodes (miRNA nodes and gene nodes for gene degree). Plotting the normalized miRNA degree as a function of the normalized gene degree, followed by linear regression analysis, resulted in a separation into clusters with high miRNA regulatory strength and clusters with low miRNA regulatory strength (Figure 9). The clusters with the highest proposed regulatory strength (HSA: Cluster 2; MMU: Cluster 1; see Supplementary Figures S3 and S5) were both associated with ubiquitin-related pathways, suggesting that the function of the genes related to ubiquitin-related clusters is highly regulated by miRNAs co-expressed with the respective genes and this regulation appears to be conserved at least between mammals.

To further test this indirect regulatory mechanism not only on the level of host genes but on the entire interactome, we performed community detection on the complete StringDB database for human and mouse and selected the ones containing at least five genes and five intragenic miRNAs. This approach identified 26 communities for HSA, and 28 communities for MMU, which contained host genes. Subsequently, each gene per community was tested for experimentally validated intragenic miRNA targeting that was harbored within the same community. The number of validations was then normalized to the total number of genes found in the community, the total number of miRNAs in the community, as well as the overall number of all possible interactions in the community (Supplementary Tables S4 and S5).

In total, 24 of 26 (HSA) and 26 of 28 (MMU) communities were influenced by intragenic miRNAs hosted within the same community (interaction ratio > 0). Influenced communities were ranked based on the interaction ratio, to identify communities with a high indirect autoregulatory potential by intragenic miRNAs. This revealed 12 (HSA) and 10 communities (MMU) with an interaction ratio ≥ 1. Functionality of these communities was further assessed by pathway enrichment analysis, showing that for HSA biological processes, such as the ribosomal large subunit assembly (community 11), regulation of alternative mRNA splicing (community 8), Rab protein signal transduction (community 4), positive regulation of I-kappaB kinase/NF-kappaB signaling (community 7) and ubiquitin-related pathways (community 12), for MMU biological processes, such as regulation of translation (community 66), positive regulation of cAMP-mediated signaling (community 1) and ubiquitin-related pathways (community 5), were significantly enriched (Supplementary Tables S6 and S7). Additionally, comparing the enrichments for all communities between HSA and MMU revealed that 72.81% of the enrichments were identical, suggesting conservation of indirect autoregulated pathways.

**Figure 10:**
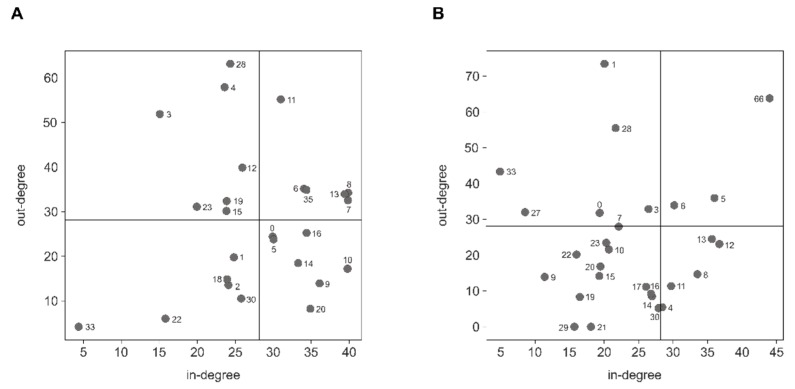
Cross-community regulation by intragenic miRNAs was assessed for HSA (**A**) and MMU (**B**). In- and outdegree for each community was assessed by the overall number of in- and outgoing edges considering the edges weight (interaction ratio). Communities were scattered in four different quadrants, which are separated based on the overall average in- and outdegree for each community.

Since miRNAs are in general predicted to target a large number of mRNAs, cross-community regulation by miRNAs was assessed by investigating the number of experimentally validated intragenic miRNA::gene interactions between the different communities. Network analysis was performed to determine the in- and out-degree for each community based on the overall number of incoming and outgoing validated miRNA::gene interactions, as well as the weighted strength (interaction ratio). This revealed communities whose intragenic miRNAs showed high regulatory potency without being strongly regulated by other communities (Figure 10A,B, upper left quadrant), communities that showed strong regulation by other communities without having high regulatory potency on other communities (lower right quadrant), as well as communities that showed strong bidirectional regulation (upper right quadrant) or both low regulatory potency and being barely regulated (lower left quadrant; Supplementary Tables S7 and S8).

## 4. Discussion

This study provides new knowledge on the localization of miRNAs in relation to protein-coding and non-coding genes, specifically to their host genes, and highlights miRNA::host gene interactions as a specific and evolutionarily conserved autoregulatory mechanism. The results support the concept that a given intragenic miRNA not only interacts with its host gene but also has a high probability of influencing other members of the host gene’s interactome, which places miRNAs in the role of a master switch, capable of regulating their host genes entire protein interaction cluster.

### 4.1. Distribution of Intragenic miRNAs Across Different Species

The percentage of intragenic and intronic miRNAs based on the location within protein-coding genes was slightly higher than in previous reports, where an increased percentage of known miRNA genes located within annotated protein-coding regions from 42% to 57% was observed with an increasing number of datasets available and progressing miRBase releases [4,47]. Within the population of human intragenic miRNAs, however, the percentage of intronic, exonic, and mixed miRNAs was the same as described in a previous study, although at that time, only 221 human miRNAs were known [2]. Thus, the extended data set, including all currently known miRNAs across four species, further supports the concept that many intragenic miRNAs and their host genes resemble single transcription units. However, biogenesis of intragenic RNAs might well occur independently from their host gene [49]. Not all types of intragenic miRNAs may have the same co-expression bias. The probability of co-expression of intragenic miRNA with their host genes is strongly related to their evolutionary conservation degree, and evolutionarily conserved intragenic miRNAs have a much higher rate of co-expression with host genes than poorly conserved ones [26]. In line with the concept that intragenic miRNAs provide an evolutionary advantage, the values were quite similar for human and mouse which have the most complete sets of annotated coding genes and miRNAs, however, diverging for other species, such as drosophila melanogaster or zebrafish. The differences, however, may also be at least partially due to the fact that for less frequently studied species, lower numbers of stringent and complete data sets are available.

### 4.2. miRNA Host Genes Show Functional Clusters and Pathway Enrichment

Up till now, miRNAs targeting specific gene clusters are largely exploited from a biomarker angle, i.e., as predictors for disease or responsiveness to certain therapies. In contrast, miRNA host genes as components of enriched pathways and the mutual interactions of proteins encoded by mRNAs harboring miRNAs have so far received only a little attention from a global point of view. Significant network communities are enriched with target genes of miRNA clusters, as suggested for a network community of ten proteins, which is co-regulated by four miRNA families in the *mir-379* cluster [50].

### 4.3. Intragenic miRNAs Target Their Host Genes

Computational tools and databases, such as TargetScan [51], miRanda [52], PicTar [53], RNA22 [54], PITA [55], miRecords [56], miRTarBase [44], miRTrail [57], and miRnalyze [58] are available to address the complex relationship between miRNAs and their mRNA targets. Other databases, such as DIANA miRPath v3 [59], miRPathDB [60], miTalos v2 [61], MiRSEA [62], or Subpathway-GMir [63] focus on associations of miRNAs with specific pathways. Unfortunately, such tools exhibit a considerable number of false positive and false negative results as that they are mostly based on computational predictions and probabilistic models, which often hampers experimental validation. Significantly different results are obtained when enrichment analysis is applied on predicted targets or validated targets, and computational predictions may overestimate the number and extent of meaningful miRNA-regulated processes [41,42]. To overcome the limitations of in silico approaches and to address the issue of “translational paresis” from theory to practice, databases collating information on miRNAs, functions and disease associations are emerging, such as HMDD [64], miRwayDB [65], PhenomiR [66] and miRPathDB [60].

Both host genes and intragenic miRNAs are subject to different regulatory mechanisms, and tight regulatory control of these genes is critical for specific molecular pathways and cellular functions. Therefore, potential feedback loops between intragenic miRNAs and their host genes, when targeted by the hosted miRNA, may be critically important. To this end, we performed genome-wide analyses and found strong interaction patterns observed between the targeting of host genes by intragenic miRNAs, but not extragenic or overlapping miRNAs. In a previous study, 20% of intragenic miRNAs were predicted to target their host mRNA transcript, and 22 of the 74 pathways yield overrepresentation of proteins encoded by mRNA targets of associated intragenic miRNAs [47]. In our in silico paradigm, intragenic miRNAs showed increased probability for targeting their host genes and higher target prediction scores. Together, this provides novel unbiased evidence for autoregulatory miRNA::host gene interactions as a general principle that fine-tunes host gene expression. Experimentally validated intragenic miRNA::host gene interactions for HSA and MMU were retrieved and revealed that 107 of HSA and 44 of MMU miRNA::host gene interactions are already experimentally validated. For miRNAs regulating their host genes interactomes, even 44.90% of interactions for HSA and 20.29% for MMU are validated. This further strengthens the concept of a specific autoregulatory interaction between miRNAs and their host genes with significant functional relevance.

Our current analysis supports a regulatory role not only of the miRNAs located on the sense strand but also of miRNAs located on the antisense strand of a gene. In contrast, overlapping miRNAs did not show specific targeting of their partially hosting genes. As a possible explanation for the increased target probability and mDPS values for intragenic miRNAs and their host genes, we found an increase in the number of miRNA recognition elements (MREs) on the host gene mRNA specific for the respective intragenic miRNAs. While intragenic and overlapping miRNAs showed a clearly significant probability to target their host genes, near-gene miRNAs exhibited a significantly lower target probability for genes in their direct vicinity as compared to intragenic miRNAs for their host genes. Therefore, the proximity of a miRNA to a gene seems not to be sufficient to warrant a high probability for a given miRNA to target a near gene.

Interestingly, not only miRNAs are located in genomic regions but also other non-coding RNAs (e.g., snoRNAs, lncRNAs, pseudogenes, circRNAs, etc.) are embedded in host genes, which are adopting the same strategy as intragenic miRNAs and have been reported to interfere with host gene expression [67,68,69,70].

A particularly relevant subgroup of intragenic miRNA are alternatively spliced miRNAs located at exon–intron junctions due to their specific inhibitory effects on host gene expression [71,72,73,74,75]. This respective “mixed” miRNA subpopulation suitable for alternative splicing was in the same range in a previous study reporting a similarly lower subpopulation of these miRNAs in human compared to mouse [76]. Furthermore, it has been shown that transcription of intronic miRNAs, as well as splicing of the host genes, can bidirectionally regulate each other’s transcription by a counteracting mechanism, where transcription of the miRNA reduces the splicing of the host gene and vice versa [77,78].

### 4.4. Indirect Host Gene Regulation by Modulation of Functional Pathways

While previous studies mainly focused on conserved miRNA expression from an evolutionary angle, we here emphasize the central functional role of conserved miRNAs for the regulation of their host genes together with other genes within the host gene’s interactome by providing evidence that intragenic miRNAs can target other genes within the networks functionally linked to their host genes. Intragenic miRNAs are particularly frequent in genes encoding proteins involved in pathways that are relevant for highly specialized organs, such as the frontal cortex [24]. In general, it is suggested that target genes regulated by an individual miRNA interact through a network of functionally associated genes [79,80], and miRNA interactome analysis has been introduced as a tool to identify miRNA functional relevance [81,82]. Using the network analysis toolkit ANAT [83], Melamed, Levy (76) generated a network connecting the most abundant AGO2-mRNA targets, in which six of ten candidate targets are part of a potential biological network. Enrichment analysis of the reconstructed network showed that both potential miR-412 targets, as well as their associated intermediate node genes, were overrepresented in programmed cell death processes [76]. Furthermore, miRNA::target gene interaction sites contain both interactor and structural elements in various combinations. Not considering spatially defined structural elements, such as precursor miRNAs and the interactor mRNAs or long ncRNAs, our approach based on sequence complementarity may not consider all relevant types of miRNA interactions with their host genes as relevant targets. This may explain why performance of bioinformatics studies based on nucleotide sequences cannot be sufficiently precise on its own. Additional parameters, such as three-dimensional structures and binding energy of specific miRNA motifs, need to be taken into account as they become available for a full understanding of miRNA::host gene autoregulation [84]. However, and in line with studies reporting that individual miRNAs target disease-relevant molecular pathways [60,64,66] our unbiased genome-wide study provides important evidence that network-wide targeting is not the result of random associations but more likely resembles a generic mechanism regulating entire molecular pathways [85].

## 5. Conclusions

We demonstrate that intragenic miRNAs show a strong probability of interacting with their own host genes. This did not only involve direct miRNA/host gene targeting but also interaction with functionally related genes via an indirect autoregulatory mechanism. This strongly argues for the biological relevance of a potential autoregulation not only for the host genes itself but, more importantly, for the entire cluster of genes coding for proteins it interacts with.

## Figures and Tables

**Table 1:** Distribution of intragenic, antisense and overlapping miRNAs for protein coding and all genes.

	HSA	MMU	DME	DRE
	**Protein Coding Genes**			
intragenic	52.38	58.16	52.33	23.36
antisense	12.08	11.66	12.02	9.12
overlapping	0.31	0.33	0.78	0.28
intronic	88.63	79.86	76.03	95.52
exonic	6.77	9.38	19.01	4.48
mixed	4.59	10.76	4.96	0.00
	**All Genes**			
intragenic	66.70	68.52	70.93	34.76
antisense	19.45	15.58	13.95	11.97
overlapping	0.84	2.20	1.16	1.42
intronic	79.98	75.04	59.75	75.51
exonic	9.74	13.68	34.59	18.37
mixed	10.27	11.28	5.66	6.12

**Table 2:** Number of essential and non-essential host genes.

		Essential	Non-Essential	OGEE Essential	OGEE Non-Essential	Chi^2^	*p*-Value
**HSA**	intragenic	444	493	7168	14,398	79.47	<0.0001
	antisense	81	133	7168	14,398	1.82	>0.05
	overlapping	3	3	7168	14,398	-	-
**MMU**	intragenic	223	391	4341	4701	31.06	<0.001
	antisense	30	91	4341	4701	24.87	<0.001
	overlapping	1	3	4341	4701	-	-
**DME**	intragenic	8	119	408	13,810	4.11	0.043
	antisense	2	20	408	13,810	1.22	0.27
	overlapping	0	3	408	13,810	-	-

**Table 3:** Average mDPS values and target probabilities for intragenic miRNAs and host genes.

	HSA	MMU	DME
	**Average mDPS**		
intragenic	0.38	0.39	0.34
antisense	0.35	0.33	0.39
overlapping	0.23	0.21	0.00
near-gene	0.28	0.26	0.20
	**Target Probability**		
intragenic	68.41	74.07	56.59
antisense	66.09	67.20	67.86
overlapping	46.15	50.00	0.00
near-gene	55.51	51.44	38.83

**Table 4:** List of Top 10 interactive intragenic miRNAs.

	miRNA Name	# Edges	Average miTG Score
**HSA**	hsa-miR-766-3p	748	0.53
	hsa-miR-5193	743	0.56
	hsa-miR-761	738	0.57
	hsa-miR-4731-5p	735	0.55
	hsa-miR-6512-3p	733	0.53
	hsa-miR-338-3p	730	0.51
	hsa-miR-6764-5p	730	0.53
	hsa-miR-1237-3p	727	0.53
	hsa-miR-942-5p	725	0.57
	hsa-miR-6736-3p	719	0.56
**MMU**	mmu-miR-7033-5p	512	0.56
	mmu-miR-330-5p	489	0.51
	mmu-miR-6904-5p	484	0.52
	mmu-miR-1968-5p	481	0.51
	mmu-miR-6914-5p	478	0.51
	mmu-miR-6945-5p	476	0.51
	mmu-miR-6958-3p	476	0.52
	mmu-miR-7064-5p	474	0.52
	mmu-miR-6937-3p	473	0.51
	mmu-miR-6946-3p	472	0.61

## Data Availability

The data used for analysis are deposited in the publicly available databases Ensembl [36], miRBase [27], TarBase [43], miRTarBase [44], microT-CDS [40], starBase [45], g:Profiler [38] and String-db [37]. Additional datasets and analysis files are available from the corresponding author upon request.

## Supplementary Materials

The following are available online at https://www.mdpi.com/2073-4409/9/1/232/s1, Figure S1: Flow chart of data acquisition and processing. Validation databases used: starBase, miRTarBase, TarBase (yellow hexagons), Figure S2: StringDB protein–protein Markov chain cluster analysis of HSA intragenic miRNA host genes. 35 cluster were defined with a number of connected nodes >5, Figure S3: All HSA network-cluster were subject to a g:Profiler enrichment analysis for GO:Biological Process (A), GO:Cellulare Components (B), GO:Molecular Function (C) and KEGG (D), Figure S4: StringDB protein–protein Markov chain cluster analysis of MMU intragenic miRNA host genes, Figure S5: All MMU network-cluster were subject to a g:Profiler enrichment analysis for GO:Biological Process (A), GO:Cellulare Components (B), GO:Molecular Function (C) and KEGG (D), Figure S6: StringDB protein–protein Markov-chain cluster analysis on DME intragenic miRNA host genes, Figure S7: All DME network-cluster were subject to a g:Profiler enrichment analysis for GO:Biological Process (A) and GO:Cellulare Components (B), Table S1: List of species miRNA groups and classifications from Ensembl genome catalog, Tables S2: Network statistics for protein–protein interaction enrichments, Tables S3. Essential vs. non-essential genes for the different miRNA types and species, Tables S4: Indirect autoregulation of host gene related protein–protein networks by HSA intragenic miRNAs, Tables S5: Indirect autoregulation of host gene related protein–protein networks by MMU intragenic miRNAs, Tables S6: Enrichment Table for each separate community in HSA, ranked by the *p*-value, Tables S7: Enrichment Table for each separate community in MMU, ranked by the *p*-value, Table S8: In- and out-degree for each community in the HSA network, Table S9. In- and out-degree for each community in the MMU network.
