# Supplementary material for: Intragenic MicroRNAs Autoregulate Their Host Genes in Both Direct and Indirect Ways—A Cross-Species Analysis
Source: Cells. 2020 Jan 17;9(1):232. doi: 10.3390/cells9010232 (PMC7016697; doi:10.3390/cells9010232)
Supplement: Supplementary file 1 [file cells-09-00232-s001.pdf]

Intragenic microRNAs autoregulate their host genes in both direct and indirect ways – a cross-species analysis

Maximilian Zeidler[1*], Alexander Hüttenhofer[2], Michaela Kress[1] and Kai K. Kummer[1]

[1] Institute of Physiology, Medical University of Innsbruck, Innsbruck, Austria.

[2] Institute of Genomics and RNomics, Medical University of Innsbruck, Innsbruck, Austria.

SUPPLEMENTARY INFORMATION

Intragenic microRNAs autoregulate their host genes in both direct and indirect ways – a cross-species analysis

Maximilian Zeidler[1*], Alexander Hüttenhofer[2], Michaela Kress[1] and Kai K. Kummer[1]

[1] Institute of Physiology, Medical University of Innsbruck, Innsbruck, Austria.

[2] Institute of Genomics and RNomics, Medical University of Innsbruck, Innsbruck, Austria.

SUPPLEMENTARY INFORMATION

SUPPLEMENTARY FIGURE AND TABLE LEGENDS

Supplementary Figure S1: Flow chart of data acquisition and processing. Validation databases used: *starBase, miRTarBase, TarBase* (yellow hexagons).

Supplementary Figure S2: StringDB protein-protein Markov chain cluster analysis of HSA intragenic miRNA host genes. 35 cluster were defined with a number of connected nodes > 5.

Supplementary Figure S3: All HSA network-cluster were subject to a g:Profiler enrichment analysis for GO:Biological Process (A), GO:Cellulare Components (B), GO:Molecular Function (C) and KEGG (D). Significant enriched pathways were illustrated by the -log10(p-value).

Supplementary Figure S4: StringDB protein-protein Markov chain cluster analysis of MMU intragenic miRNA host genes. 19 cluster were defined with a number of connected nodes > 5.

Supplementary Figure S5: All MMU network-cluster were subject to a g:Profiler enrichment analysis for GO:Biological Process (A), GO:Cellulare Components (B), GO:Molecular Function (C) and KEGG (D). Significant enriched pathways were illustrated by the -log10(p-value).

Supplementary Figure S6: StringDB protein-protein Markov-chain cluster analysis on DME intragenic miRNA host genes. Two cluster were defined with a number of connected nodes > 5.

Supplementary Figure S7: All DME network-cluster were subject to a g:Profiler enrichment analysis for GO:Biological Process (A) and GO:Cellulare Components (B). Significant enriched pathways were illustrated by the -log10(p-value).

Supplementary Table S1. List of species miRNA groups and classifications from Ensembl genome catalog.

Supplementary Tables S2. Network statistics for protein-protein interaction enrichments.

Supplementary Tables S3. Essential vs. non-essential genes for the different miRNA types and species.

Supplementary Tables S4. Indirect autoregulation of host gene related protein-protein networks by HSA intragenic miRNAs.

Supplementary Tables S5. Indirect autoregulation of host gene related protein-protein networks by MMU intragenic miRNAs.

Supplementary Tables S6. Enrichment Table for each separate community in HSA, ranked by the p-value.

Supplementary Tables S7. Enrichment Table for each separate community in MMU, ranked by the p-value.

Supplementary Table S8. In- and out-degree for each community in the HSA network.

Supplementary Table S9. In- and out-degree for each community in the MMU network.

Intragenic microRNAs autoregulate their host genes in both direct and indirect ways – a cross-species analysis

Maximilian Zeidler[1*], Alexander Hüttenhofer[2], Michaela Kress[1] and Kai K. Kummer[1]

[1] Institute of Physiology, Medical University of Innsbruck, Innsbruck, Austria.

[2] Institute of Genomics and RNomics, Medical University of Innsbruck, Innsbruck, Austria.

SUPPLEMENTARY INFORMATION

SUPPLEMENTARY FIGURE AND TABLE LEGENDS

Supplementary Figure S1: Flow chart of data acquisition and processing. Validation databases used: *starBase, miRTarBase, TarBase* (yellow hexagons).

Supplementary Figure S2: StringDB protein-protein Markov chain cluster analysis of HSA intragenic miRNA host genes. 35 cluster were defined with a number of connected nodes > 5.

Supplementary Figure S3: All HSA network-cluster were subject to a g:Profiler enrichment analysis for GO:Biological Process (A), GO:Cellulare Components (B), GO:Molecular Function (C) and KEGG (D). Significant enriched pathways were illustrated by the -log10(p-value).

Supplementary Figure S4: StringDB protein-protein Markov chain cluster analysis of MMU intragenic miRNA host genes. 19 cluster were defined with a number of connected nodes > 5.

Supplementary Figure S5: All MMU network-cluster were subject to a g:Profiler enrichment analysis for GO:Biological Process (A), GO:Cellulare Components (B), GO:Molecular Function (C) and KEGG (D). Significant enriched pathways were illustrated by the -log10(p-value).

Supplementary Figure S6: StringDB protein-protein Markov-chain cluster analysis on DME intragenic miRNA host genes. Two cluster were defined with a number of connected nodes > 5.

Supplementary Figure S7: All DME network-cluster were subject to a g:Profiler enrichment analysis for GO:Biological Process (A) and GO:Cellulare Components (B). Significant enriched pathways were illustrated by the -log10(p-value).

Supplementary Table S1. List of species miRNA groups and classifications from Ensembl genome catalog.

Supplementary Tables S2. Network statistics for protein-protein interaction enrichments.

Supplementary Tables S3. Essential vs. non-essential genes for the different miRNA types and species.

Supplementary Tables S4. Indirect autoregulation of host gene related protein-protein networks by HSA intragenic miRNAs.

Supplementary Tables S5. Indirect autoregulation of host gene related protein-protein networks by MMU intragenic miRNAs**.**

Supplementary Tables S6. Enrichment Table for each separate community in HSA, ranked by the p-value.

Supplementary Tables S7. Enrichment Table for each separate community in MMU, ranked by the p-value.

Supplementary Table S8. In- and out-degree for each community in the HSA network.

Supplementary Table S9. In- and out-degree for each community in the MMU network.

Supplementary Figure S1

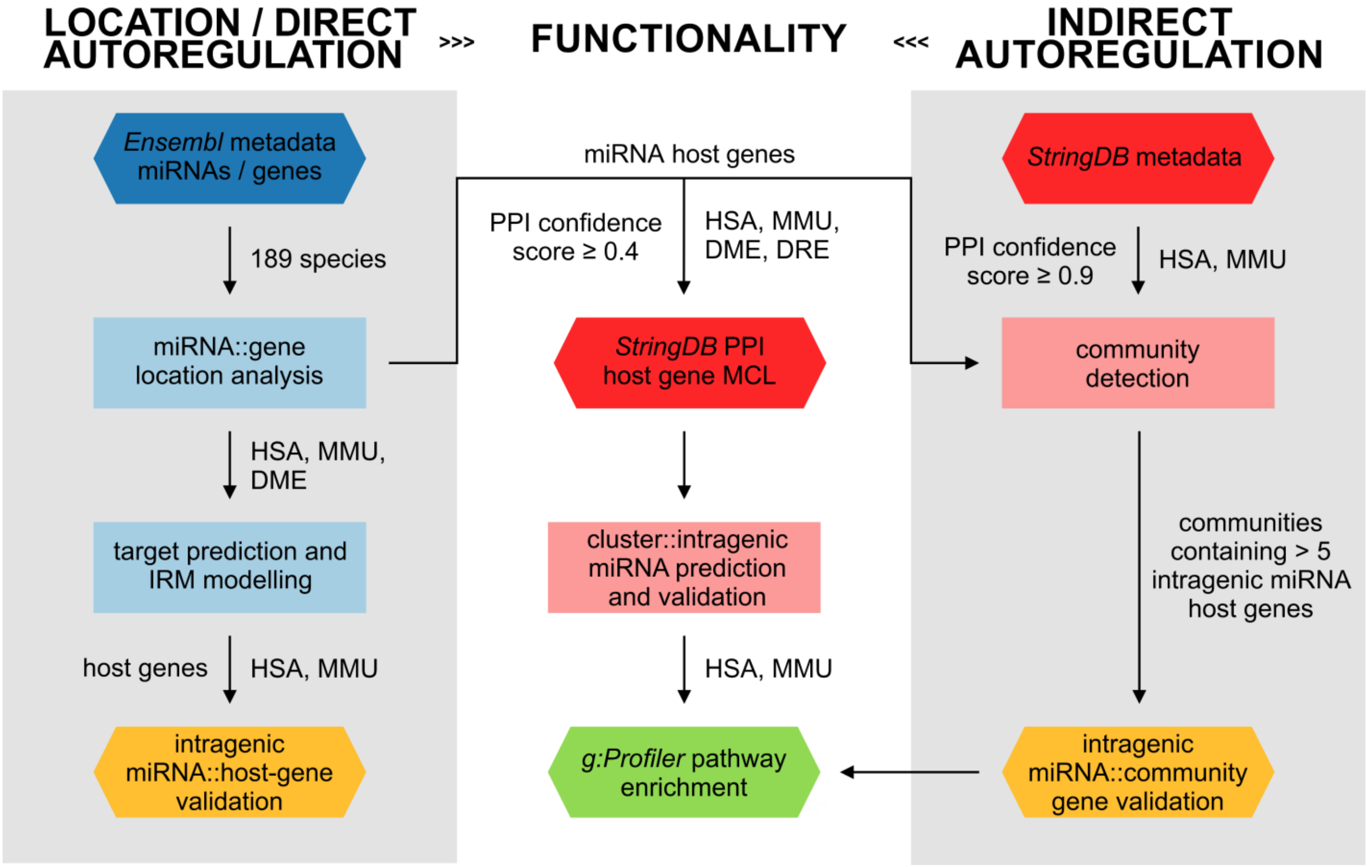

Supplementary Figure S2A

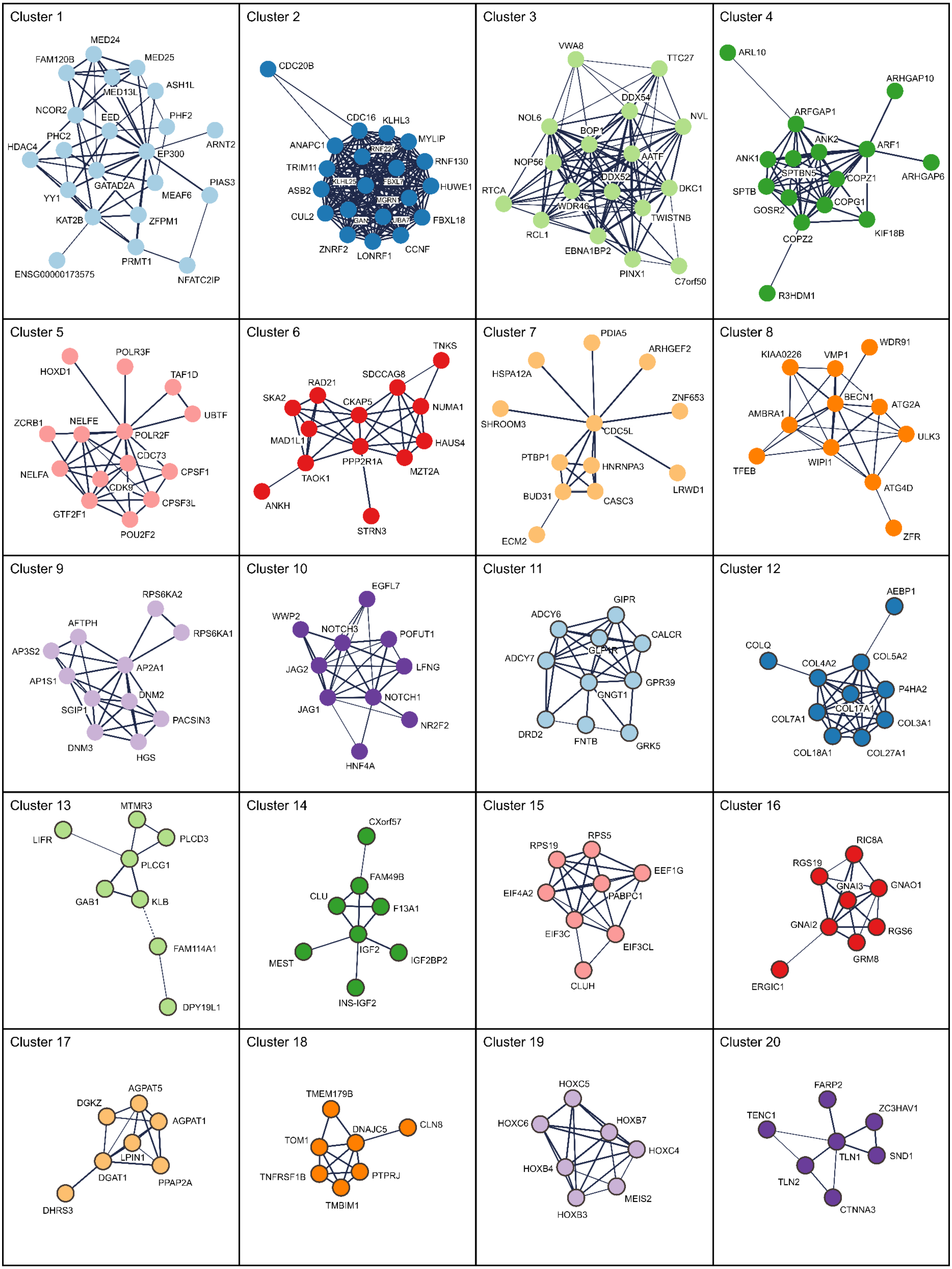

Supplementary Figure S2B

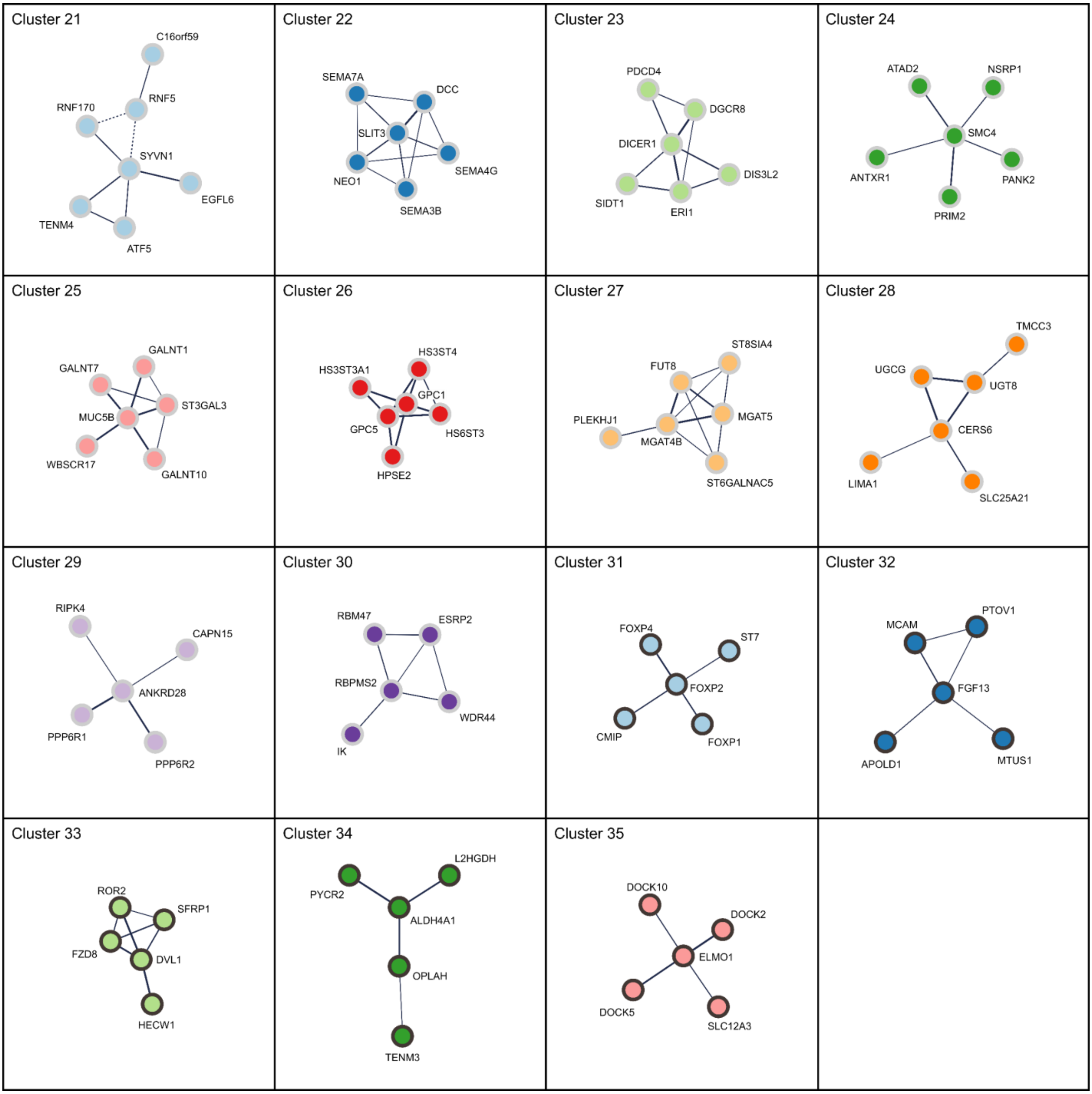

Supplementary Figure S3A

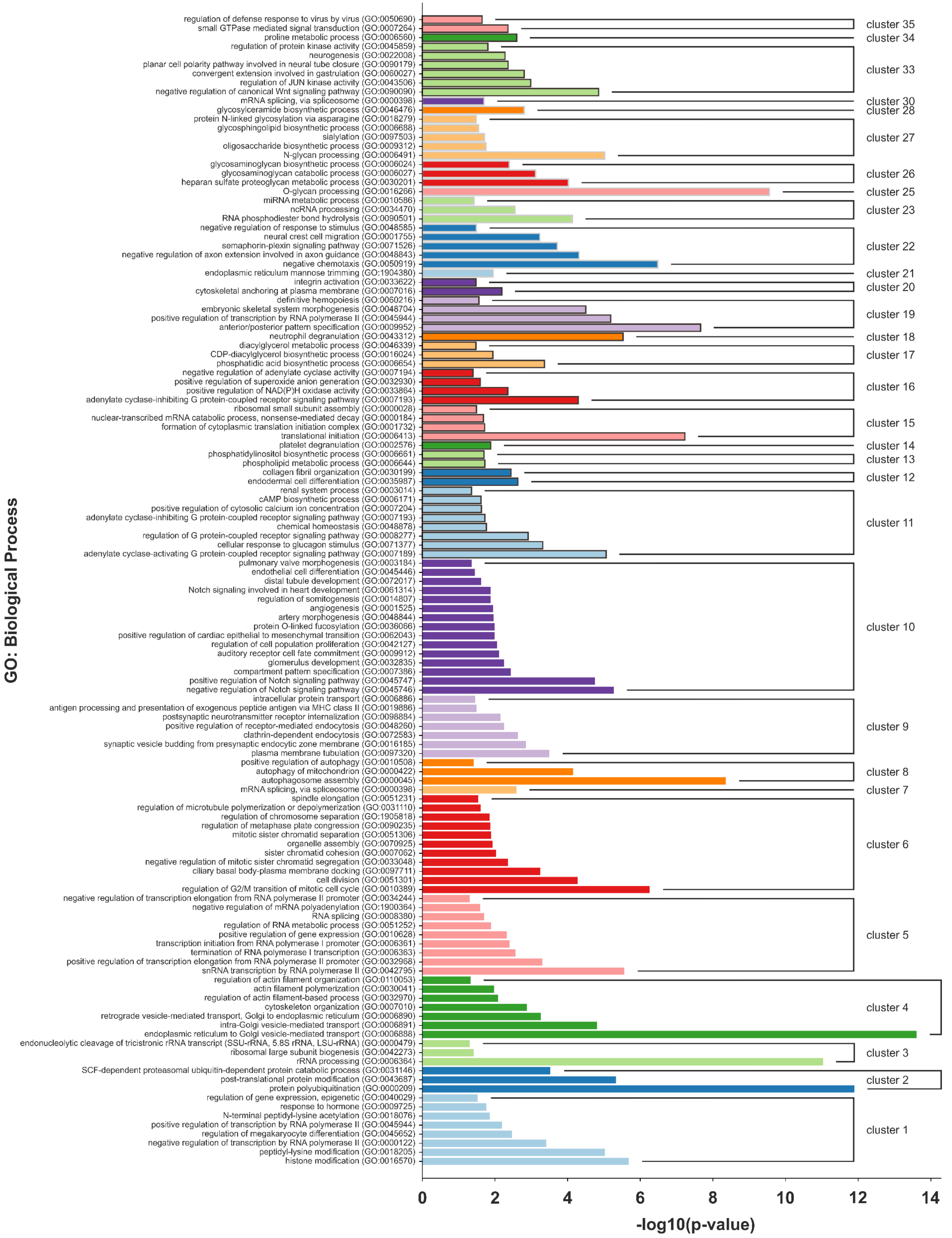

Supplementary Figure S3B

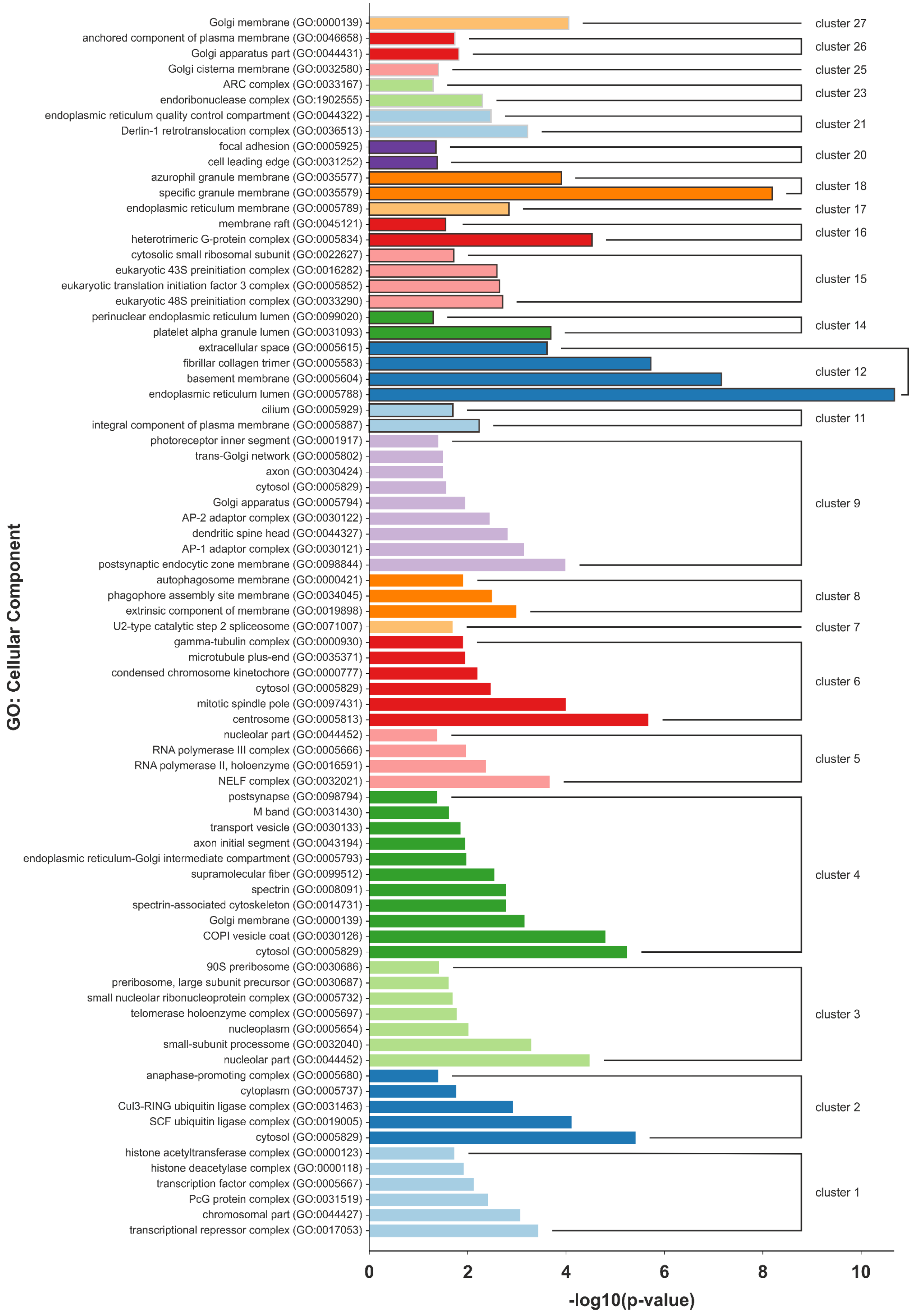

Supplementary Figure S3C

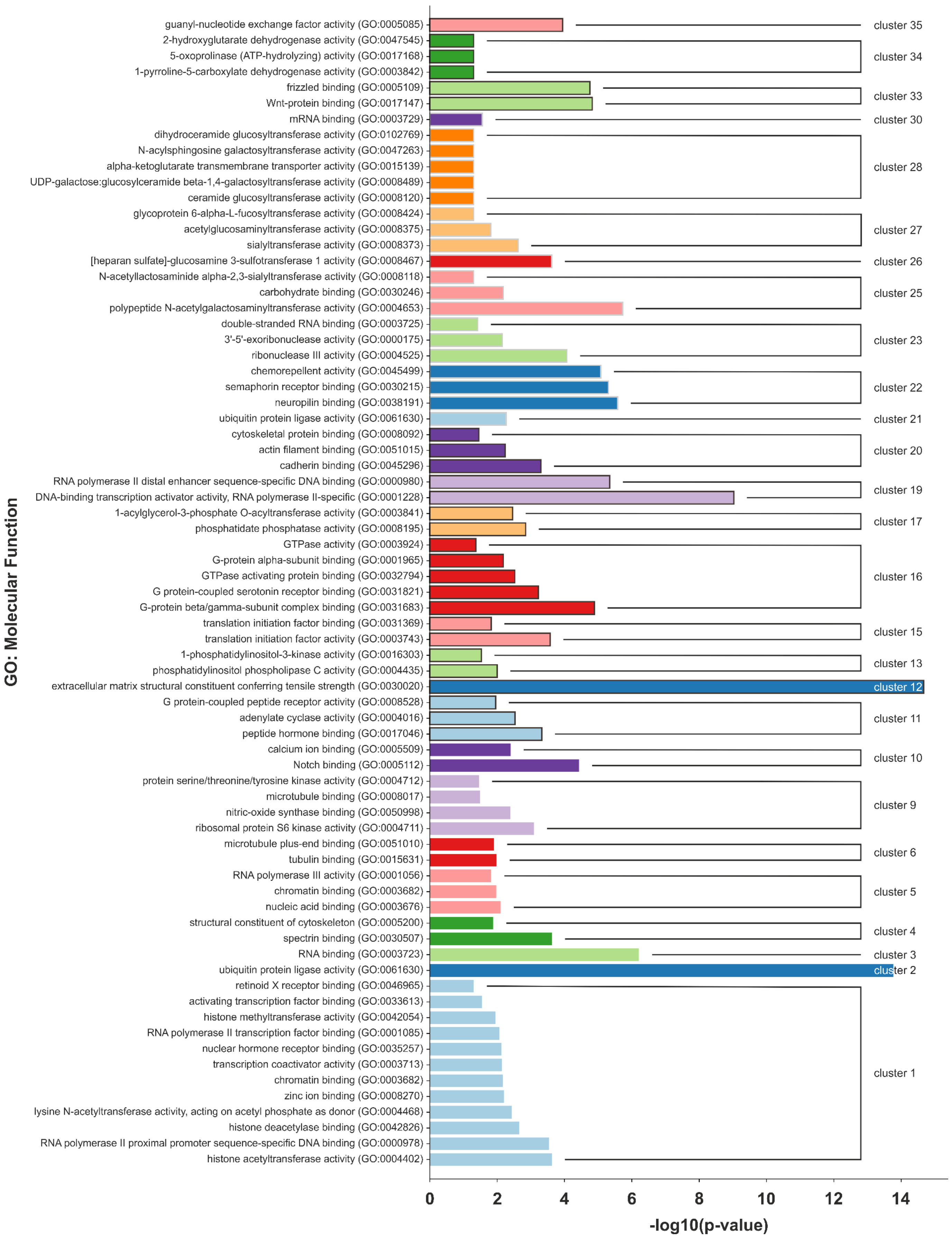

Supplementary Figure S3D

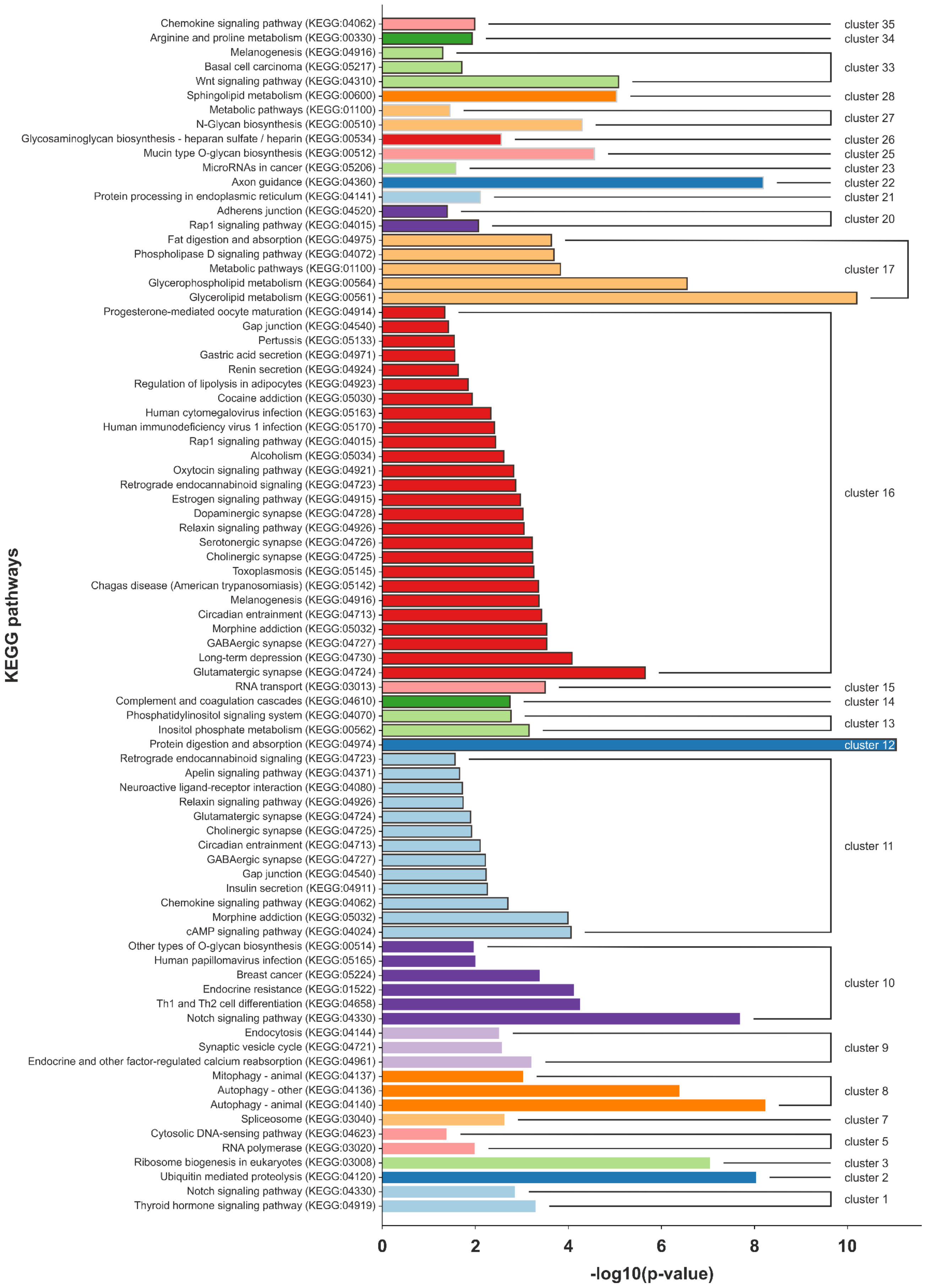

Supplementary Figure S4

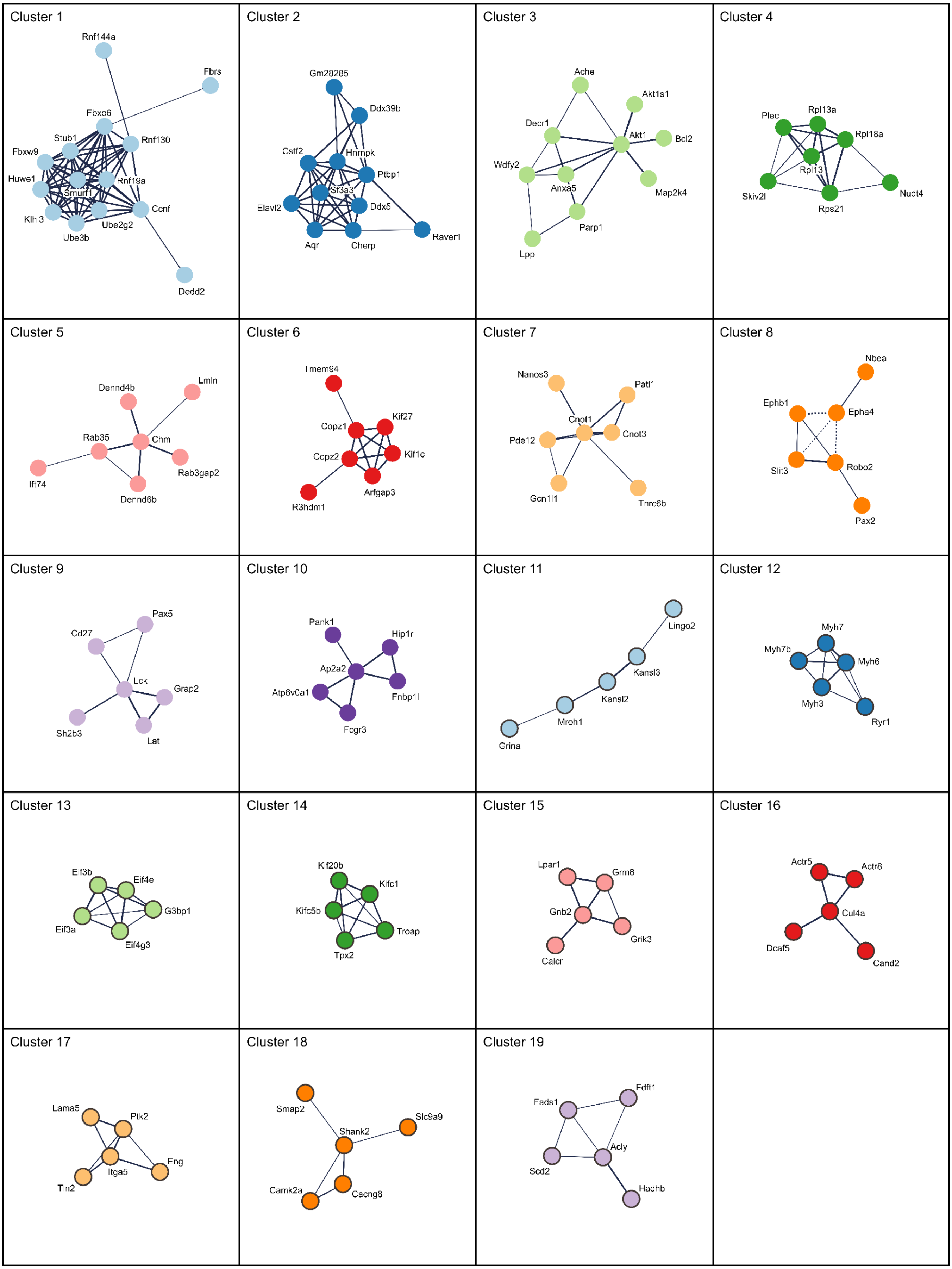

Supplementary Figure S5A

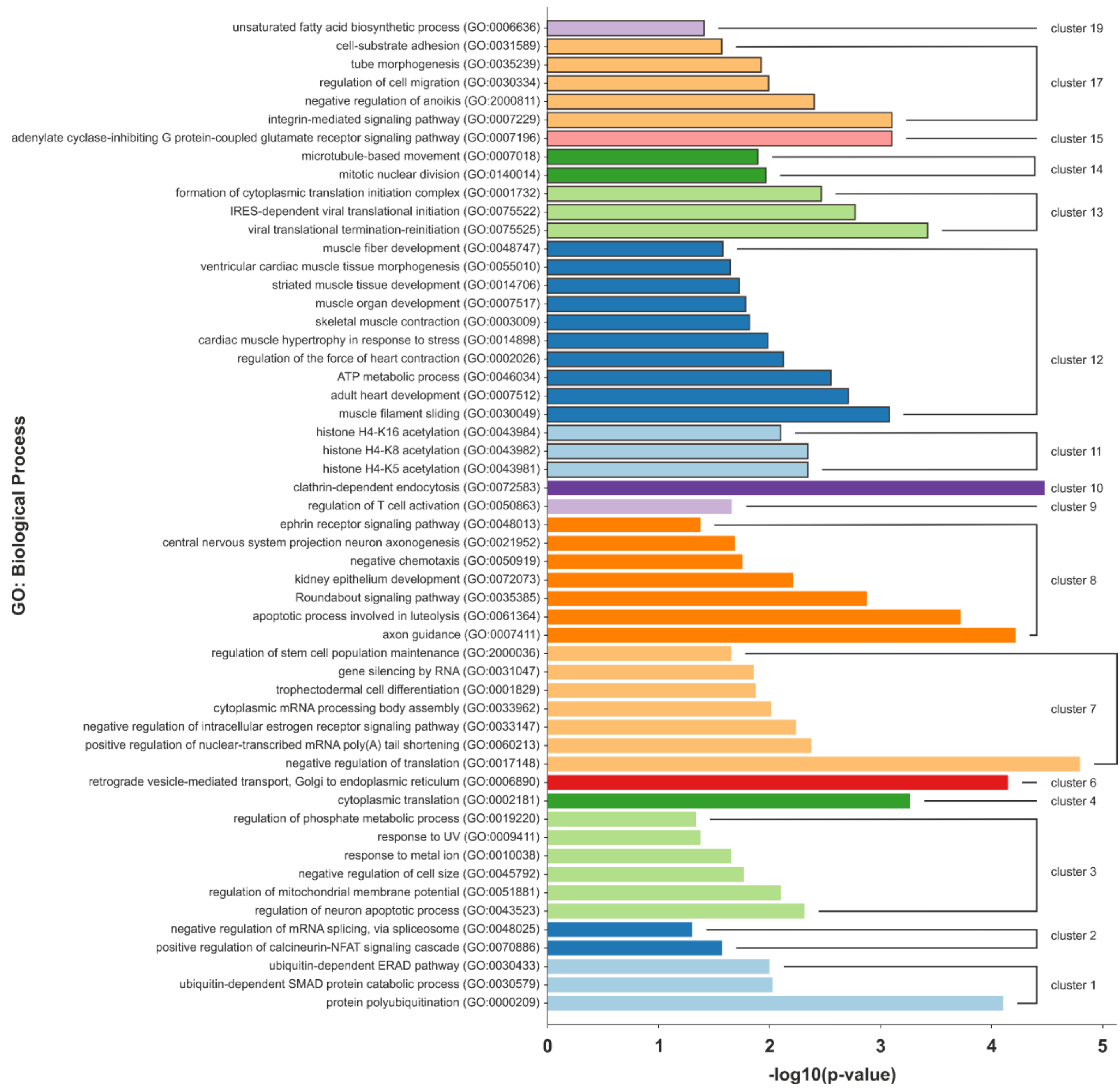

Supplementary Figure S5B

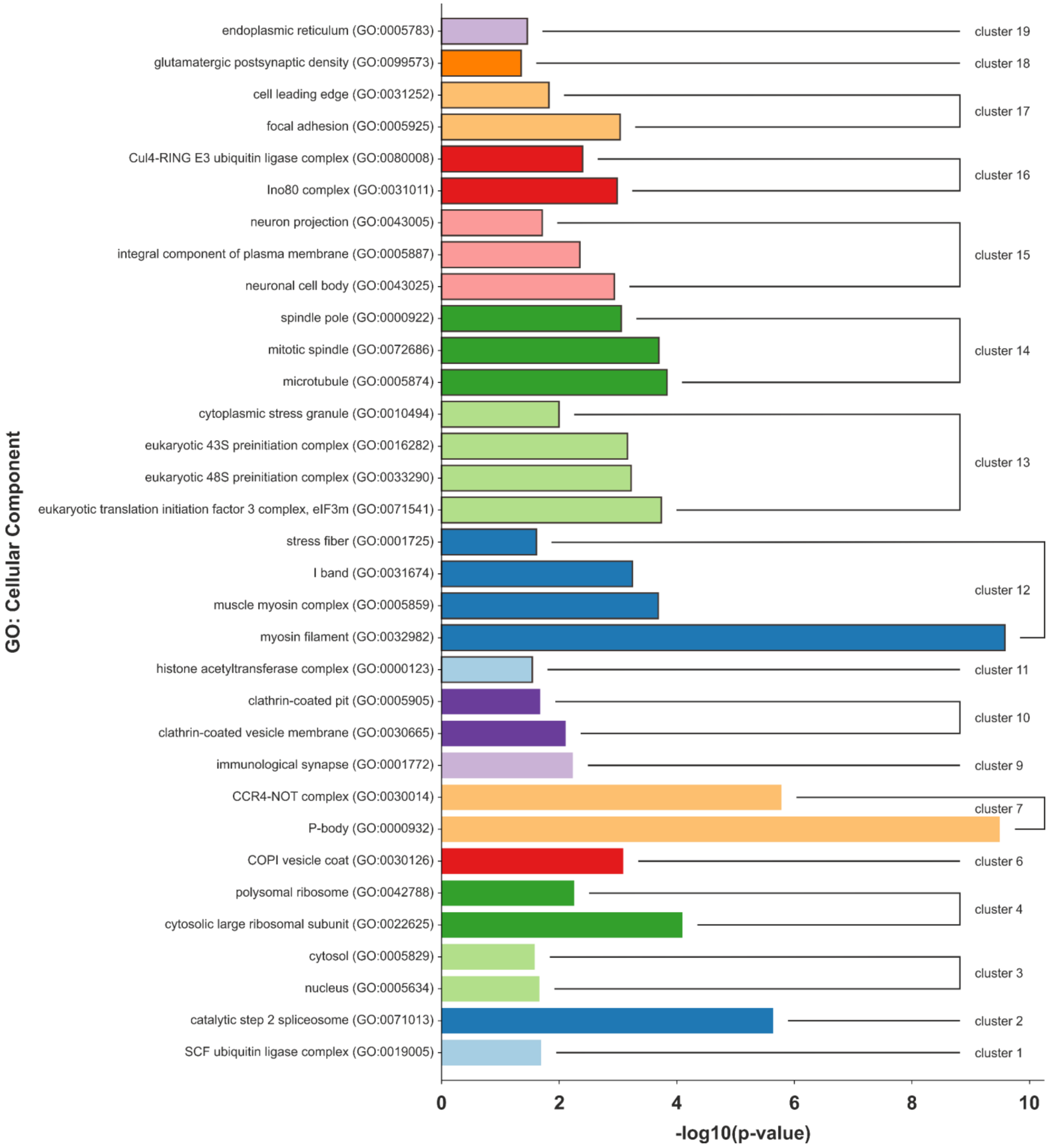

Supplementary Figure S5C

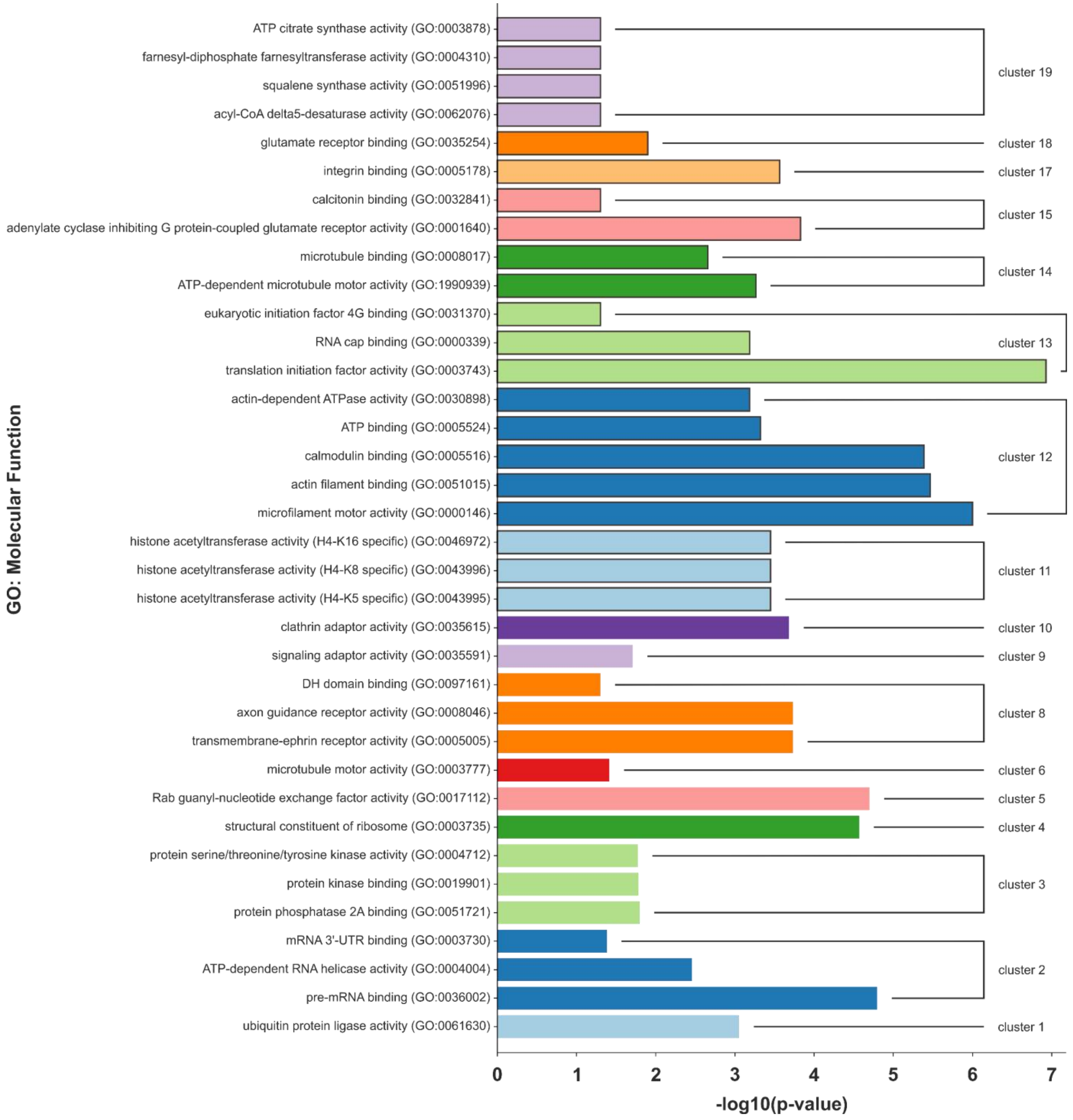

Supplementary Figure S5D

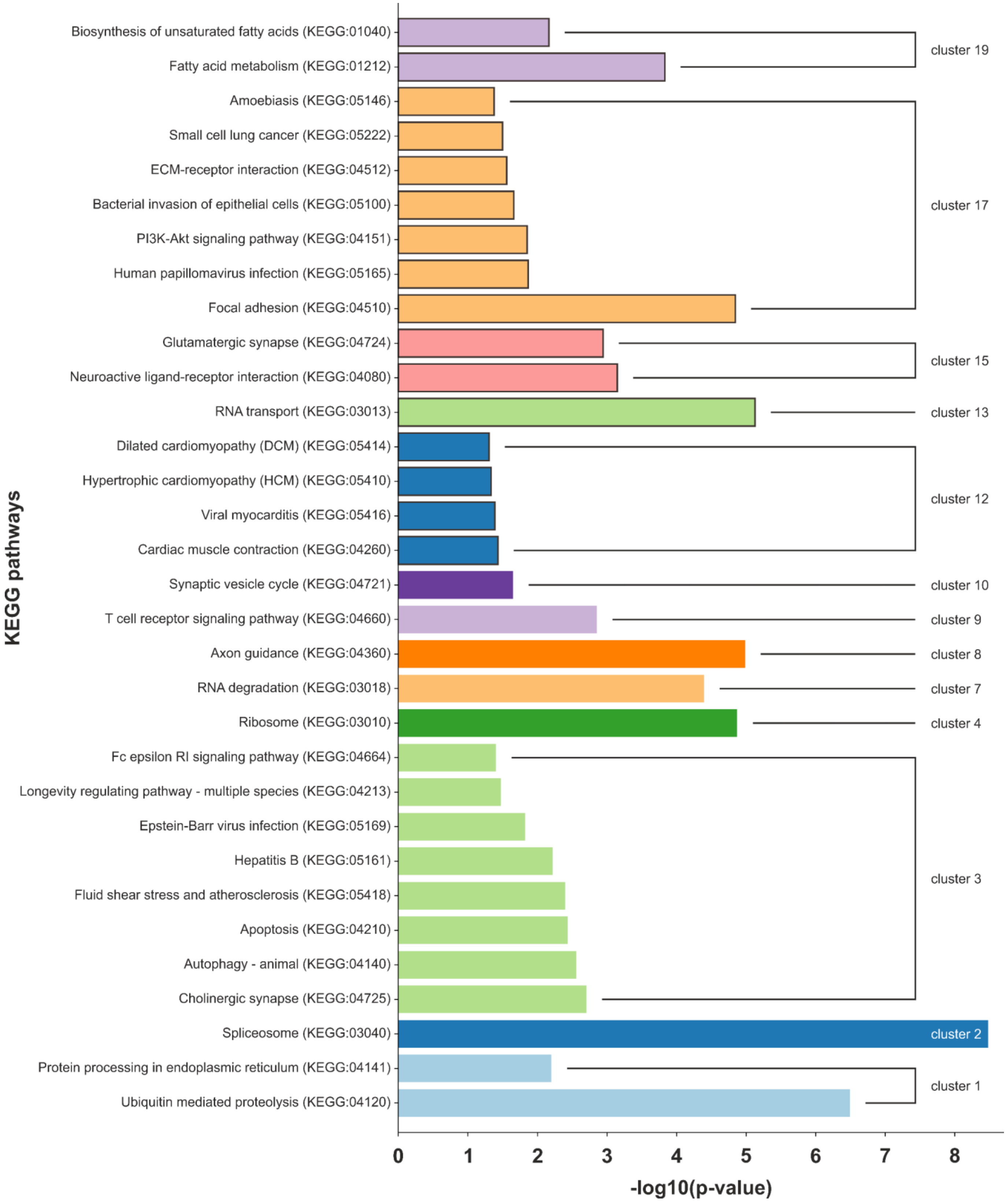

Supplementary Figure S6

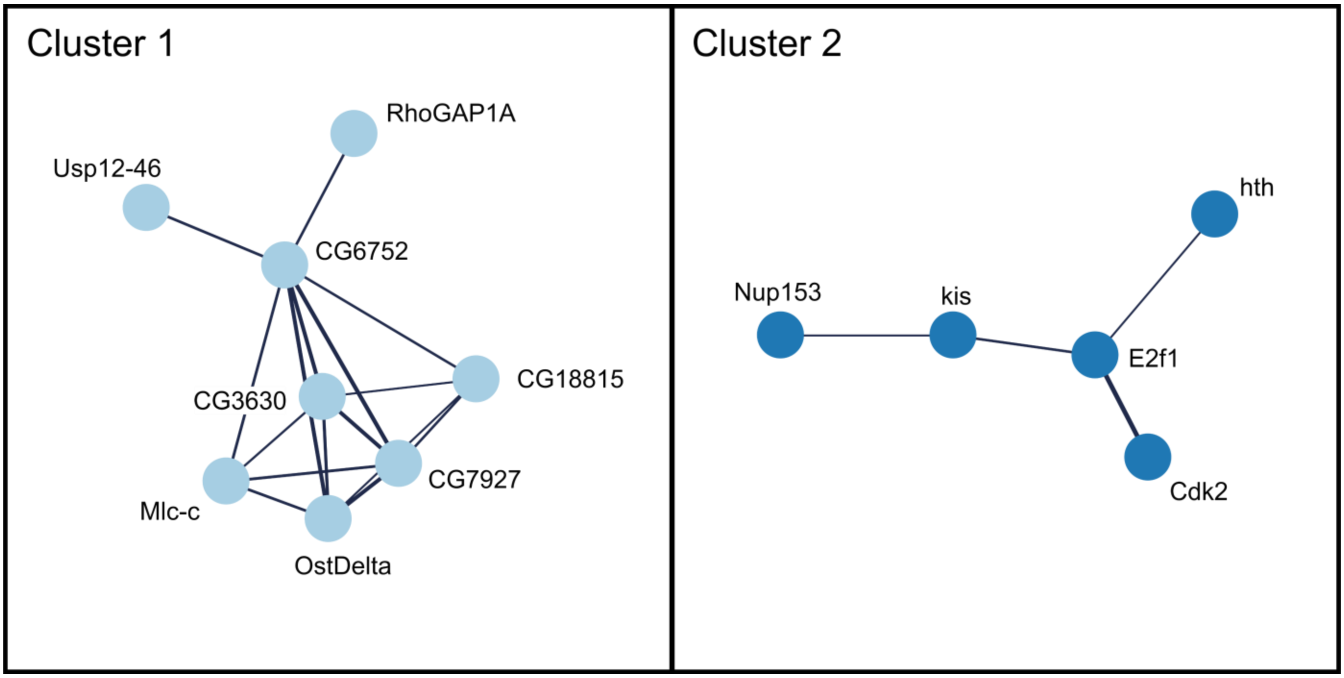

Supplementary Figure S7

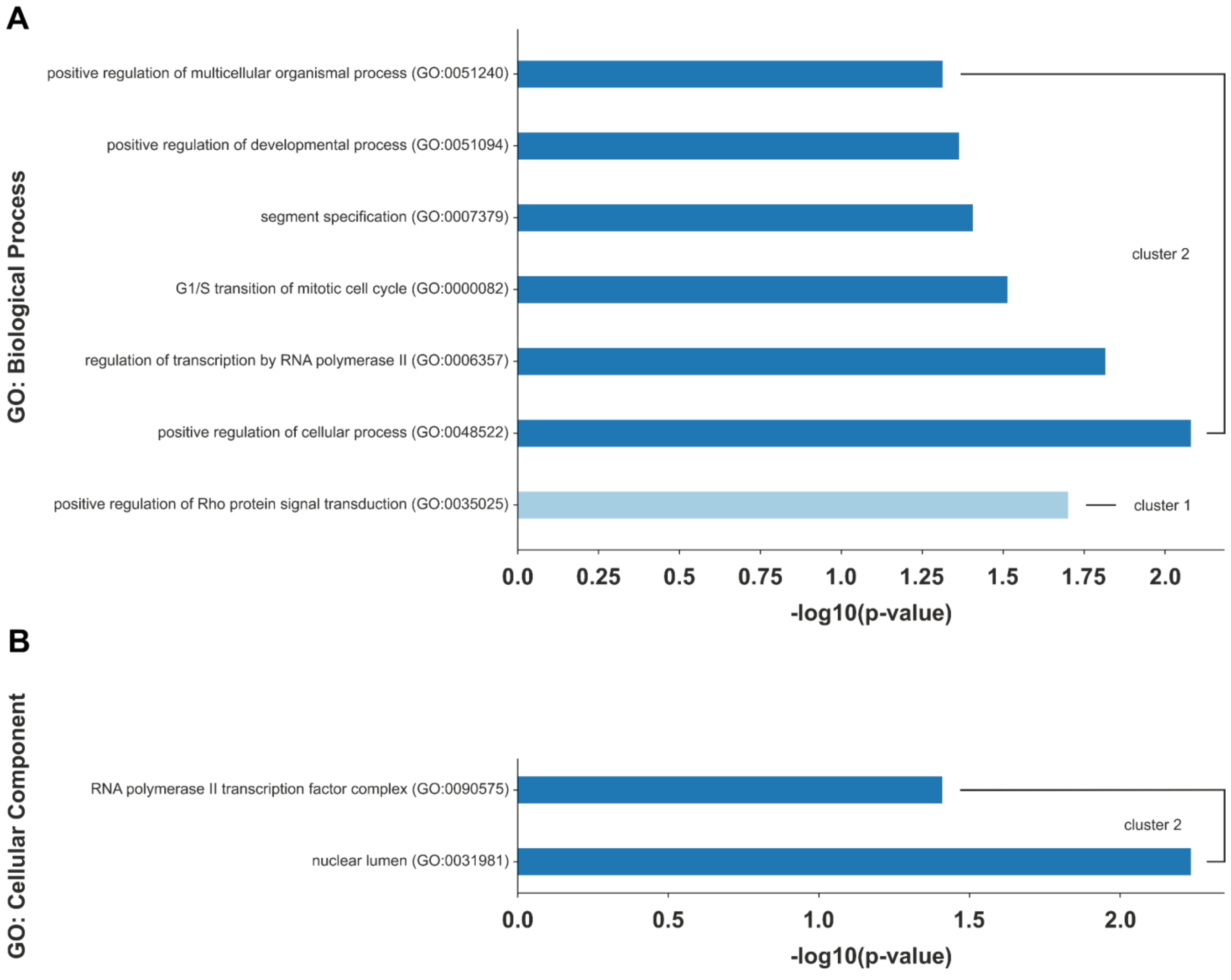

Supplementary Table S1. List of species miRNA groups and classifications from Ensembl genome catalog

Species	*Name*	*# genes*	*# mirnas*	*# intragenic*	*# antisense*	*# overlapping*	*Kingdom*	*Phylum*	*Class*	*Order*	*Family*	*Genus*	*Ensembl classification*
E. telfairi	Lesser hedgehog tenrec	25,609	470	72	28	2	Animalia	Chordata	Mammalia	Afrosoricida	Tenrecomorpha	Echinops	Afrotheria
P. capensis	Hyrax	18,273	624	88	27	0	Animalia	Chordata	Mammalia	Hyracoidea	Procaviidae	Procavia	Afrotheria
L. africana	Elephant	22,488	757	151	72	2	Animalia	Chordata	Mammalia	Proboscidea	Elephantidae	Loxodonta	Afrotheria
X. tropicalis	Xenopus	19,588	333	56	14	0	Animalia	Chordata	Amphibia	Anura	Pipidae	Xenopus	Amphibians
A. platyrhynchos	Duck	25,832	120	44	11	0	Animalia	Chordata	Aves	Anseriformes	Anatidae	Anas	Birds and Reptiles
A. brachyrhynchus	Pink-footed goose	22,530	111	37	10	1	Animalia	Chordata	Aves	Anseriformes	Anatidae	Anser	Birds and Reptiles
A. haastii	Great spotted kiwi	19,162	99	24	6	0	Animalia	Chordata	Aves	Apterygiformes	Apterygidae	Apteryx	Birds and Reptiles
A. owenii	Little spotted kiwi	18,792	102	33	10	0	Animalia	Chordata	Aves	Apterygiformes	Apterygidae	Apteryx	Birds and Reptiles
A. rowi	Okarito brown kiwi	18,830	104	38	9	0	Animalia	Chordata	Aves	Apterygiformes	Apterygidae	Apteryx	Birds and Reptiles
D. novaehollandiae	Emu	19,140	102	40	14	2	Animalia	Chordata	Aves	Casuariiformes	Casuariidae	Dromaius	Birds and Reptiles
C. pugnax	Ruff	19,928	124	49	9	1	Animalia	Chordata	Aves	Charadriiformes	Scolopacidae	Calidris	Birds and Reptiles
C. pygmaea	Spoon-billed sandpiper	18,299	123	42	7	1	Animalia	Chordata	Aves	Charadriiformes	Scolopacidae	Calidris	Birds and Reptiles
N. meleagris	Helmeted guineafowl	23,108	149	58	17	1	Animalia	Chordata	Aves	Galliformes	Numididae	Numida	Birds and Reptiles
C. japonica	Japanese quail	21,413	148	57	15	0	Animalia	Chordata	Aves	Galliformes	Phasianidae	Coturnix	Birds and Reptiles
G. gallus	Chicken	23,216	1,140	569	155	8	Animalia	Chordata	Aves	Galliformes	Phasianidae	Gallus	Birds and Reptiles
M. gallopavo	Turkey	14,572	430	130	49	0	Animalia	Chordata	Aves	Galliformes	Phasianidae	Meleagris	Birds and Reptiles
L. striata	Bengalese finch	18,090	165	56	14	2	Animalia	Chordata	Aves	Passeriformes	Estrildidae	Lonchura	Birds and Reptiles
T. guttata	Zebra Finch	18,316	302	68	37	1	Animalia	Chordata	Aves	Passeriformes	Estrildidae	Taeniopygia	Birds and Reptiles
S. canaria	Common canary	17,248	132	47	8	2	Animalia	Chordata	Aves	Passeriformes	Fringillidae	Serinus	Birds and Reptiles
F. albicollis	Flycatcher	21,398	510	146	42	2	Animalia	Chordata	Aves	Passeriformes	Muscicapidae	Ficedula	Birds and Reptiles
C. caeruleus	Blue tit	24,384	125	48	10	3	Animalia	Chordata	Aves	Passeriformes	Paridae	Cyanistes	Birds and Reptiles
P. major	Great Tit	19,874	132	63	13	1	Animalia	Chordata	Aves	Passeriformes	Paridae	Parus	Birds and Reptiles
J. hyemalis	Dark-eyed junco	17,351	126	47	16	1	Animalia	Chordata	Aves	Passeriformes	Passerellidae	Junco	Birds and Reptiles
Z. albicollis	White-throated sparrow	18,128	124	51	10	1	Animalia	Chordata	Aves	Passeriformes	Passerellidae	Zonotrichia	Birds and Reptiles
L. coronata	Blue-crowned manakin	16,410	126	33	7	1	Animalia	Chordata	Aves	Passeriformes	Pipridae	Lepidothrix	Birds and Reptiles
M. vitellinus	Golden-collared manakin	18,507	114	48	15	1	Animalia	Chordata	Aves	Passeriformes	Pipridae	Manacus	Birds and Reptiles
M. undulatus	Budgerigar	16,849	96	27	10	0	Animalia	Chordata	Aves	Psittaciformes	Psittaculidae	Melopsittacus	Birds and Reptiles
N. perdicaria	Chilean tinamou	16,175	87	23	5	0	Animalia	Chordata	Aves	Tinamiformes	Tinamidae	Nothoprocta	Birds and Reptiles
C. porosus	Australian saltwater crocodile	19,093	114	43	10	2	Animalia	Chordata	Reptilia	Crocodilia	Crocodylidae	Crocodylus	Birds and Reptiles
S. punctatus	Tuatara	19,283	51	13	4	0	Animalia	Chordata	Reptilia	Rhynchocephalia	Sphenodontidae	Sphenodon	Birds and Reptiles
P. vitticeps	Central bearded dragon	25,678	45	22	6	0	Animalia	Chordata	Reptilia	Squamata	Agamidae	Pogona	Birds and Reptiles
A. carolinensis	Anole lizard	25,497	423	101	57	0	Animalia	Chordata	Reptilia	Squamata	Dactyloidae	Anolis	Birds and Reptiles
N. scutatus	Mainland tiger snake	20,505	134	30	2	1	Animalia	Chordata	Reptilia	Squamata	Elapidae	Notechis	Birds and Reptiles
S. merianae	Argentine black and white tegu	23,653	40	18	2	1	Animalia	Chordata	Reptilia	Squamata	Teiidae	Salvator	Birds and Reptiles
C. picta	Painted turtle	26,547	270	109	26	0	Animalia	Chordata	Reptilia	Testudines	Emydidae	Chysemys	Birds and Reptiles
C. abingdonii†	Abingdon island giant tortoise	23,086	123	24	5	0	Animalia	Chordata	Reptilia	Testudines	Testudinidae	Chelonoidis	Birds and Reptiles
G. agassizii	Agassiz's desert tortoise	24,134	120	46	9	1	Animalia	Chordata	Reptilia	Testudines	Testudinidae	Gopherus	Birds and Reptiles
P. sinensis	Chinese softshell turtle	18,812	516	109	83	0	Animalia	Chordata	Reptilia	Testudines	Trionychidae	Pelodiscus	Birds and Reptiles
A. testudineus	Climbing perch	25,067	97	28	7	0	Animalia	Chordata	Actinopterygii	Anabantiformes	Anabantidae	Anabas	Fish
O. latipes HNI	Japanese medaka HNI	22,543	138	37	10	0	Animalia	Chordata	Actinopterygii	Beloniformes	Adrianichthyidae	Oryzias	Fish
O. latipes HSOK	Japanese medaka HSOK	23,189	127	28	12	0	Animalia	Chordata	Actinopterygii	Beloniformes	Adrianichthyidae	Oryzias	Fish
O. latipes HdrR	Japanese medaka HdrR	24,135	230	39	19	0	Animalia	Chordata	Actinopterygii	Beloniformes	Adrianichthyidae	Oryzias	Fish
O. melastigma	Indian medaka	23,939	80	20	10	0	Animalia	Chordata	Actinopterygii	Beloniformes	Adrianichthyidae	Oryzias	Fish
A. mexicanus	Mexican tetra	27,382	38	11	6	0	Animalia	Chordata	Actinopterygii	Characiformes	Characidae	Astyanax	Fish
P. nattereri	Red-bellied piranha	30,066	38	10	5	0	Animalia	Chordata	Actinopterygii	Characiformes	Serrasalmidae	Pygocentrus	Fish
A. citrinellus	Midas cichlid	24,348	180	41	9	0	Animalia	Chordata	Actinopterygii	Cichliformes	Cichlidae	Amphilophus	Fish
A. calliptera	Eastern happy	27,591	410	126	41	1	Animalia	Chordata	Actinopterygii	Cichliformes	Cichlidae	Astatotilapia	Fish
A. burtoni	Burton's mouthbrooder	23,490	393	136	34	1	Animalia	Chordata	Actinopterygii	Cichliformes	Cichlidae	Astatotilapia	Fish
H. nyererei	Makobe Island cichlid	23,899	373	95	26	4	Animalia	Chordata	Actinopterygii	Cichliformes	Cichlidae	Haplochromis	Fish
M. zebra	Zebra mbuna	28,203	419	124	33	1	Animalia	Chordata	Actinopterygii	Cichliformes	Cichlidae	Maylandia	Fish
N. brichardi	Lyretail cichlid	24,051	360	92	18	0	Animalia	Chordata	Actinopterygii	Cichliformes	Cichlidae	Neolamprologus	Fish
O. niloticus	Tilapia	26,692	396	101	51	1	Animalia	Chordata	Actinopterygii	Cichliformes	Cichlidae	Oreochromis	Fish
D. rerio	Zebrafish	36,807	434	137	82	5	Animalia	Chordata	Actinopterygii	Cypriniformes	Cyprinidae	Danio	Fish
K. marmoratus	Mangrove rivulus	22,236	78	22	4	0	Animalia	Chordata	Actinopterygii	Cyprinodontiformes	Aplocheilidae	Kryptolebias	Fish

C. variegatus	Sheepshead minnow	23,394	64	16	5	0	Animalia	Chordata	Actinopterygii	Cyprinodontiformes	Cyprinodontidae	Cyprinodon	Fish
F. heteroclitus	Mummichog	23,403	66	14	2	0	Animalia	Chordata	Actinopterygii	Cyprinodontiformes	Fundulidae	Fundulus	Fish
G. affinis	Western mosquitofish	21,994	63	23	5	1	Animalia	Chordata	Actinopterygii	Cyprinodontiformes	Poeciliidae	Gambusia	Fish
P. formosa	Amazon molly	23,940	414	121	43	2	Animalia	Chordata	Actinopterygii	Cyprinodontiformes	Poeciliidae	Poecilia	Fish
P. latipinna	Sailfin molly	23,959	75	16	4	0	Animalia	Chordata	Actinopterygii	Cyprinodontiformes	Poeciliidae	Poecilia	Fish
P. mexicana	Shortfin molly	24,400	75	17	4	0	Animalia	Chordata	Actinopterygii	Cyprinodontiformes	Poeciliidae	Poecilia	Fish
P. reticulata	Guppy	23,187	67	19	6	0	Animalia	Chordata	Actinopterygii	Cyprinodontiformes	Poeciliidae	Poecilia	Fish
X. couchianus	Monterrey platyfish	20,849	61	15	6	0	Animalia	Chordata	Actinopterygii	Cyprinodontiformes	Poeciliidae	Xiphophorus	Fish
X. maculatus	Platyfish	24,140	69	26	5	0	Animalia	Chordata	Actinopterygii	Cyprinodontiformes	Poeciliidae	Xiphophorus	Fish
E. lucius	Northern pike	24,567	65	21	3	0	Animalia	Chordata	Actinopterygii	Esociformes	Esocidae	Esox	Fish
G. morhua	Cod	21,740	414	60	26	1	Animalia	Chordata	Actinopterygii	Gadiformes	Gadidae	Gadus	Fish
G. aculeatus	Stickleback	21,952	504	68	32	1	Animalia	Chordata	Actinopterygii	Gasterosteiformes	Gasterosteidae	Gasterosteus	Fish
P. magnuspinnatus	Periophthalmus magnuspinnatus	24,157	40	9	4	0	Animalia	Chordata	Actinopterygii	Gobiiformes	Oxudercidae	Periophthalmus	Fish
E. electricus	Electric eel	23,366	40	18	1	4	Animalia	Chordata	Actinopterygii	Gymnotiformes	Gymnotidae	Electrophorus	Fish
L. oculatus	Spotted gar	23,058	257	77	17	0	Animalia	Chordata	Actinopterygii	Lepisosteiformes	Lepisosteidae	Lepisosteus	Fish
P. kingsleyae	Paramormyrops kingsleyae	25,669	23	7	1	0	Animalia	Chordata	Actinopterygii	Osteoglossiformes	Mormyridae	Paramormyrops	Fish
S. formosus	Asian bonytongue	24,188	31	6	4	0	Animalia	Chordata	Actinopterygii	Osteoglossiformes	Osteoglossidae	Scleropages	Fish
A. ocellaris	Clown anemonefish	24,656	117	34	6	0	Animalia	Chordata	Actinopterygii	Ovalentaria	Pomacentridae	Amphiprion	Fish
A. percula	Orange clownfish	24,717	123	36	6	0	Animalia	Chordata	Actinopterygii	Ovalentaria	Pomacentridae	Amphiprion	Fish
S. partitus	Bicolor damselfish	23,458	135	37	7	0	Animalia	Chordata	Actinopterygii	Ovalentaria	Pomacentridae	Stegastes	Fish
S. dorsalis	Yellowtail amberjack	25,425	262	62	16	0	Animalia	Chordata	Actinopterygii	Perciformes	Carangidae	Seriola	Fish
S. dumerili	Greater amberjack	23,720	125	38	15	0	Animalia	Chordata	Actinopterygii	Perciformes	Carangidae	Seriola	Fish
L. bergylta	Ballan wrasse	28,990	97	28	8	0	Animalia	Chordata	Actinopterygii	Perciformes	Labridae	Labrus	Fish
L. calcarifer	Barramundi perch	26,626	135	40	11	0	Animalia	Chordata	Actinopterygii	Perciformes	Latidae	Lates	Fish
C. semilaevis	Tongue sole	21,747	64	16	4	0	Animalia	Chordata	Actinopterygii	Pleuronectiformes	Cynoglossidae	Cynoglossus	Fish
S. maximus	Turbot	21,365	82	36	9	0	Animalia	Chordata	Actinopterygii	Pleuronectiformes	Scophthalmidae	Scophthalmus	Fish
A. polyacanthus	Spiny chromis	24,439	126	36	7	0	Animalia	Chordata	Actinopterygii	Pomacentridae	Chrominae	Acanthochromis	Fish
H. hucho	Huchen	52,651	289	70	14	0	Animalia	Chordata	Actinopterygii	Salmoniformes	Salmonidae	Hucho	Fish
I. punctatus	Channel catfish	25,062	56	18	9	0	Animalia	Chordata	Actinopterygii	Siluriformes	Ictaluridae	Ictalurus	Fish
M. armatus	Zig-zag eel	24,152	105	28	8	1	Animalia	Chordata	Actinopterygii	Synbranchiformes	Mastacembelidae	Mastacembelus	Fish
M. albus	Swamp eel	22,624	84	26	5	0	Animalia	Chordata	Actinopterygii	Synbranchiformes	Synbranchidae	Monopterus	Fish
H. comes	Tiger tail seahorse	21,179	47	16	2	1	Animalia	Chordata	Actinopterygii	Syngnathiformes	Syngnathidae	Hippocampus	Fish
M. mola	Ocean sunfish	21,815	78	19	6	0	Animalia	Chordata	Actinopterygii	Tetraodontiformes	Molidae	Mola	Fish
T. rubripes	Fugu	20,956	153	33	11	0	Animalia	Chordata	Actinopterygii	Tetraodontiformes	Tetraodontidae	Takifugu	Fish
T. nigroviridis	Tetraodon	20,165	397	58	25	0	Animalia	Chordata	Actinopterygii	Tetraodontiformes	Tetraodontidae	Tetraodon	Fish
B. bison	American bison	24,826	683	226	54	15	Animalia	Chordata	Mammalia	Artiodactyla	Bovidae	Bison	Laurasiatheria
B. mutus	Wild yak	25,103	669	287	70	19	Animalia	Chordata	Mammalia	Artiodactyla	Bovidae	Bos	Laurasiatheria
B. taurus	Cow	26,656	951	454	107	22	Animalia	Chordata	Mammalia	Artiodactyla	Bovidae	Bos	Laurasiatheria
C. aegagrus hircus	Goat	26,957	314	104	33	3	Animalia	Chordata	Mammalia	Artiodactyla	Bovidae	Capra	Laurasiatheria
O. aries	Sheep	25,749	1,305	369	216	2	Animalia	Chordata	Mammalia	Artiodactyla	Bovidae	Ovis	Laurasiatheria
V. pacos	Alpaca	14,593	602	68	41	2	Animalia	Chordata	Mammalia	Artiodactyla	Camelidae	Vicugna	Laurasiatheria
T. truncatus	Dolphin	20,164	1,101	229	111	0	Animalia	Chordata	Mammalia	Artiodactyla	Delphinidae	Tursiops	Laurasiatheria
S. scrofa swine	Pig	25,326	554	202	105	9	Animalia	Chordata	Mammalia	Artiodactyla	Suidae	Sus	Laurasiatheria
S. scrofa	Pig USMARC	27,833	489	167	54	7	Animalia	Chordata	Mammalia	Artiodactyla	Suidae	Sus	Laurasiatheria
C. lupus dingo	Dingo	26,064	493	127	40	3	Animalia	Chordata	Mammalia	Carnivora	Canidae	Canis	Laurasiatheria
C. lupus familiaris	Dog	31,858	846	278	162	1	Animalia	Chordata	Mammalia	Carnivora	Canidae	Canis	Laurasiatheria
V. vulpes	Red fox	28,942	316	121	39	4	Animalia	Chordata	Mammalia	Carnivora	Canidae	Vulpes	Laurasiatheria
F. catus	Cat	26,336	345	125	32	4	Animalia	Chordata	Mammalia	Carnivora	Felidae	Felis	Laurasiatheria
P. tigris	Tiger	21,825	294	96	25	1	Animalia	Chordata	Mammalia	Carnivora	Felidae	Panthera	Laurasiatheria
P. pardus	Leopard	24,728	365	111	37	2	Animalia	Chordata	Mammalia	Carnivora	Felidae	Panthera	Laurasiatheria
M. putorius	Ferret	31,239	818	259	126	5	Animalia	Chordata	Mammalia	Carnivora	Mustelidae	Mustela	Laurasiatheria
N. vison	American mink	24,322	204	73	15	1	Animalia	Chordata	Mammalia	Carnivora	Mustelidae	Neovison	Laurasiatheria
A. melanoleuca	Panda	22,519	743	154	105	1	Animalia	Chordata	Mammalia	Carnivora	Ursidae	Ailuropoda	Laurasiatheria
U. americanus	American black bear	24,423	224	53	17	2	Animalia	Chordata	Mammalia	Carnivora	Ursidae	Ursus	Laurasiatheria
U. maritimus	Polar bear	24,140	205	72	13	0	Animalia	Chordata	Mammalia	Carnivora	Ursidae	Ursus	Laurasiatheria
P. vampyrus	Megabat	21,348	846	126	78	1	Animalia	Chordata	Mammalia	Chiroptera	Pteropodidae	Pteropus	Laurasiatheria
M. lucifugus	Microbat	25,064	785	148	81	2	Animalia	Chordata	Mammalia	Chiroptera	Vespertilionidae	Myotis	Laurasiatheria

E. europaeus	Hedgehog	21,922	526	68	36	1	Animalia	Chordata	Mammalia	Eulipotyphla	Erinaceidae	Erinaceus	Laurasiatheria
S. araneus	Shrew	18,627	507	54	28	1	Animalia	Chordata	Mammalia	Eulipotyphla	Soricidae	Sorex	Laurasiatheria
E. africanus	Donkey	22,529	399	116	38	4	Animalia	Chordata	Mammalia	Perissodactyla	Equidae	Equus	Laurasiatheria
E. ferus caballus	Horse	30,548	668	246	83	11	Animalia	Chordata	Mammalia	Perissodactyla	Equidae	Equus	Laurasiatheria
C. intestinalis	C.intestinalis	16,861	292	95	43	1	Animalia	Chordata	Ascidiacea	Enterogona	Cionidae	Ciona	Other chordates
C. savignyi	C.savignyi	12,110	62	11	2	0	Animalia	Chordata	Ascidiacea	Enterogona	Cionidae	Ciona	Other chordates
D. melanogaster	Drosophila melanogaster	17,494	259	184	36	3	Animalia	Arthropoda	Insecta	Diptera	Drosophilidae	Drosophila	Other eukaryotes
C. elegans	Caenorhabditis elegans	46,644	260	84	43	16	Animalia	Nematoda	Chromadorea	Rhabditida	Rhabditidae	Caenorhabditis	Other eukaryotes
S. harrisii	Tasmanian devil	19,970	486	98	33	1	Animalia	Chordata	Mammalia	Dasyuromorphia	Dasyuridae	Sarcophilus	Other mammals
M. domestica	Opossum	29,957	412	112	79	2	Animalia	Chordata	Mammalia	Didelphimorphia	Didelphidae	Monodelphis	Other mammals
M. eugenii	Wallaby	17,733	525	36	16	1	Animalia	Chordata	Mammalia	Diprotodontia	Macropodidae	Macropus	Other mammals
P. cinereus	Koala	25,349	128	55	11	1	Animalia	Chordata	Mammalia	Diprotodontia	Phascolarctidae	Phascolarctos	Other mammals
V. ursinus	Common wombat	24,553	543	162	57	7	Animalia	Chordata	Mammalia	Diprotodontia	Vombatidae	Vombatus	Other mammals
O. anatinus	Platypus	28,953	823	91	60	1	Animalia	Chordata	Mammalia	Monotremata	Ornithorhynchidae	Ornithorhynchus	Other mammals
C. milii	Elephant shark	20,080	4	2	0	0	Animalia	Chordata	Chondrichthyes	Chimaeriformes	Callorhinchidae	Callorhinchus	Other vertebrates
P. marinus	Lamprey	12,784	330	44	14	2	Animalia	Chordata	Hyperoartia	Petromyzontiformes	Petromyzontidae	Petromyzon	Other vertebrates
E. burgeri	Hagfish	17,217	29	10	5	0	Animalia	Chordata	Myxini	Myxiniformes	Myxinidae	Eptatretus	Other vertebrates
L. chalumnae	Coelacanth	22,113	515	107	53	1	Animalia	Chordata	Sarcopterygii	Coelacanthiformes	Latimeriidae	Latimeria	Other vertebrates
A. nancymaae	Ma's night monkey	28,065	897	301	102	10	Animalia	Chordata	Mammalia	Primates	Aotidae	Aotus	Primates
C. jacchus	Marmoset	28,459	827	269	103	8	Animalia	Chordata	Mammalia	Primates	Callitrichidae	Callithrix	Primates
C. capucinus	Capuchin	27,104	884	293	121	11	Animalia	Chordata	Mammalia	Primates	Cebidae	Cebus	Primates
S. boliviensis	Bolivian squirrel monkey	26,708	794	267	104	10	Animalia	Chordata	Mammalia	Primates	Cebidae	Saimiri	Primates
C. atys	Sooty mangabey	27,147	1,312	534	152	8	Animalia	Chordata	Mammalia	Primates	Cercopithecidae	Cercocebus	Primates
C. sabaeus	Vervet-AGM	24,849	3,136	900	337	17	Animalia	Chordata	Mammalia	Primates	Cercopithecidae	Chlorocebus	Primates
C. angolensis	Angola colobus	25,939	1,313	470	141	7	Animalia	Chordata	Mammalia	Primates	Cercopithecidae	Colobus	Primates
M. fascicularis	Crab-eating macaque	27,990	1,334	543	147	12	Animalia	Chordata	Mammalia	Primates	Cercopithecidae	Macaca	Primates
M. mulatta	Macaque	30,047	2,339	732	219	17	Animalia	Chordata	Mammalia	Primates	Cercopithecidae	Macaca	Primates
M. nemestrina	Pig-tailed macaque	27,559	1,306	516	153	11	Animalia	Chordata	Mammalia	Primates	Cercopithecidae	Macaca	Primates
M. leucophaeus	Drill	26,446	1,257	446	121	8	Animalia	Chordata	Mammalia	Primates	Cercopithecidae	Mandrillus	Primates
P. anubis	Olive baboon	28,097	1,379	482	136	13	Animalia	Chordata	Mammalia	Primates	Cercopithecidae	Papio	Primates
P. tephrosceles	Ugandan red Colobus	37,249	394	139	35	4	Animalia	Chordata	Mammalia	Primates	Cercopithecidae	Piliocolobus	Primates
P. bieti	Black snub-nosed monkey	28,591	1,314	454	137	14	Animalia	Chordata	Mammalia	Primates	Cercopithecidae	Rhinopithecus	Primates
R. roxellana	Golden snub-nosed monkey	27,516	1,367	484	137	15	Animalia	Chordata	Mammalia	Primates	Cercopithecidae	Rhinopithecus	Primates
T. gelada	Gelada	28,876	495	195	39	3	Animalia	Chordata	Mammalia	Primates	Cercopithecidae	Theropithecus	Primates
M. murinus	Mouse Lemur	26,375	423	162	43	3	Animalia	Chordata	Mammalia	Primates	Cheirogaleidae	Microcebus	Primates
O. garnettii	Bushbaby	27,237	848	187	88	4	Animalia	Chordata	Mammalia	Primates	Galagidae	Otolemur	Primates
G. gorilla	Gorilla	28,038	2,046	813	206	8	Animalia	Chordata	Mammalia	Primates	Hominidae	Gorilla	Primates
H. sapiens	Human	64,921	1,911	1294	389	15	Animalia	Chordata	Mammalia	Primates	Hominidae	Homo	Primates
P. paniscus	Bonobo	28,310	1,948	783	184	10	Animalia	Chordata	Mammalia	Primates	Hominidae	Pan	Primates
P. troglodytes	Chimpanzee	31,488	2,241	902	240	17	Animalia	Chordata	Mammalia	Primates	Hominidae	Pan	Primates
P. abelii	Orangutan	26,821	1,622	395	164	3	Animalia	Chordata	Mammalia	Primates	Hominidae	Pongo	Primates
N. leucogenys	Gibbon	26,334	1,492	543	148	5	Animalia	Chordata	Mammalia	Primates	Hylobatidae	Nomascus	Primates
P. coquereli	Coquerel's sifaka	23,208	427	135	39	6	Animalia	Chordata	Mammalia	Primates	Indriidae	Propithecus	Primates
P. simus	Greater bamboo lemur	25,867	232	67	16	0	Animalia	Chordata	Mammalia	Primates	Lemuridae	Prolemur	Primates
C. syrichta	Tarsier	24,536	385	91	30	7	Animalia	Chordata	Mammalia	Primates	Tarsiidae	Carlito	Primates
O. cuniculus	Rabbit	23,081	588	159	80	1	Animalia	Chordata	Mammalia	Lagomorpha	Leporidae	Oryctolagus	Rodents
O. princeps	Pika	22,292	773	77	41	0	Animalia	Chordata	Mammalia	Lagomorpha	Ochotonidae	Ochotona	Rodents
F. damarensis	Damara mole rat	33,153	409	119	38	3	Animalia	Chordata	Mammalia	Rodentia	Bathyergidae	Fukomys	Rodents
C. canadensis	American beaver	28,773	157	57	9	1	Animalia	Chordata	Mammalia	Rodentia	Castoridae	Castor	Rodents
C. aperea	Brazilian guinea pig	17,682	348	66	21	3	Animalia	Chordata	Mammalia	Rodentia	Caviidae	Cavia	Rodents
C. porcellus	Guinea Pig	26,381	474	145	48	4	Animalia	Chordata	Mammalia	Rodentia	Caviidae	Cavia	Rodents
C. lanigera	Long-tailed chinchilla	28,799	462	157	56	4	Animalia	Chordata	Mammalia	Rodentia	Chinchillidae	Chinchilla	Rodents
C. griseus ovary	Chinese hamster CHOK1GS	24,224	848	219	76	14	Animalia	Chordata	Mammalia	Rodentia	Cricetidae	Cricetulus	Rodents
C. griseus	Chinese hamster CriGri	25,926	742	176	58	14	Animalia	Chordata	Mammalia	Rodentia	Cricetidae	Cricetulus	Rodents
C. griseus scaffold	Chinese hamster PICR	28,189	283	107	21	8	Animalia	Chordata	Mammalia	Rodentia	Cricetidae	Cricetulus	Rodents
M. auratus	Golden Hamster	21,511	772	193	67	11	Animalia	Chordata	Mammalia	Rodentia	Cricetidae	Mesocricetus	Rodents
M. ochrogaster	Prairie vole	22,267	771	252	71	15	Animalia	Chordata	Mammalia	Rodentia	Cricetidae	Microtus	Rodents

P. maniculatus	Northern American deer mouse	25,220	174	54	15	0	Animalia	Chordata	Mammalia	Rodentia	Cricetidae	Peromyscus	Rodents
J. jaculus	Lesser Egyptian jerboa	24,124	309	80	37	6	Animalia	Chordata	Mammalia	Rodentia	Dipodidae	Jaculus	Rodents
H. glaber female	Naked mole-rat female	31,322	484	169	62	7	Animalia	Chordata	Mammalia	Rodentia	Heterocephalidae	Heterocephalus	Rodents
H. glaber male	Naked mole-rat male	32,295	452	145	41	5	Animalia	Chordata	Mammalia	Rodentia	Heterocephalidae	Heterocephalus	Rodents
D. ordii	Kangaroo rat	20,140	402	113	34	5	Animalia	Chordata	Mammalia	Rodentia	Heteromyidae	Dipodomys	Rodents
M. unguiculatus	Mongolian gerbil	25,576	169	42	9	1	Animalia	Chordata	Mammalia	Rodentia	Muridae	Meriones	Rodents
M. caroli	Ryukyu mouse	35,291	1,491	855	313	47	Animalia	Chordata	Mammalia	Rodentia	Muridae	Mus	Rodents
M. musculus	Mouse	54,128	2,265	1204	447	67	Animalia	Chordata	Mammalia	Rodentia	Muridae	Mus	Rodents
M. pahari	Shrew mouse	33,703	1,385	817	259	47	Animalia	Chordata	Mammalia	Rodentia	Muridae	Mus	Rodents
M. spicilegus	Steppe mouse	30,274	598	292	57	14	Animalia	Chordata	Mammalia	Rodentia	Muridae	Mus	Rodents
M. spretus	Algerian mouse	36,943	1,614	885	288	47	Animalia	Chordata	Mammalia	Rodentia	Muridae	Mus	Rodents
R. norvegicus	Rat	31,295	1,588	489	216	44	Animalia	Chordata	Mammalia	Rodentia	Muridae	Rattus	Rodents
O. degus	Degu	25,438	465	123	42	5	Animalia	Chordata	Mammalia	Rodentia	Octodontidae	Octodon	Rodents
I. tridecemlineatus	Squirrel	24,742	459	161	53	4	Animalia	Chordata	Mammalia	Rodentia	Sciuridae	Ictidomys	Rodents
M. marmota	Alpine marmot	23,856	168	46	11	0	Animalia	Chordata	Mammalia	Rodentia	Sciuridae	Marmota	Rodents
S. dauricus	Daurian ground squirrel	24,820	175	47	8	0	Animalia	Chordata	Mammalia	Rodentia	Sciuridae	Spermophilus	Rodents
U. parryii	Arctic ground squirrel	23,340	175	69	13	1	Animalia	Chordata	Mammalia	Rodentia	Sciuridae	Urocitellus	Rodents
S. galili	Upper Galilee mountains blind mole rat	24,054	582	130	40	10	Animalia	Chordata	Mammalia	Rodentia	Spalacidae	Spalax	Rodents
T. belangeri	Tree Shrew	20,217	640	74	45	0	Animalia	Chordata	Mammalia	Scandentia	Tupaiidae	Tupaia	Rodents
D. novemcinctus	Armadillo	32,533	841	203	108	2	Animalia	Chordata	Mammalia	Cingulata	Dasypodidae	Dasypus	Xenarthra
C. hoffmanni	Sloth	15,510	550	47	14	1	Animalia	Chordata	Mammalia	Pilosa	Choloepodidae	Choloepus	Xenarthra

Supplementary Table S2. Network statistics for protein-protein interaction enrichments

	HSA	*MMU*	*DME*
number of nodes	909	595	126
number of edges	4078	1456	71
expected number of edges	3647	1307	46
average node degree	8.97	4.89	1.13
average local clustering coefficient	0.3	0.333	0.318
p-value	1.23E-12	2.68E-05	4.26E-04

Supplementary Table S3. Essential vs. non-essential genes for the different miRNA types and species

	species	*essential*	*non-essential*
intragenic	*HSA*	444	493
	MMU	283	391
	DME	8	119
antisense	*HSA*	81	133
	MMU	30	91
	DME	2	20
overlappin	*HSA*	3	3
	MMU	1	3
	DME	0	3

Supplementary Table S4. Indirect autoregulation of host gene related protein-protein networks by HSA intragenic miRNAs

community	*# validated miRNAs*	*gene number*	*miRNA number*	*ratio genes*	*ratio miRNAs*	*interaction ratio*
28	34	60	18	0.57	1.89	3.15
11	216	264	36	0.82	6.00	2.27
35	56	318	9	0.18	6.22	1.96
8	5745	1172	264	4.90	21.76	1.86
4	2585	994	141	2.60	18.33	1.84
7	192	209	51	0.92	3.76	1.80
13	573	398	81	1.44	7.07	1.78
6	1857	779	150	2.38	12.38	1.59
12	567	484	78	1.17	7.27	1.50
16	482	395	99	1.22	4.87	1.23
3	532	531	93	1.00	5.72	1.08
0	879	575	150	1.53	5.86	1.02
5	3939	1334	300	2.95	13.13	0.98
19	190	340	57	0.56	3.33	0.98
15	78	182	45	0.43	1.73	0.95
30	3	53	6	0.06	0.50	0.94
10	757	644	126	1.18	6.01	0.93
23	32	124	30	0.26	1.07	0.86
14	483	542	105	0.89	4.60	0.85
18	76	345	30	0.22	2.53	0.73
9	367	560	96	0.66	3.82	0.68
1	28	146	30	0.19	0.93	0.64
2	22	185	27	0.12	0.81	0.44
20	11	108	27	0.10	0.41	0.38
22	0	72	6	0.00	0.00	0.00
33	0	49	6	0.00	0.00	0.00

Supplementary Table S5. Indirect autoregulation of host gene related protein-protein networks by MMU intragenic miRNAs

community	*# validated miRNAs*	*gene number*	*miRNA number*	*ratio genes*	*ratio miRNAs*	*interaction ratio*
66	8	20	12	0.40	0.67	3.33
1	11	79	6	0.14	1.83	2.32
33	2	10	9	0.20	0.22	2.22
28	35	98	18	0.36	1.94	1.98
5	298	327	51	0.91	5.84	1.79
6	1540	868	117	1.77	13.16	1.52
3	275	381	51	0.72	5.39	1.42
13	830	746	93	1.11	8.92	1.20
12	191	427	39	0.45	4.90	1.15
7	269	464	54	0.58	4.98	1.07
0	163	356	51	0.46	3.20	0.90
8	1513	1064	186	1.42	8.13	0.76
23	13	121	18	0.11	0.72	0.60
27	3	91	6	0.03	0.50	0.55
10	137	522	48	0.26	2.85	0.55
17	7	86	15	0.08	0.47	0.54
22	20	218	18	0.09	1.11	0.51
20	14	161	21	0.09	0.67	0.41
16	138	453	75	0.30	1.84	0.41
15	27	229	30	0.12	0.90	0.39
30	13	149	24	0.09	0.54	0.36
11	15	254	21	0.06	0.71	0.28
9	6	182	12	0.03	0.50	0.27
14	85	495	63	0.17	1.35	0.27
4	62	487	51	0.13	1.22	0.25
19	15	444	21	0.03	0.71	0.16
21	0	60	6	0.00	0.00	0.00
29	0	51	6	0.00	0.00	0.00

Supplementary Table S6. Enrichment Table for each separate community in HSA, ranked by the p-value

GO-Terms	*description*	*source*	*p-value*	*community*
KEGG:00564	Glycerophospholipid metabolism	KEGG	8.75376E-50	0
GO:0008076	voltage-gated potassium channel complex	GO:CC	1.8449E-37	0
KEGG:04140	Autophagy - animal	KEGG	9.57788E-35	0
KEGG:00600	Sphingolipid metabolism	KEGG	2.43173E-34	0
GO:0005251	delayed rectifier potassium channel activity	GO:MF	4.00956E-31	0
KEGG:04150	mTOR signaling pathway	KEGG	1.51866E-27	0
KEGG:04136	Autophagy - other	KEGG	4.92177E-27	0
GO:0000421	autophagosome membrane	GO:CC	1.38055E-19	0
GO:0046513	ceramide biosynthetic process	GO:BP	2.44482E-19	0
GO:0005789	endoplasmic reticulum membrane	GO:CC	1.19851E-18	0
GO:0000422	autophagy of mitochondrion	GO:BP	7.58406E-18	0
GO:0005765	lysosomal membrane	GO:CC	5.02655E-17	0
GO:0061952	midbody abscission	GO:BP	6.32525E-16	0
GO:0051260	protein homooligomerization	GO:BP	3.525E-14	0
GO:0034045	phagophore assembly site membrane	GO:CC	1.2706E-13	0
GO:0031932	TORC2 complex	GO:CC	1.61458E-13	0
KEGG:04211	Longevity regulating pathway	KEGG	2.35839E-13	0
KEGG:04152	AMPK signaling pathway	KEGG	1.90445E-12	0
KEGG:00565	Ether lipid metabolism	KEGG	3.88153E-12	0
GO:0000813	ESCRT I complex	GO:CC	6.42762E-12	0
GO:0016024	CDP-diacylglycerol biosynthetic process	GO:BP	6.60146E-12	0
GO:1902188	positive regulation of viral release from host cell	GO:BP	2.80176E-11	0
KEGG:04810	Regulation of actin cytoskeleton	KEGG	3.14796E-89	1
KEGG:04360	Axon guidance	KEGG	8.15413E-82	1
KEGG:04015	Rap1 signaling pathway	KEGG	6.94719E-70	1
KEGG:04014	Ras signaling pathway	KEGG	1.37513E-67	1
KEGG:04510	Focal adhesion	KEGG	4.31344E-65	1
KEGG:04151	PI3K-Akt signaling pathway	KEGG	6.36676E-61	1
KEGG:04630	JAK-STAT signaling pathway	KEGG	1.60698E-52	1
KEGG:04010	MAPK signaling pathway	KEGG	2.7643E-52	1
GO:0008360	regulation of cell shape	GO:BP	2.99581E-49	1
GO:0005925	focal adhesion	GO:CC	6.52815E-49	1
KEGG:00562	Inositol phosphate metabolism	KEGG	6.95334E-44	1
KEGG:04012	ErbB signaling pathway	KEGG	2.82346E-40	1
GO:0005096	GTPase activator activity	GO:MF	1.18424E-37	1
KEGG:01521	EGFR tyrosine kinase inhibitor resistance	KEGG	1.82731E-37	1
GO:0098978	glutamatergic synapse	GO:CC	6.37348E-36	1
GO:0005829	cytosol	GO:CC	7.02894E-36	1
KEGG:05200	Pathways in cancer	KEGG	7.55941E-36	1
KEGG:04070	Phosphatidylinositol signaling system	KEGG	1.28306E-35	1
GO:0007229	integrin-mediated signaling pathway	GO:BP	4.37133E-34	1
KEGG:04722	Neurotrophin signaling pathway	KEGG	5.08711E-33	1
GO:0048013	ephrin receptor signaling pathway	GO:BP	1.3818E-32	1
GO:0051015	actin filament binding	GO:MF	4.27817E-32	1
KEGG:05205	Proteoglycans in cancer	KEGG	8.82683E-30	1
GO:0017124	SH3 domain binding	GO:MF	1.32455E-29	1
KEGG:04664	Fc epsilon RI signaling pathway	KEGG	2.49902E-28	1
KEGG:04666	Fc gamma R-mediated phagocytosis	KEGG	8.06999E-28	1
GO:0005524	ATP binding	GO:MF	2.08157E-27	1
GO:0005516	calmodulin binding	GO:MF	1.25433E-26	1
KEGG:05135	Yersinia infection	KEGG	1.07849E-25	1
KEGG:05218	Melanoma	KEGG	1.33566E-25	1
GO:0008083	growth factor activity	GO:MF	2.04396E-24	1
KEGG:04660	T cell receptor signaling pathway	KEGG	3.09459E-24	1

GO:0030426	growth cone	GO:CC	7.44507E-24	1
KEGG:05206	MicroRNAs in cancer	KEGG	3.10743E-23	1
GO:0001784	phosphotyrosine residue binding	GO:MF	5.76921E-23	1
GO:0004715	non-membrane spanning protein tyrosine kinase activity	GO:MF	1.69593E-22	1
GO:0046875	ephrin receptor binding	GO:MF	2.84979E-22	1
GO:0035335	peptidyl-tyrosine dephosphorylation	GO:BP	6.41786E-22	1
GO:0004725	protein tyrosine phosphatase activity	GO:MF	1.59286E-21	1
GO:0048471	perinuclear region of cytoplasm	GO:CC	2.05003E-21	1
KEGG:05414	Dilated cardiomyopathy (DCM)	KEGG	6.19462E-21	1
KEGG:05410	Hypertrophic cardiomyopathy (HCM)	KEGG	8.14543E-21	1
GO:0045499	chemorepellent activity	GO:MF	1.73632E-20	1
GO:0042531	positive regulation of tyrosine phosphorylation of STAT protein	GO:BP	2.28832E-20	1
KEGG:05223	Non-small cell lung cancer	KEGG	3.01329E-20	1
KEGG:04650	Natural killer cell mediated cytotoxicity	KEGG	9.59996E-20	1
KEGG:05214	Glioma	KEGG	1.22918E-19	1
KEGG:05100	Bacterial invasion of epithelial cells	KEGG	1.22918E-19	1
GO:0030175	filopodium	GO:CC	2.32013E-19	1
KEGG:04662	B cell receptor signaling pathway	KEGG	4.27711E-19	1
GO:0046854	phosphatidylinositol phosphorylation	GO:BP	8.13415E-19	1
KEGG:04060	Cytokine-cytokine receptor interaction	KEGG	8.36218E-19	1
KEGG:04670	Leukocyte transendothelial migration	KEGG	1.18752E-18	1
GO:0005070	SH3/SH2 adaptor activity	GO:MF	1.32941E-18	1
GO:0001725	stress fiber	GO:CC	1.49678E-18	1
GO:0038083	peptidyl-tyrosine autophosphorylation	GO:BP	2.94607E-18	1
KEGG:04370	VEGF signaling pathway	KEGG	1.31337E-17	1
GO:0070374	positive regulation of ERK1 and ERK2 cascade	GO:BP	4.12315E-17	1
GO:0005178	integrin binding	GO:MF	5.13283E-17	1
KEGG:04611	Platelet activation	KEGG	5.70057E-17	1
GO:0004198	calcium-dependent cysteine-type endopeptidase activity	GO:MF	9.86793E-17	1
GO:0051496	positive regulation of stress fiber assembly	GO:BP	1.24473E-16	1
KEGG:04072	Phospholipase D signaling pathway	KEGG	1.31926E-16	1
GO:0008305	integrin complex	GO:CC	1.58593E-16	1
KEGG:04062	Chemokine signaling pathway	KEGG	1.93945E-16	1
GO:0008543	fibroblast growth factor receptor signaling pathway	GO:BP	3.96103E-16	1
KEGG:05210	Colorectal cancer	KEGG	9.68681E-16	1
KEGG:05231	Choline metabolism in cancer	KEGG	1.24723E-15	1
KEGG:04530	Tight junction	KEGG	1.52897E-15	1
KEGG:04910	Insulin signaling pathway	KEGG	3.6349E-15	1
KEGG:05211	Renal cell carcinoma	KEGG	4.11243E-15	1
GO:0043197	dendritic spine	GO:CC	1.26156E-14	1
GO:0032587	ruffle membrane	GO:CC	2.14276E-14	1
GO:0042169	SH2 domain binding	GO:MF	3.18712E-14	1
KEGG:05412	Arrhythmogenic right ventricular cardiomyopathy (ARVC)	KEGG	3.37436E-14	1
GO:0045296	cadherin binding	GO:MF	3.50358E-14	1
KEGG:05213	Endometrial cancer	KEGG	4.33552E-14	1
KEGG:05220	Chronic myeloid leukemia	KEGG	5.9352E-14	1
KEGG:04933	AGE-RAGE signaling pathway in diabetic complications	KEGG	9.07479E-14	1
GO:0042803	protein homodimerization activity	GO:MF	1.25564E-13	1
GO:0030215	semaphorin receptor binding	GO:MF	1.26185E-13	1
KEGG:05212	Pancreatic cancer	KEGG	1.50851E-13	1
GO:0048843	negative regulation of axon extension involved in axon guidance	GO:BP	1.60858E-13	1
KEGG:04917	Prolactin signaling pathway	KEGG	2.18059E-13	1
GO:0009897	external side of plasma membrane	GO:CC	2.79771E-13	1
GO:0014068	positive regulation of phosphatidylinositol 3-kinase signaling	GO:BP	3.29346E-13	1

GO:0008285	negative regulation of cell population proliferation	GO:BP	3.50155E-13	1
KEGG:04640	Hematopoietic cell lineage	KEGG	3.61431E-13	1
KEGG:04210	Apoptosis	KEGG	9.73427E-13	1
KEGG:04659	Th17 cell differentiation	KEGG	1.28413E-12	1
KEGG:05226	Gastric cancer	KEGG	1.30842E-12	1
GO:0030018	Z disc	GO:CC	1.5357E-12	1
GO:0030165	PDZ domain binding	GO:MF	2.04083E-12	1
KEGG:05215	Prostate cancer	KEGG	3.3752E-12	1
KEGG:01522	Endocrine resistance	KEGG	3.52085E-12	1
GO:0000187	activation of MAPK activity	GO:BP	3.67304E-12	1
KEGG:05221	Acute myeloid leukemia	KEGG	4.27916E-12	1
GO:0035235	ionotropic glutamate receptor signaling pathway	GO:BP	4.60762E-12	1
GO:0017154	semaphorin receptor activity	GO:MF	4.73207E-12	1
KEGG:04625	C-type lectin receptor signaling pathway	KEGG	5.79701E-12	1
GO:0032982	myosin filament	GO:CC	7.16826E-12	1
KEGG:04520	Adherens junction	KEGG	1.23243E-11	1
KEGG:05321	Inflammatory bowel disease (IBD)	KEGG	1.29151E-11	1
GO:1902287	semaphorin-plexin signaling pathway involved in axon guidance	GO:BP	1.99814E-11	1
GO:0016601	Rac protein signal transduction	GO:BP	2.01013E-11	1
GO:0030336	negative regulation of cell migration	GO:BP	2.49148E-11	1
GO:0090630	activation of GTPase activity	GO:BP	3.50886E-11	1
GO:0044331	cell-cell adhesion mediated by cadherin	GO:BP	4.0984E-11	1
GO:0045214	sarcomere organization	GO:BP	5.65967E-11	1
GO:0043525	positive regulation of neuron apoptotic process	GO:BP	6.18404E-11	1
GO:0043524	negative regulation of neuron apoptotic process	GO:BP	7.77106E-11	1
KEGG:04658	Th1 and Th2 cell differentiation	KEGG	8.27435E-11	1
GO:0014911	positive regulation of smooth muscle cell migration	GO:BP	8.33437E-11	1
KEGG:04080	Neuroactive ligand-receptor interaction	KEGG	2.583E-241	2
GO:0007218	neuropeptide signaling pathway	GO:BP	4.93402E-94	2
KEGG:04062	Chemokine signaling pathway	KEGG	7.15277E-51	2
GO:0070098	chemokine-mediated signaling pathway	GO:BP	8.62748E-47	2
KEGG:04020	Calcium signaling pathway	KEGG	3.40923E-44	2
KEGG:04024	cAMP signaling pathway	KEGG	6.09448E-42	2
GO:0005834	heterotrimeric G-protein complex	GO:CC	1.66751E-36	2
KEGG:04725	Cholinergic synapse	KEGG	5.60395E-36	2
KEGG:04724	Glutamatergic synapse	KEGG	9.0408E-32	2
KEGG:05032	Morphine addiction	KEGG	2.51295E-31	2
KEGG:04713	Circadian entrainment	KEGG	1.47701E-30	2
GO:0051482	positive regulation of cytosolic calcium ion concentration involved in phospholipase C-activating G protein-coupled signaling pathway	GO:BP	3.68049E-30	2
KEGG:04726	Serotonergic synapse	KEGG	2.75485E-29	2
GO:0070374	positive regulation of ERK1 and ERK2 cascade	GO:BP	3.56726E-29	2
GO:0001965	G-protein alpha-subunit binding	GO:MF	3.68767E-29	2
GO:0019722	calcium-mediated signaling	GO:BP	7.90667E-29	2
KEGG:04061	Viral protein interaction with cytokine and cytokine receptor	KEGG	2.18838E-28	2
GO:0016493	C-C chemokine receptor activity	GO:MF	2.12654E-27	2
GO:0008009	chemokine activity	GO:MF	8.46583E-27	2
GO:0042923	neuropeptide binding	GO:MF	1.24806E-26	2
KEGG:04723	Retrograde endocannabinoid signaling	KEGG	5.51902E-26	2
KEGG:04926	Relaxin signaling pathway	KEGG	1.98542E-25	2
KEGG:04727	GABAergic synapse	KEGG	7.93016E-24	2
GO:0043950	positive regulation of cAMP-mediated signaling	GO:BP	3.85012E-23	2
GO:0004993	G protein-coupled serotonin receptor activity	GO:MF	1.23295E-21	2
KEGG:04270	Vascular smooth muscle contraction	KEGG	5.89491E-21	2

KEGG:04921	Oxytocin signaling pathway	KEGG	1.95836E-20	2
KEGG:04728	Dopaminergic synapse	KEGG	2.91886E-20	2
GO:0031683	G-protein beta/gamma-subunit complex binding	GO:MF	3.1168E-20	2
GO:0005184	neuropeptide hormone activity	GO:MF	3.1168E-20	2
KEGG:05163	Human cytomegalovirus infection	KEGG	1.87606E-17	2
KEGG:04924	Renin secretion	KEGG	1.97283E-17	2
GO:0007190	activation of adenylate cyclase activity	GO:BP	4.48188E-17	2
KEGG:04911	Insulin secretion	KEGG	7.02547E-17	2
KEGG:04072	Phospholipase D signaling pathway	KEGG	7.04935E-17	2
KEGG:04971	Gastric acid secretion	KEGG	1.08039E-16	2
GO:0045907	positive regulation of vasoconstriction	GO:BP	1.26436E-16	2
KEGG:04261	Adrenergic signaling in cardiomyocytes	KEGG	3.20928E-16	2
KEGG:04742	Taste transduction	KEGG	4.78388E-16	2
GO:0031681	G-protein beta-subunit binding	GO:MF	2.62037E-15	2
GO:0051281	positive regulation of release of sequestered calcium ion into cytosol	GO:BP	4.08315E-15	2
KEGG:04022	cGMP-PKG signaling pathway	KEGG	1.11293E-14	2
KEGG:04540	Gap junction	KEGG	1.80181E-14	2
KEGG:04923	Regulation of lipolysis in adipocytes	KEGG	2.13015E-14	2
GO:0008331	high voltage-gated calcium channel activity	GO:MF	2.85333E-14	2
GO:0035815	positive regulation of renal sodium excretion	GO:BP	3.19112E-14	2
GO:0061844	antimicrobial humoral immune response mediated by antimicrobial peptide	GO:BP	6.68091E-14	2
GO:0043547	positive regulation of GTPase activity	GO:BP	7.37851E-14	2
KEGG:04371	Apelin signaling pathway	KEGG	7.64387E-14	2
GO:0031680	G-protein beta/gamma-subunit complex	GO:CC	1.60075E-13	2
GO:0007613	memory	GO:BP	3.31448E-13	2
KEGG:04927	Cortisol synthesis and secretion	KEGG	6.77636E-13	2
GO:0006171	cAMP biosynthetic process	GO:BP	1.4749E-12	2
GO:0048020	CCR chemokine receptor binding	GO:MF	7.36471E-12	2
KEGG:05414	Dilated cardiomyopathy (DCM)	KEGG	1.03704E-11	2
KEGG:04929	GnRH secretion	KEGG	1.38368E-11	2
GO:0045745	positive regulation of G protein-coupled receptor signaling pathway	GO:BP	3.64634E-11	2
GO:0004966	galanin receptor activity	GO:MF	5.18115E-11	2
GO:0043204	perikaryon	GO:CC	7.85956E-11	2
GO:0035025	positive regulation of Rho protein signal transduction	GO:BP	8.35112E-11	2
KEGG:04915	Estrogen signaling pathway	KEGG	9.48765E-11	2
KEGG:01100	Metabolic pathways	KEGG	0	3
KEGG:00190	Oxidative phosphorylation	KEGG	7.50172E-97	3
KEGG:01200	Carbon metabolism	KEGG	7.16902E-81	3
KEGG:05012	Parkinson disease	KEGG	1.31514E-63	3
KEGG:00230	Purine metabolism	KEGG	2.29564E-57	3
KEGG:04714	Thermogenesis	KEGG	1.0818E-56	3
GO:0005747	mitochondrial respiratory chain complex I	GO:CC	2.47684E-48	3
KEGG:04146	Peroxisome	KEGG	5.40693E-48	3
KEGG:01230	Biosynthesis of amino acids	KEGG	3.70959E-47	3
GO:0005524	ATP binding	GO:MF	5.70489E-47	3
GO:0032981	mitochondrial respiratory chain complex I assembly	GO:BP	3.27269E-45	3
KEGG:00970	Aminoacyl-tRNA biosynthesis	KEGG	5.40812E-43	3
KEGG:00240	Pyrimidine metabolism	KEGG	5.47673E-41	3
KEGG:05016	Huntington disease	KEGG	4.02628E-39	3
KEGG:05010	Alzheimer disease	KEGG	8.44759E-36	3
KEGG:00010	Glycolysis / Gluconeogenesis	KEGG	5.8254E-34	3
GO:0005829	cytosol	GO:CC	1.50472E-32	3
KEGG:04932	Non-alcoholic fatty liver disease (NAFLD)	KEGG	2.37743E-30	3
KEGG:00620	Pyruvate metabolism	KEGG	4.7344E-30	3

KEGG:00640	Propanoate metabolism	KEGG	2.47094E-29	3
KEGG:00250	Alanine, aspartate and glutamate metabolism	KEGG	4.49917E-29	3
KEGG:00280	Valine, leucine and isoleucine degradation	KEGG	6.68623E-28	3
GO:0005758	mitochondrial intermembrane space	GO:CC	1.50603E-27	3
GO:0016597	amino acid binding	GO:MF	9.70016E-27	3
GO:0008137	NADH dehydrogenase (ubiquinone) activity	GO:MF	1.59559E-26	3
GO:0006099	tricarboxylic acid cycle	GO:BP	2.53899E-26	3
KEGG:00410	beta-Alanine metabolism	KEGG	3.41825E-25	3
KEGG:00630	Glyoxylate and dicarboxylate metabolism	KEGG	3.27741E-24	3
KEGG:00270	Cysteine and methionine metabolism	KEGG	1.71802E-23	3
GO:0042803	protein homodimerization activity	GO:MF	5.31102E-23	3
KEGG:00380	Tryptophan metabolism	KEGG	3.02989E-22	3
GO:0043209	myelin sheath	GO:CC	1.46479E-21	3
KEGG:00260	Glycine, serine and threonine metabolism	KEGG	1.93497E-21	3
GO:0051289	protein homotetramerization	GO:BP	1.84319E-20	3
KEGG:00020	Citrate cycle (TCA cycle)	KEGG	4.81298E-19	3
GO:0004129	cytochrome-c oxidase activity	GO:MF	5.36417E-19	3
GO:0030170	pyridoxal phosphate binding	GO:MF	1.0174E-18	3
KEGG:01212	Fatty acid metabolism	KEGG	7.03064E-18	3
GO:0006541	glutamine metabolic process	GO:BP	1.02509E-17	3
GO:0005782	peroxisomal matrix	GO:CC	1.47879E-17	3
GO:0000287	magnesium ion binding	GO:MF	3.5976E-17	3
KEGG:00071	Fatty acid degradation	KEGG	3.94388E-17	3
KEGG:00330	Arginine and proline metabolism	KEGG	4.4514E-17	3
KEGG:00030	Pentose phosphate pathway	KEGG	1.31943E-16	3
GO:0046961	proton-transporting ATPase activity, rotational mechanism	GO:MF	5.65534E-16	3
KEGG:00670	One carbon pool by folate	KEGG	1.90214E-15	3
GO:0004550	nucleoside diphosphate kinase activity	GO:MF	2.24654E-15	3
GO:0016471	vacuolar proton-transporting V-type ATPase complex	GO:CC	4.07885E-15	3
KEGG:00760	Nicotinate and nicotinamide metabolism	KEGG	1.38377E-14	3
KEGG:00350	Tyrosine metabolism	KEGG	1.47125E-14	3
KEGG:00650	Butanoate metabolism	KEGG	1.73252E-14	3
GO:0008483	transaminase activity	GO:MF	3.15081E-14	3
KEGG:00340	Histidine metabolism	KEGG	6.51216E-14	3
KEGG:00480	Glutathione metabolism	KEGG	1.84117E-13	3
GO:0016831	carboxy-lyase activity	GO:MF	2.46622E-13	3
KEGG:00770	Pantothenate and CoA biosynthesis	KEGG	1.42295E-12	3
GO:0030150	protein import into mitochondrial matrix	GO:BP	2.84517E-12	3
KEGG:04966	Collecting duct acid secretion	KEGG	3.60288E-12	3
KEGG:00051	Fructose and mannose metabolism	KEGG	4.25888E-12	3
GO:0035999	tetrahydrofolate interconversion	GO:BP	5.83642E-12	3
KEGG:01210	2-Oxocarboxylic acid metabolism	KEGG	8.17373E-12	3
KEGG:00220	Arginine biosynthesis	KEGG	8.17373E-12	3
GO:0017101	aminoacyl-tRNA synthetase multienzyme complex	GO:CC	1.25649E-11	3
GO:0015986	ATP synthesis coupled proton transport	GO:BP	1.84231E-11	3
GO:0046835	carbohydrate phosphorylation	GO:BP	2.07059E-11	3
KEGG:00052	Galactose metabolism	KEGG	3.08099E-11	3
KEGG:00500	Starch and sucrose metabolism	KEGG	3.08099E-11	3
GO:0006002	fructose 6-phosphate metabolic process	GO:BP	3.90138E-11	3
GO:0006103	2-oxoglutarate metabolic process	GO:BP	4.53948E-11	3
GO:0032482	Rab protein signal transduction	GO:BP	1.69599E-55	4
GO:0005525	GTP binding	GO:MF	5.44405E-49	4
GO:0000139	Golgi membrane	GO:CC	8.0849E-45	4
GO:0003924	GTPase activity	GO:MF	2.7619E-44	4
GO:0005814	centriole	GO:CC	2.67798E-40	4
GO:0036064	ciliary basal body	GO:CC	2.13912E-38	4

GO:0006890	retrograde vesicle-mediated transport, Golgi to endoplasmic reticulum	GO:BP	2.17701E-31	4
GO:0005829	cytosol	GO:CC	9.02588E-31	4
GO:0017112	Rab guanyl-nucleotide exchange factor activity	GO:MF	1.73369E-29	4
GO:0006891	intra-Golgi vesicle-mediated transport	GO:BP	2.94248E-27	4
GO:0008574	ATP-dependent microtubule motor activity, plus-end-directed	GO:MF	7.02954E-22	4
GO:0042147	retrograde transport, endosome to Golgi	GO:BP	1.82404E-20	4
GO:0006904	vesicle docking involved in exocytosis	GO:BP	1.9764E-18	4
GO:0005868	cytoplasmic dynein complex	GO:CC	3.30872E-18	4
KEGG:04114	Oocyte meiosis	KEGG	1.65687E-17	4
GO:0019003	GDP binding	GO:MF	1.78284E-17	4
GO:0097546	ciliary base	GO:CC	3.99696E-17	4
KEGG:04130	SNARE interactions in vesicular transport	KEGG	8.84551E-17	4
GO:0032391	photoreceptor connecting cilium	GO:CC	1.22063E-16	4
GO:0005801	cis-Golgi network	GO:CC	2.57778E-16	4
GO:0005484	SNAP receptor activity	GO:MF	2.64407E-16	4
KEGG:04962	Vasopressin-regulated water reabsorption	KEGG	3.42084E-16	4
GO:0045505	dynein intermediate chain binding	GO:MF	6.55214E-16	4
GO:0031201	SNARE complex	GO:CC	8.61422E-16	4
GO:0048471	perinuclear region of cytoplasm	GO:CC	9.2607E-16	4
GO:0071539	protein localization to centrosome	GO:BP	1.29377E-15	4
GO:0030992	intraciliary transport particle B	GO:CC	1.34763E-15	4
GO:0001518	voltage-gated sodium channel complex	GO:CC	4.72387E-15	4
GO:0030126	COPI vesicle coat	GO:CC	1.31329E-14	4
GO:0031489	myosin V binding	GO:MF	2.81166E-14	4
GO:0030127	COPII vesicle coat	GO:CC	9.44957E-14	4
GO:0019905	syntaxin binding	GO:MF	4.72445E-12	4
GO:0036038	MKS complex	GO:CC	5.86386E-12	4
GO:0090110	COPII-coated vesicle cargo loading	GO:BP	1.11518E-11	4
GO:0051959	dynein light intermediate chain binding	GO:MF	1.55322E-11	4
GO:0000242	pericentriolar material	GO:CC	2.06867E-11	4
GO:0031122	cytoplasmic microtubule organization	GO:BP	2.16096E-11	4
GO:0043015	gamma-tubulin binding	GO:MF	2.36248E-11	4
GO:0035371	microtubule plus-end	GO:CC	2.40385E-11	4
GO:0008569	ATP-dependent microtubule motor activity, minus-end-directed	GO:MF	2.53727E-11	4
GO:0031105	septin complex	GO:CC	3.65835E-11	4
GO:0097431	mitotic spindle pole	GO:CC	8.08337E-11	4
GO:1902476	chloride transmembrane transport	GO:BP	2.00865E-34	5
GO:0004890	GABA-A receptor activity	GO:MF	1.20208E-30	5
GO:1902711	GABA-A receptor complex	GO:CC	1.94149E-30	5
GO:0022851	GABA-gated chloride ion channel activity	GO:MF	3.28141E-30	5
GO:1904315	transmitter-gated ion channel activity involved in regulation of postsynaptic membrane potential	GO:MF	5.46223E-30	5
GO:0007214	gamma-aminobutyric acid signaling pathway	GO:BP	2.11731E-27	5
GO:0098982	GABA-ergic synapse	GO:CC	1.82843E-23	5
KEGG:05033	Nicotine addiction	KEGG	1.88492E-22	5
GO:0008503	benzodiazepine receptor activity	GO:MF	2.39127E-20	5
KEGG:04727	GABAergic synapse	KEGG	3.79753E-20	5
GO:0099060	integral component of postsynaptic specialization membrane	GO:CC	3.00052E-18	5
GO:0051932	synaptic transmission, GABAergic	GO:BP	4.41244E-18	5
KEGG:05032	Morphine addiction	KEGG	6.63569E-18	5
KEGG:04080	Neuroactive ligand-receptor interaction	KEGG	1.67945E-17	5
GO:0030054	cell junction	GO:CC	2.05835E-15	5
KEGG:04723	Retrograde endocannabinoid signaling	KEGG	2.91983E-15	5

GO:0032590	dendrite membrane	GO:CC	2.68165E-14	5
GO:0016934	extracellularly glycine-gated chloride channel activity	GO:MF	1.67442E-12	5
GO:0071420	cellular response to histamine	GO:BP	3.08593E-11	5
GO:0001228	DNA-binding transcription activator activity, RNA polymerase II-specific	GO:MF	7.08221E-85	6
GO:0003714	transcription corepressor activity	GO:MF	1.37665E-57	6
GO:0003707	steroid hormone receptor activity	GO:MF	1.23462E-42	6
GO:0042826	histone deacetylase binding	GO:MF	2.28992E-41	6
GO:0016592	mediator complex	GO:CC	1.20263E-39	6
GO:0046982	protein heterodimerization activity	GO:MF	7.45883E-38	6
GO:0000980	RNA polymerase II distal enhancer sequence-specific DNA binding	GO:MF	1.20039E-34	6
GO:0001227	DNA-binding transcription repressor activity, RNA polymerase II-specific	GO:MF	2.17259E-34	6
KEGG:04330	Notch signaling pathway	KEGG	2.62849E-34	6
GO:0004879	nuclear receptor activity	GO:MF	1.40043E-32	6
KEGG:05202	Transcriptional misregulation in cancer	KEGG	9.9941E-30	6
GO:0008270	zinc ion binding	GO:MF	2.66899E-29	6
GO:1990841	promoter-specific chromatin binding	GO:MF	4.8588E-23	6
GO:0001085	RNA polymerase II transcription factor binding	GO:MF	6.76715E-23	6
GO:0070888	E-box binding	GO:MF	5.28892E-22	6
KEGG:05034	Alcoholism	KEGG	8.67042E-22	6
KEGG:04919	Thyroid hormone signaling pathway	KEGG	1.41937E-20	6
GO:0035914	skeletal muscle cell differentiation	GO:BP	1.06095E-19	6
GO:0001102	RNA polymerase II activating transcription factor binding	GO:MF	1.19092E-18	6
GO:0035064	methylated histone binding	GO:MF	1.33837E-18	6
GO:0005719	nuclear euchromatin	GO:CC	3.03416E-18	6
GO:0035102	PRC1 complex	GO:CC	1.2979E-17	6
KEGG:05203	Viral carcinogenesis	KEGG	2.43868E-17	6
GO:0016605	PML body	GO:CC	1.1324E-16	6
GO:0016581	NuRD complex	GO:CC	1.97934E-16	6
GO:0071565	nBAF complex	GO:CC	2.59458E-16	6
GO:0016514	SWI/SNF complex	GO:CC	5.44055E-16	6
GO:0032922	circadian regulation of gene expression	GO:BP	9.30399E-16	6
GO:0043981	histone H4-K5 acetylation	GO:BP	1.17254E-15	6
GO:0043982	histone H4-K8 acetylation	GO:BP	1.17254E-15	6
GO:0016607	nuclear speck	GO:CC	2.26472E-15	6
GO:0071339	MLL1 complex	GO:CC	2.5824E-15	6
GO:0035019	somatic stem cell population maintenance	GO:BP	4.844E-15	6
GO:0035098	ESC/E(Z) complex	GO:CC	2.82958E-14	6
GO:0031011	Ino80 complex	GO:CC	6.91762E-14	6
KEGG:05322	Systemic lupus erythematosus	KEGG	1.08037E-13	6
KEGG:05225	Hepatocellular carcinoma	KEGG	1.41484E-13	6
GO:0043984	histone H4-K16 acetylation	GO:BP	2.14329E-13	6
GO:0046965	retinoid X receptor binding	GO:MF	2.29609E-13	6
GO:0070932	histone H3 deacetylation	GO:BP	2.4608E-13	6
GO:0043968	histone H2A acetylation	GO:BP	9.08479E-13	6
GO:0043065	positive regulation of apoptotic process	GO:BP	1.24527E-12	6
GO:0016580	Sin3 complex	GO:CC	1.28212E-12	6
GO:0071564	npBAF complex	GO:CC	2.06165E-12	6
GO:0002039	p53 binding	GO:MF	2.73398E-12	6
GO:0000788	nuclear nucleosome	GO:CC	3.82489E-12	6
GO:0006335	DNA replication-dependent nucleosome assembly	GO:BP	4.77521E-12	6
KEGG:04110	Cell cycle	KEGG	7.67608E-12	6
KEGG:04550	Signaling pathways regulating pluripotency of stem cells	KEGG	8.85724E-12	6
GO:0006336	DNA replication-independent nucleosome assembly	GO:BP	1.22241E-11	6
GO:0006337	nucleosome disassembly	GO:BP	1.49965E-11	6

GO:0016584	nucleosome positioning	GO:BP	1.49965E-11	6
GO:0008013	beta-catenin binding	GO:MF	1.81203E-11	6
KEGG:01522	Endocrine resistance	KEGG	2.33219E-11	6
GO:0000812	Swr1 complex	GO:CC	2.36023E-11	6
GO:0042800	histone methyltransferase activity (H3-K4 specific)	GO:MF	5.06593E-11	6
GO:0032041	NAD-dependent histone deacetylase activity (H3-K14 specific)	GO:MF	5.92776E-11	6
KEGG:04064	NF-kappa B signaling pathway	KEGG	7.61295E-59	7
KEGG:03050	Proteasome	KEGG	5.03117E-55	7
GO:0043123	positive regulation of I-kappaB kinase/NF-kappaB signaling	GO:BP	2.93153E-54	7
KEGG:05169	Epstein-Barr virus infection	KEGG	8.29738E-42	7
GO:0051092	positive regulation of NF-kappaB transcription factor activity	GO:BP	2.10774E-40	7
KEGG:04620	Toll-like receptor signaling pathway	KEGG	7.43693E-32	7
GO:0004298	threonine-type endopeptidase activity	GO:MF	7.42708E-28	7
KEGG:04668	TNF signaling pathway	KEGG	6.6166E-27	7
GO:0010499	proteasomal ubiquitin-independent protein catabolic process	GO:BP	2.81865E-26	7
KEGG:04621	NOD-like receptor signaling pathway	KEGG	6.93623E-26	7
GO:0005164	tumor necrosis factor receptor binding	GO:MF	2.55667E-24	7
KEGG:04622	RIG-I-like receptor signaling pathway	KEGG	1.22448E-23	7
KEGG:05161	Hepatitis B	KEGG	4.46085E-23	7
GO:0043161	proteasome-mediated ubiquitin-dependent protein catabolic process	GO:BP	5.19641E-21	7
KEGG:05162	Measles	KEGG	5.73327E-21	7
KEGG:04010	MAPK signaling pathway	KEGG	4.86218E-19	7
GO:0042802	identical protein binding	GO:MF	9.46053E-18	7
KEGG:04210	Apoptosis	KEGG	1.03473E-17	7
KEGG:04217	Necroptosis	KEGG	1.10099E-17	7
GO:0032088	negative regulation of NF-kappaB transcription factor activity	GO:BP	1.9056E-17	7
GO:0019901	protein kinase binding	GO:MF	6.09515E-17	7
KEGG:05164	Influenza A	KEGG	1.11307E-16	7
KEGG:04340	Hedgehog signaling pathway	KEGG	2.13223E-16	7
GO:0019774	proteasome core complex, beta-subunit complex	GO:CC	3.23821E-15	7
GO:0032760	positive regulation of tumor necrosis factor production	GO:BP	3.9069E-15	7
KEGG:04657	IL-17 signaling pathway	KEGG	4.11346E-14	7
KEGG:05145	Toxoplasmosis	KEGG	7.04823E-14	7
GO:0031625	ubiquitin protein ligase binding	GO:MF	1.3546E-13	7
GO:0050700	CARD domain binding	GO:MF	4.4848E-13	7
GO:0002755	MyD88-dependent toll-like receptor signaling pathway	GO:BP	6.14073E-13	7
GO:0007252	I-kappaB phosphorylation	GO:BP	6.14073E-13	7
GO:0008540	proteasome regulatory particle, base subcomplex	GO:CC	8.77182E-13	7
KEGG:05135	Yersinia infection	KEGG	1.23769E-12	7
KEGG:05142	Chagas disease (American trypanosomiasis)	KEGG	1.42624E-12	7
GO:2001238	positive regulation of extrinsic apoptotic signaling pathway	GO:BP	1.57425E-12	7
KEGG:05217	Basal cell carcinoma	KEGG	2.6551E-12	7
GO:0007250	activation of NF-kappaB-inducing kinase activity	GO:BP	2.70788E-12	7
GO:0071260	cellular response to mechanical stimulus	GO:BP	3.22618E-12	7
KEGG:05160	Hepatitis C	KEGG	7.60867E-12	7
GO:0019773	proteasome core complex, alpha-subunit complex	GO:CC	8.25845E-12	7
KEGG:04380	Osteoclast differentiation	KEGG	1.55643E-11	7
KEGG:05235	PD-L1 expression and PD-1 checkpoint pathway in cancer	KEGG	2.14885E-11	7
KEGG:04623	Cytosolic DNA-sensing pathway	KEGG	2.47349E-11	7
GO:0005524	ATP binding	GO:MF	3.03497E-11	7
GO:0050729	positive regulation of inflammatory response	GO:BP	4.27539E-11	7

KEGG:05222	Small cell lung cancer	KEGG	4.66181E-11	7
KEGG:03040	Spliceosome	KEGG	4.3478E-152	8
GO:0016607	nuclear speck	GO:CC	5.90546E-78	8
GO:0071005	U2-type precatalytic spliceosome	GO:CC	2.64265E-59	8
KEGG:03013	RNA transport	KEGG	2.43202E-49	8
KEGG:03022	Basal transcription factors	KEGG	4.27996E-43	8
GO:0071007	U2-type catalytic step 2 spliceosome	GO:CC	2.1861E-38	8
GO:0005689	U12-type spliceosomal complex	GO:CC	1.02773E-35	8
GO:0005669	transcription factor TFIID complex	GO:CC	1.91584E-33	8
KEGG:03015	mRNA surveillance pathway	KEGG	1.27804E-32	8
GO:0046540	U4/U6 x U5 tri-snRNP complex	GO:CC	2.62572E-32	8
GO:0000381	regulation of alternative mRNA splicing, via spliceosome	GO:BP	7.72621E-26	8
GO:0005686	U2 snRNP	GO:CC	4.93856E-25	8
KEGG:03020	RNA polymerase	KEGG	1.49046E-23	8
GO:0034719	SMN-Sm protein complex	GO:CC	3.61691E-23	8
GO:0005685	U1 snRNP	GO:CC	3.61691E-23	8
GO:0017056	structural constituent of nuclear pore	GO:MF	4.63923E-22	8
GO:0005666	RNA polymerase III complex	GO:CC	5.25436E-21	8
GO:0015030	Cajal body	GO:CC	6.33796E-20	8
GO:0045292	mRNA cis splicing, via spliceosome	GO:BP	5.94796E-19	8
GO:0005682	U5 snRNP	GO:CC	7.12039E-19	8
GO:0032968	positive regulation of transcription elongation from RNA polymerase II promoter	GO:BP	9.65264E-19	8
GO:0001056	RNA polymerase III activity	GO:MF	7.31256E-18	8
GO:0071004	U2-type prespliceosome	GO:CC	6.5158E-17	8
GO:0008353	RNA polymerase II CTD heptapeptide repeat kinase activity	GO:MF	1.7732E-16	8
GO:0000974	Prp19 complex	GO:CC	1.77838E-16	8
GO:0005665	RNA polymerase II, core complex	GO:CC	8.25658E-16	8
GO:0016251	RNA polymerase II general transcription initiation factor activity	GO:MF	1.14039E-15	8
GO:0006606	protein import into nucleus	GO:BP	4.3141E-14	8
GO:0048025	negative regulation of mRNA splicing, via spliceosome	GO:BP	7.88583E-14	8
GO:0005687	U4 snRNP	GO:CC	8.65804E-14	8
GO:0071006	U2-type catalytic step 1 spliceosome	GO:CC	1.76218E-13	8
GO:0006376	mRNA splice site selection	GO:BP	4.43206E-13	8
GO:0005847	mRNA cleavage and polyadenylation specificity factor complex	GO:CC	1.03751E-12	8
GO:1990446	U1 snRNP binding	GO:MF	4.66826E-12	8
GO:0001055	RNA polymerase II activity	GO:MF	4.66826E-12	8
GO:0000993	RNA polymerase II complex binding	GO:MF	9.17382E-12	8
GO:0032797	SMN complex	GO:CC	3.07025E-11	8
GO:0032039	integrator complex	GO:CC	6.33115E-11	8
KEGG:03460	Fanconi anemia pathway	KEGG	6.74678E-72	9
KEGG:03030	DNA replication	KEGG	7.16352E-55	9
KEGG:03440	Homologous recombination	KEGG	1.95684E-44	9
KEGG:03420	Nucleotide excision repair	KEGG	4.62863E-41	9
GO:0003697	single-stranded DNA binding	GO:MF	1.68344E-38	9
GO:0036297	interstrand cross-link repair	GO:BP	3.46204E-33	9
KEGG:03410	Base excision repair	KEGG	7.75773E-33	9
GO:0017116	single-stranded DNA helicase activity	GO:MF	2.60819E-29	9
KEGG:03430	Mismatch repair	KEGG	1.53377E-27	9
GO:0003688	DNA replication origin binding	GO:MF	3.64721E-26	9
GO:0003887	DNA-directed DNA polymerase activity	GO:MF	1.44805E-24	9
GO:0031297	replication fork processing	GO:BP	2.11693E-23	9
GO:0006298	mismatch repair	GO:BP	4.60123E-19	9
GO:0000727	double-strand break repair via break-induced replication	GO:BP	3.00535E-18	9
GO:0043138	3'-5' DNA helicase activity	GO:MF	3.39847E-17	9

GO:0035861	site of double-strand break	GO:CC	7.72333E-17	9
GO:0000784	nuclear chromosome, telomeric region	GO:CC	7.88817E-17	9
GO:0072546	ER membrane protein complex	GO:CC	1.97432E-15	9
GO:0003689	DNA clamp loader activity	GO:MF	3.184E-15	9
GO:0005524	ATP binding	GO:MF	6.63715E-15	9
GO:0003682	chromatin binding	GO:MF	1.4523E-14	9
GO:0051539	4 iron, 4 sulfur cluster binding	GO:MF	1.47251E-14	9
GO:0031390	Ctf18 RFC-like complex	GO:CC	1.98717E-13	9
GO:0008821	crossover junction endodeoxyribonuclease activity	GO:MF	2.58577E-13	9
GO:0031573	intra-S DNA damage checkpoint	GO:BP	6.90061E-13	9
GO:0000400	four-way junction DNA binding	GO:MF	1.56298E-12	9
GO:0006303	double-strand break repair via nonhomologous end joining	GO:BP	3.44828E-12	9
GO:0043240	Fanconi anaemia nuclear complex	GO:CC	4.67621E-12	9
GO:0006268	DNA unwinding involved in DNA replication	GO:BP	1.3397E-11	9
GO:0017108	5'-flap endonuclease activity	GO:MF	2.092E-11	9
KEGG:04141	Protein processing in endoplasmic reticulum	KEGG	7.02076E-11	9
KEGG:04350	TGF-beta signaling pathway	KEGG	4.8933E-46	10
GO:0010862	positive regulation of pathway-restricted SMAD protein phosphorylation	GO:BP	5.47727E-38	10
KEGG:04610	Complement and coagulation cascades	KEGG	3.32762E-37	10
GO:0008083	growth factor activity	GO:MF	4.09752E-29	10
KEGG:00532	Glycosaminoglycan biosynthesis - chondroitin sulfate / dermatan sulfate	KEGG	8.41615E-28	10
KEGG:00534	Glycosaminoglycan biosynthesis - heparan sulfate / heparin	KEGG	5.22925E-26	10
GO:0004252	serine-type endopeptidase activity	GO:MF	2.39222E-20	10
GO:0048185	activin binding	GO:MF	2.05609E-19	10
GO:0022848	acetylcholine-gated cation-selective channel activity	GO:MF	5.31076E-19	10
GO:0005892	acetylcholine-gated channel complex	GO:CC	1.57346E-17	10
GO:0008201	heparin binding	GO:MF	4.47395E-17	10
KEGG:04512	ECM-receptor interaction	KEGG	1.32648E-16	10
GO:0005509	calcium ion binding	GO:MF	3.27452E-16	10
GO:0042166	acetylcholine binding	GO:MF	3.90415E-15	10
GO:0030206	chondroitin sulfate biosynthetic process	GO:BP	1.65012E-13	10
GO:0005125	cytokine activity	GO:MF	2.86556E-13	10
GO:0000139	Golgi membrane	GO:CC	7.39539E-13	10
GO:0010951	negative regulation of endopeptidase activity	GO:BP	1.38424E-12	10
GO:0015464	acetylcholine receptor activity	GO:MF	2.22379E-12	10
GO:0005178	integrin binding	GO:MF	5.25917E-12	10
GO:0005796	Golgi lumen	GO:CC	7.4518E-12	10
GO:0050431	transforming growth factor beta binding	GO:MF	9.80927E-12	10
GO:0034361	very-low-density lipoprotein particle	GO:CC	1.96817E-11	10
GO:0007271	synaptic transmission, cholinergic	GO:BP	2.26852E-11	10
KEGG:03010	Ribosome	KEGG	7.72867E-86	11
GO:0003735	structural constituent of ribosome	GO:MF	1.03251E-83	11
KEGG:03008	Ribosome biogenesis in eukaryotes	KEGG	8.15845E-62	11
GO:0022625	cytosolic large ribosomal subunit	GO:CC	6.08613E-59	11
GO:0022627	cytosolic small ribosomal subunit	GO:CC	6.13469E-47	11
GO:0003743	translation initiation factor activity	GO:MF	8.47435E-45	11
GO:0032040	small-subunit processome	GO:CC	3.79091E-40	11
KEGG:03013	RNA transport	KEGG	7.694E-40	11
KEGG:03018	RNA degradation	KEGG	3.31258E-38	11
GO:0042788	polysomal ribosome	GO:CC	4.16161E-36	11
GO:0030687	preribosome, large subunit precursor	GO:CC	2.64676E-27	11
GO:0000027	ribosomal large subunit assembly	GO:BP	1.53724E-23	11
GO:0000184	nuclear-transcribed mRNA catabolic process, nonsense-mediated decay	GO:BP	2.06786E-20	11

GO:0000176	nuclear exosome (RNase complex)	GO:CC	3.67107E-20	11
GO:0043022	ribosome binding	GO:MF	4.66598E-19	11
KEGG:03060	Protein export	KEGG	9.41493E-19	11
GO:0016282	eukaryotic 43S preinitiation complex	GO:CC	1.59673E-18	11
GO:0001732	formation of cytoplasmic translation initiation complex	GO:BP	2.02299E-18	11
GO:0033290	eukaryotic 48S preinitiation complex	GO:CC	1.14448E-17	11
GO:0030688	preribosome, small subunit precursor	GO:CC	6.56304E-17	11
GO:0045727	positive regulation of translation	GO:BP	1.11313E-16	11
GO:0004526	ribonuclease P activity	GO:MF	2.17191E-16	11
GO:0003724	RNA helicase activity	GO:MF	6.58135E-16	11
GO:0000028	ribosomal small subunit assembly	GO:BP	1.75915E-15	11
GO:0031369	translation initiation factor binding	GO:MF	7.50781E-15	11
GO:0033204	ribonuclease P RNA binding	GO:MF	1.02815E-14	11
GO:0000463	maturation of LSU-rRNA from tricistronic rRNA transcript (SSU-rRNA, 5.8S rRNA, LSU-rRNA)	GO:BP	1.56509E-14	11
GO:0001682	tRNA 5'-leader removal	GO:BP	1.95609E-14	11
GO:0034427	nuclear-transcribed mRNA catabolic process, exonucleolytic, 3'-5'	GO:BP	7.55105E-14	11
GO:0000177	cytoplasmic exosome (RNase complex)	GO:CC	7.98695E-14	11
GO:0000932	P-body	GO:CC	1.21443E-13	11
GO:0006614	SRP-dependent cotranslational protein targeting to membrane	GO:BP	1.59708E-12	11
GO:0010494	cytoplasmic stress granule	GO:CC	2.57471E-12	11
GO:0043928	exonucleolytic catabolism of deadenylated mRNA	GO:BP	3.42024E-12	11
GO:0071028	nuclear mRNA surveillance	GO:BP	4.78607E-12	11
GO:0071541	eukaryotic translation initiation factor 3 complex, eIF3m	GO:CC	2.13808E-11	11
GO:0008250	oligosaccharyltransferase complex	GO:CC	2.5104E-11	11
KEGG:04120	Ubiquitin mediated proteolysis	KEGG	1.4493E-150	12
GO:0019005	SCF ubiquitin ligase complex	GO:CC	5.58379E-55	12
GO:0061631	ubiquitin conjugating enzyme activity	GO:MF	1.4353E-44	12
GO:0031146	SCF-dependent proteasomal ubiquitin-dependent protein catabolic process	GO:BP	7.3333E-41	12
GO:0070936	protein K48-linked ubiquitination	GO:BP	3.08976E-34	12
GO:0070979	protein K11-linked ubiquitination	GO:BP	2.81747E-33	12
GO:0006513	protein monoubiquitination	GO:BP	4.79127E-26	12
GO:0005680	anaphase-promoting complex	GO:CC	4.87303E-26	12
GO:0031625	ubiquitin protein ligase binding	GO:MF	1.85852E-22	12
GO:0051865	protein autoubiquitination	GO:BP	2.84345E-22	12
GO:0070534	protein K63-linked ubiquitination	GO:BP	4.67977E-20	12
GO:0031463	Cul3-RING ubiquitin ligase complex	GO:CC	3.46248E-19	12
GO:0031624	ubiquitin conjugating enzyme binding	GO:MF	1.75884E-18	12
GO:0005829	cytosol	GO:CC	4.13361E-16	12
GO:0031145	anaphase-promoting complex-dependent catabolic process	GO:BP	9.68609E-16	12
GO:0032436	positive regulation of proteasomal ubiquitin-dependent protein catabolic process	GO:BP	1.10728E-15	12
GO:0000338	protein deneddylation	GO:BP	4.90817E-15	12
GO:0045116	protein neddylation	GO:BP	6.17359E-14	12
GO:0097602	cullin family protein binding	GO:MF	6.04276E-13	12
GO:0051443	positive regulation of ubiquitin-protein transferase activity	GO:BP	6.72832E-13	12
KEGG:04114	Oocyte meiosis	KEGG	7.96239E-12	12
GO:0030020	extracellular matrix structural constituent conferring tensile strength	GO:MF	7.10513E-70	13
KEGG:04974	Protein digestion and absorption	KEGG	5.68511E-58	13
GO:0005615	extracellular space	GO:CC	2.28706E-39	13
GO:0030199	collagen fibril organization	GO:BP	1.90896E-33	13

GO:0004222	metalloendopeptidase activity	GO:MF	5.42123E-27	13
KEGG:04512	ECM-receptor interaction	KEGG	5.29564E-23	13
KEGG:04510	Focal adhesion	KEGG	4.69261E-16	13
GO:0008201	heparin binding	GO:MF	8.415E-14	13
GO:0048407	platelet-derived growth factor binding	GO:MF	2.06137E-11	13
KEGG:05165	Human papillomavirus infection	KEGG	2.19115E-11	13
KEGG:04151	PI3K-Akt signaling pathway	KEGG	2.69514E-11	13
GO:0005587	collagen type IV trimer	GO:CC	5.17841E-11	13
GO:0006508	proteolysis	GO:BP	8.02289E-11	13
GO:0098978	glutamatergic synapse	GO:CC	3.19364E-49	14
KEGG:04144	Endocytosis	KEGG	4.81112E-47	14
KEGG:04310	Wnt signaling pathway	KEGG	5.79149E-45	14
GO:0005109	frizzled binding	GO:MF	3.85988E-39	14
KEGG:04721	Synaptic vesicle cycle	KEGG	6.8311E-27	14
GO:0017147	Wnt-protein binding	GO:MF	7.31719E-27	14
KEGG:05217	Basal cell carcinoma	KEGG	8.20259E-27	14
KEGG:04916	Melanogenesis	KEGG	1.5427E-21	14
GO:0031901	early endosome membrane	GO:CC	3.56195E-20	14
KEGG:04150	mTOR signaling pathway	KEGG	7.74557E-20	14
KEGG:04390	Hippo signaling pathway	KEGG	1.56343E-19	14
KEGG:05224	Breast cancer	KEGG	4.42609E-19	14
KEGG:05226	Gastric cancer	KEGG	5.58875E-19	14
GO:0042813	Wnt-activated receptor activity	GO:MF	8.49255E-19	14
GO:0005802	trans-Golgi network	GO:CC	1.04748E-18	14
GO:0048268	clathrin coat assembly	GO:BP	1.09902E-18	14
KEGG:04934	Cushing syndrome	KEGG	6.49996E-17	14
GO:1905606	regulation of presynapse assembly	GO:BP	1.23243E-16	14
GO:0006886	intracellular protein transport	GO:BP	2.42825E-16	14
KEGG:05225	Hepatocellular carcinoma	KEGG	2.6004E-16	14
GO:0060076	excitatory synapse	GO:CC	6.3562E-16	14
KEGG:04550	Signaling pathways regulating pluripotency of stem cells	KEGG	1.04192E-15	14
GO:0048490	anterograde synaptic vesicle transport	GO:BP	2.2213E-15	14
KEGG:05205	Proteoglycans in cancer	KEGG	9.68319E-15	14
GO:0098685	Schaffer collateral - CA1 synapse	GO:CC	2.01547E-14	14
GO:0035615	clathrin adaptor activity	GO:MF	1.38241E-13	14
GO:0043025	neuronal cell body	GO:CC	3.46245E-13	14
KEGG:04142	Lysosome	KEGG	5.64051E-13	14
GO:0030122	AP-2 adaptor complex	GO:CC	1.25076E-12	14
GO:0005885	Arp2/3 protein complex	GO:CC	1.25076E-12	14
GO:0098686	hippocampal mossy fiber to CA3 synapse	GO:CC	2.47984E-12	14
GO:0051965	positive regulation of synapse assembly	GO:BP	5.52508E-12	14
GO:0030285	integral component of synaptic vesicle membrane	GO:CC	9.97183E-12	14
GO:1904115	axon cytoplasm	GO:CC	6.09626E-11	14
KEGG:05164	Influenza A	KEGG	3.41504E-20	15
KEGG:05169	Epstein-Barr virus infection	KEGG	1.73893E-18	15
KEGG:04612	Antigen processing and presentation	KEGG	3.43115E-18	15
KEGG:05160	Hepatitis C	KEGG	3.34848E-15	15
KEGG:04621	NOD-like receptor signaling pathway	KEGG	1.55568E-14	15
KEGG:05162	Measles	KEGG	1.32356E-13	15
KEGG:05168	Herpes simplex virus 1 infection	KEGG	1.19154E-12	15
GO:0045071	negative regulation of viral genome replication	GO:BP	3.41201E-12	15
KEGG:04622	RIG-I-like receptor signaling pathway	KEGG	6.41315E-12	15
KEGG:04620	Toll-like receptor signaling pathway	KEGG	3.90785E-11	15
KEGG:04142	Lysosome	KEGG	4.67921E-28	17
KEGG:00531	Glycosaminoglycan degradation	KEGG	2.83386E-22	17
KEGG:00520	Amino sugar and nucleotide sugar metabolism	KEGG	1.07037E-16	17
GO:0005615	extracellular space	GO:CC	1.79701E-14	17

KEGG:01100	Metabolic pathways	KEGG	6.11975E-13	17
KEGG:00511	Other glycan degradation	KEGG	6.9896E-13	17
GO:0004364	glutathione transferase activity	GO:MF	2.0828E-11	17
KEGG:00983	Drug metabolism - other enzymes	KEGG	4.47531E-11	17
KEGG:01100	Metabolic pathways	KEGG	2.31072E-70	18
GO:0055114	oxidation-reduction process	GO:BP	9.44105E-63	18
KEGG:00563	Glycosylphosphatidylinositol (GPI)-anchor biosynthesis	KEGG	1.83207E-31	18
GO:0042572	retinol metabolic process	GO:BP	1.39818E-30	18
KEGG:00100	Steroid biosynthesis	KEGG	8.13009E-30	18
KEGG:00140	Steroid hormone biosynthesis	KEGG	8.32029E-27	18
GO:0006695	cholesterol biosynthetic process	GO:BP	1.59265E-25	18
GO:0005506	iron ion binding	GO:MF	5.03987E-25	18
KEGG:00830	Retinol metabolism	KEGG	1.00793E-24	18
GO:0020037	heme binding	GO:MF	5.22534E-21	18
KEGG:00900	Terpenoid backbone biosynthesis	KEGG	4.87718E-20	18
GO:0004745	retinol dehydrogenase activity	GO:MF	8.79021E-18	18
GO:0008395	steroid hydroxylase activity	GO:MF	3.57876E-14	18
GO:0000506	glycosylphosphatidylinositol-N-acetylglucosaminyltransferase (GPI-GnT) complex	GO:CC	9.71528E-14	18
KEGG:00980	Metabolism of xenobiotics by cytochrome P450	KEGG	5.00583E-13	18
KEGG:00510	N-Glycan biosynthesis	KEGG	5.77645E-13	18
GO:0004303	estradiol 17-beta-dehydrogenase activity	GO:MF	8.8929E-13	18
GO:0004806	triglyceride lipase activity	GO:MF	1.76993E-12	18
GO:0097502	mannosylation	GO:BP	8.38182E-12	18
GO:0006703	estrogen biosynthetic process	GO:BP	2.51673E-11	18
KEGG:05204	Chemical carcinogenesis	KEGG	3.97503E-11	18
GO:0008207	C21-steroid hormone metabolic process	GO:BP	5.02756E-11	18
KEGG:00512	Mucin type O-glycan biosynthesis	KEGG	1.87857E-49	20
KEGG:01100	Metabolic pathways	KEGG	3.92514E-47	20
KEGG:00601	Glycosphingolipid biosynthesis - lacto and neolacto series	KEGG	1.91763E-41	20
GO:0004653	polypeptide N-acetylgalactosaminyltransferase activity	GO:MF	1.0335E-29	20
GO:0032580	Golgi cisterna membrane	GO:CC	1.77377E-27	20
GO:0016021	integral component of membrane	GO:CC	4.10907E-27	20
KEGG:00533	Glycosaminoglycan biosynthesis - keratan sulfate	KEGG	8.65711E-24	20
KEGG:00515	Mannose type O-glycan biosynthesis	KEGG	4.2442E-21	20
KEGG:00603	Glycosphingolipid biosynthesis - globo and isoglobo series	KEGG	1.20638E-19	20
KEGG:00604	Glycosphingolipid biosynthesis - ganglio series	KEGG	9.81608E-18	20
GO:0030311	poly-N-acetyllactosamine biosynthetic process	GO:BP	3.58721E-17	20
GO:0008532	N-acetyllactosaminide beta-1,3-N-acetylglucosaminyltransferase activity	GO:MF	2.30878E-15	20
GO:0030246	carbohydrate binding	GO:MF	5.70758E-13	20
GO:0009312	oligosaccharide biosynthetic process	GO:BP	2.19142E-12	20
GO:0018243	protein O-linked glycosylation via threonine	GO:BP	5.50097E-12	20
KEGG:00510	N-Glycan biosynthesis	KEGG	9.54478E-11	20
KEGG:04950	Maturity onset diabetes of the young	KEGG	1.88051E-20	23
GO:0016338	calcium-independent cell-cell adhesion via plasma membrane cell-adhesion molecules	GO:BP	2.13111E-58	25
GO:0005923	bicellular tight junction	GO:CC	5.19675E-52	25
KEGG:04670	Leukocyte transendothelial migration	KEGG	8.83362E-38	25
KEGG:04530	Tight junction	KEGG	6.73441E-37	25
KEGG:05160	Hepatitis C	KEGG	3.15322E-34	25
KEGG:04514	Cell adhesion molecules (CAMs)	KEGG	3.15322E-34	25
GO:0005198	structural molecule activity	GO:MF	2.29095E-25	25
GO:0016328	lateral plasma membrane	GO:CC	2.43721E-15	25
GO:0042802	identical protein binding	GO:MF	2.83021E-15	25
GO:0016327	apicolateral plasma membrane	GO:CC	2.03473E-14	25
KEGG:05150	Staphylococcus aureus infection	KEGG	1.25471E-48	30

KEGG:04915	Estrogen signaling pathway	KEGG	8.92592E-47	30
GO:0045095	keratin filament	GO:CC	1.22979E-44	30
GO:0045109	intermediate filament organization	GO:BP	2.59E-14	30
GO:0005890	sodium:potassium-exchanging ATPase complex	GO:CC	3.274E-28	31
GO:0005391	sodium:potassium-exchanging ATPase activity	GO:MF	1.54917E-21	31
GO:0010248	establishment or maintenance of transmembrane electrochemical gradient	GO:BP	4.88603E-19	31
GO:0030007	cellular potassium ion homeostasis	GO:BP	4.88603E-19	31
GO:0006883	cellular sodium ion homeostasis	GO:BP	7.78185E-18	31
KEGG:04974	Protein digestion and absorption	KEGG	3.76008E-16	31
KEGG:04964	Proximal tubule bicarbonate reclamation	KEGG	1.80345E-15	31
KEGG:04960	Aldosterone-regulated sodium reabsorption	KEGG	2.49235E-15	31
KEGG:04976	Bile secretion	KEGG	7.29832E-14	31
KEGG:04973	Carbohydrate digestion and absorption	KEGG	3.03212E-12	31
KEGG:04971	Gastric acid secretion	KEGG	4.81598E-12	31
KEGG:04978	Mineral absorption	KEGG	8.4653E-12	31
KEGG:04961	Endocrine and other factor-regulated calcium reabsorption	KEGG	4.15037E-11	31
KEGG:05168	Herpes simplex virus 1 infection	KEGG	1.22744E-35	33
GO:0046872	metal ion binding	GO:MF	2.23717E-33	33
GO:0005634	nucleus	GO:CC	1.04894E-19	33
GO:0003700	DNA-binding transcription factor activity	GO:MF	1.85789E-13	33

Supplementary Table S7. Enrichment Table for each separate community in MMU, ranked by the p-value

GO-Terms	*description*	*source*	*p-value*	*community*
KEGG:04080	Neuroactive ligand-receptor interaction	KEGG	1.1694E-151	0
GO:0007218	neuropeptide signaling pathway	GO:BP	1.51E-77	0
GO:0070098	chemokine-mediated signaling pathway	GO:BP	6.47311E-64	0
KEGG:04062	Chemokine signaling pathway	KEGG	5.04813E-57	0
KEGG:04742	Taste transduction	KEGG	4.36407E-41	0
GO:0008009	chemokine activity	GO:MF	9.48515E-41	0
KEGG:04061	Viral protein interaction with cytokine and cytokine receptor	KEGG	2.32747E-40	0
GO:0001580	detection of chemical stimulus involved in sensory perception of bitter taste	GO:BP	1.45288E-37	0
GO:0005834	heterotrimeric G-protein complex	GO:CC	7.21798E-36	0
GO:0016493	C-C chemokine receptor activity	GO:MF	1.02348E-33	0
GO:0019722	calcium-mediated signaling	GO:BP	1.26978E-33	0
GO:0070374	positive regulation of ERK1 and ERK2 cascade	GO:BP	1.09651E-31	0
KEGG:04725	Cholinergic synapse	KEGG	4.76009E-30	0
GO:0033038	bitter taste receptor activity	GO:MF	2.63685E-29	0
GO:0051482	positive regulation of cytosolic calcium ion concentration involved in phospholipase C-activating G protein-coupled signaling pathway	GO:BP	6.97399E-28	0
KEGG:04020	Calcium signaling pathway	KEGG	2.12107E-27	0
KEGG:04724	Glutamatergic synapse	KEGG	4.41027E-26	0
KEGG:04726	Serotonergic synapse	KEGG	3.16824E-24	0
GO:0042923	neuropeptide binding	GO:MF	4.04353E-21	0
GO:0031681	G-protein beta-subunit binding	GO:MF	9.2994E-20	0
GO:0031683	G-protein beta/gamma-subunit complex binding	GO:MF	4.13755E-19	0
KEGG:04060	Cytokine-cytokine receptor interaction	KEGG	8.95659E-19	0
GO:0045777	positive regulation of blood pressure	GO:BP	1.03941E-18	0
KEGG:04713	Circadian entrainment	KEGG	1.17819E-18	0
GO:0061844	antimicrobial humoral immune response mediated by antimicrobial peptide	GO:BP	1.61283E-17	0
KEGG:04072	Phospholipase D signaling pathway	KEGG	3.82586E-17	0
GO:0099056	integral component of presynaptic membrane	GO:CC	4.68531E-17	0
KEGG:05163	Human cytomegalovirus infection	KEGG	6.31757E-17	0
GO:0048245	eosinophil chemotaxis	GO:BP	7.14071E-17	0
KEGG:04723	Retrograde endocannabinoid signaling	KEGG	1.50852E-16	0
KEGG:04728	Dopaminergic synapse	KEGG	1.75829E-16	0
GO:0045907	positive regulation of vasoconstriction	GO:BP	4.43058E-16	0
GO:0004993	G protein-coupled serotonin receptor activity	GO:MF	4.4572E-16	0
KEGG:04926	Relaxin signaling pathway	KEGG	1.55076E-15	0
KEGG:04371	Apelin signaling pathway	KEGG	9.00417E-15	0
KEGG:05032	Morphine addiction	KEGG	1.50177E-14	0
GO:0031680	G-protein beta/gamma-subunit complex	GO:CC	3.69072E-14	0
GO:0005184	neuropeptide hormone activity	GO:MF	1.54817E-13	0
GO:0071347	cellular response to interleukin-1	GO:BP	7.80919E-13	0
KEGG:04929	GnRH secretion	KEGG	8.93822E-13	0
GO:0035815	positive regulation of renal sodium excretion	GO:BP	9.75909E-13	0
GO:0043547	positive regulation of GTPase activity	GO:BP	1.27572E-12	0
GO:0001965	G-protein alpha-subunit binding	GO:MF	1.48792E-12	0
KEGG:04024	cAMP signaling pathway	KEGG	1.77136E-12	0
GO:0042756	drinking behavior	GO:BP	8.51634E-12	0
GO:0007626	locomotory behavior	GO:BP	8.95956E-12	0
GO:0002430	complement receptor mediated signaling pathway	GO:BP	1.00672E-11	0
GO:0007189	adenylate cyclase-activating G protein-coupled receptor signaling pathway	GO:BP	1.02392E-11	0
GO:0051281	positive regulation of release of sequestered calcium ion into cytosol	GO:BP	1.22295E-11	0
KEGG:04924	Renin secretion	KEGG	2.18964E-11	0
KEGG:04022	cGMP-PKG signaling pathway	KEGG	5.23951E-11	0
GO:0007197	adenylate cyclase-inhibiting G protein-coupled acetylcholine receptor signaling pathway	GO:BP	8.63023E-11	0
GO:0051378	serotonin binding	GO:MF	8.80097E-11	0
KEGG:04080	Neuroactive ligand-receptor interaction	KEGG	1.04608E-65	1
GO:0043950	positive regulation of cAMP-mediated signaling	GO:BP	1.48659E-32	1
KEGG:04024	cAMP signaling pathway	KEGG	1.15311E-28	1
GO:0007190	activation of adenylate cyclase activity	GO:BP	1.13871E-24	1
KEGG:04911	Insulin secretion	KEGG	3.36954E-21	1
GO:0006171	cAMP biosynthetic process	GO:BP	2.18758E-19	1
KEGG:04923	Regulation of lipolysis in adipocytes	KEGG	6.08557E-17	1

KEGG:04270	Vascular smooth muscle contraction	KEGG	2.09513E-16	1
KEGG:04913	Ovarian steroidogenesis	KEGG	3.27024E-15	1
GO:0001594	trace-amine receptor activity	GO:MF	3.58277E-12	1
KEGG:04918	Thyroid hormone synthesis	KEGG	5.06896E-12	1
GO:0097647	amylin receptor signaling pathway	GO:BP	3.38385E-11	1
KEGG:04020	Calcium signaling pathway	KEGG	3.63507E-11	1
KEGG:04927	Cortisol synthesis and secretion	KEGG	6.53566E-11	1
KEGG:03460	Fanconi anemia pathway	KEGG	4.06375E-67	3
KEGG:03420	Nucleotide excision repair	KEGG	2.67824E-54	3
KEGG:03440	Homologous recombination	KEGG	2.85379E-46	3
GO:0003697	single-stranded DNA binding	GO:MF	2.38069E-42	3
KEGG:03030	DNA replication	KEGG	2.61672E-39	3
GO:0036297	interstrand cross-link repair	GO:BP	9.73021E-35	3
KEGG:03410	Base excision repair	KEGG	3.3106E-31	3
KEGG:03430	Mismatch repair	KEGG	2.20806E-27	3
GO:0003887	DNA-directed DNA polymerase activity	GO:MF	3.60402E-24	3
GO:0006303	double-strand break repair via nonhomologous end joining	GO:BP	3.7563E-24	3
GO:0003688	DNA replication origin binding	GO:MF	1.21208E-23	3
GO:0005524	ATP binding	GO:MF	6.52262E-22	3
GO:0017116	single-stranded DNA helicase activity	GO:MF	1.35959E-21	3
GO:0004843	thiol-dependent ubiquitin-specific protease activity	GO:MF	3.43574E-21	3
GO:0035861	site of double-strand break	GO:CC	3.81195E-19	3
GO:0006298	mismatch repair	GO:BP	6.54038E-19	3
GO:0000784	nuclear chromosome, telomeric region	GO:CC	1.80229E-18	3
GO:0043138	3'-5' DNA helicase activity	GO:MF	1.35347E-17	3
GO:0043240	Fanconi anaemia nuclear complex	GO:CC	1.42119E-17	3
GO:0030433	ubiquitin-dependent ERAD pathway	GO:BP	6.59872E-17	3
GO:0000727	double-strand break repair via break-induced replication	GO:BP	1.00813E-16	3
KEGG:03450	Non-homologous end-joining	KEGG	1.80602E-16	3
GO:0003682	chromatin binding	GO:MF	1.76521E-15	3
GO:0031297	replication fork processing	GO:BP	1.98484E-15	3
GO:0006268	DNA unwinding involved in DNA replication	GO:BP	7.61847E-15	3
GO:0042555	MCM complex	GO:CC	3.72005E-14	3
GO:0031573	intra-S DNA damage checkpoint	GO:BP	1.29171E-13	3
GO:0010165	response to X-ray	GO:BP	3.03588E-13	3
GO:0000400	four-way junction DNA binding	GO:MF	5.92224E-13	3
GO:0000712	resolution of meiotic recombination intermediates	GO:BP	1.13468E-12	3
GO:0031625	ubiquitin protein ligase binding	GO:MF	2.26024E-12	3
GO:0008821	crossover junction endodeoxyribonuclease activity	GO:MF	4.0902E-12	3
KEGG:04110	Cell cycle	KEGG	1.41849E-11	3
GO:0003689	DNA clamp loader activity	GO:MF	3.62477E-11	3
GO:0031011	Ino80 complex	GO:CC	6.45126E-11	3
GO:0043123	positive regulation of I-kappaB kinase/NF-kappaB signaling	GO:BP	3.9528E-55	4
KEGG:03050	Proteasome	KEGG	4.47968E-52	4
KEGG:05169	Epstein-Barr virus infection	KEGG	1.27018E-48	4
KEGG:05217	Basal cell carcinoma	KEGG	4.31177E-45	4
KEGG:04064	NF-kappa B signaling pathway	KEGG	4.1599E-44	4
KEGG:04310	Wnt signaling pathway	KEGG	1.37506E-38	4
GO:0051092	positive regulation of NF-kappaB transcription factor activity	GO:BP	5.26805E-33	4
KEGG:05200	Pathways in cancer	KEGG	3.69606E-31	4
KEGG:05226	Gastric cancer	KEGG	1.64002E-28	4
KEGG:05165	Human papillomavirus infection	KEGG	3.37634E-28	4
GO:0004298	threonine-type endopeptidase activity	GO:MF	3.98987E-27	4
GO:0005109	frizzled binding	GO:MF	4.4247E-27	4
KEGG:05224	Breast cancer	KEGG	1.75314E-26	4
KEGG:04622	RIG-I-like receptor signaling pathway	KEGG	1.21026E-25	4
GO:0010499	proteasomal ubiquitin-independent protein catabolic process	GO:BP	1.79726E-25	4
KEGG:04934	Cushing syndrome	KEGG	8.89915E-25	4
KEGG:04668	TNF signaling pathway	KEGG	2.33189E-24	4
KEGG:04620	Toll-like receptor signaling pathway	KEGG	3.24446E-24	4
KEGG:05162	Measles	KEGG	1.36258E-23	4
KEGG:05161	Hepatitis B	KEGG	2.2774E-23	4
KEGG:05225	Hepatocellular carcinoma	KEGG	7.2269E-23	4
KEGG:04110	Cell cycle	KEGG	2.42704E-21	4
KEGG:04390	Hippo signaling pathway	KEGG	1.55978E-20	4

GO:0004843	thiol-dependent ubiquitin-specific protease activity	GO:MF	9.47013E-20	4
KEGG:05160	Hepatitis C	KEGG	1.75642E-19	4
KEGG:04621	NOD-like receptor signaling pathway	KEGG	2.61605E-18	4
GO:0090090	negative regulation of canonical Wnt signaling pathway	GO:BP	1.37457E-17	4
KEGG:05164	Influenza A	KEGG	4.07995E-17	4
GO:0019901	protein kinase binding	GO:MF	6.66198E-17	4
GO:0005164	tumor necrosis factor receptor binding	GO:MF	1.27155E-16	4
KEGG:05222	Small cell lung cancer	KEGG	1.25518E-15	4
KEGG:04340	Hedgehog signaling pathway	KEGG	4.86201E-15	4
GO:0019774	proteasome core complex, beta-subunit complex	GO:CC	9.42032E-15	4
GO:0031625	ubiquitin protein ligase binding	GO:MF	5.69662E-14	4
KEGG:04150	mTOR signaling pathway	KEGG	1.59083E-13	4
KEGG:04218	Cellular senescence	KEGG	3.63199E-13	4
KEGG:05145	Toxoplasmosis	KEGG	3.66742E-13	4
GO:0035631	CD40 receptor complex	GO:CC	4.29271E-13	4
GO:0005149	interleukin-1 receptor binding	GO:MF	4.30255E-13	4
KEGG:04210	Apoptosis	KEGG	6.11191E-13	4
KEGG:04550	Signaling pathways regulating pluripotency of stem cells	KEGG	7.61297E-13	4
KEGG:05205	Proteoglycans in cancer	KEGG	1.05491E-12	4
KEGG:04657	IL-17 signaling pathway	KEGG	1.44007E-12	4
KEGG:04916	Melanogenesis	KEGG	1.49266E-12	4
GO:0042802	identical protein binding	GO:MF	2.33116E-12	4
GO:0008540	proteasome regulatory particle, base subcomplex	GO:CC	2.5365E-12	4
GO:0043507	positive regulation of JUN kinase activity	GO:BP	3.5958E-12	4
KEGG:05166	Human T-cell leukemia virus 1 infection	KEGG	1.36641E-11	4
GO:0071260	cellular response to mechanical stimulus	GO:BP	1.85583E-11	4
GO:0019773	proteasome core complex, alpha-subunit complex	GO:CC	1.95204E-11	4
GO:0005654	nucleoplasm	GO:CC	2.92073E-11	4
KEGG:04120	Ubiquitin mediated proteolysis	KEGG	3.094E-154	5
GO:0031146	SCF-dependent proteasomal ubiquitin-dependent protein catabolic process	GO:BP	1.89964E-44	5
GO:0061631	ubiquitin conjugating enzyme activity	GO:MF	2.54194E-39	5
GO:0070936	protein K48-linked ubiquitination	GO:BP	8.62247E-34	5
GO:0005680	anaphase-promoting complex	GO:CC	6.74193E-32	5
GO:0070979	protein K11-linked ubiquitination	GO:BP	1.29906E-29	5
GO:0051865	protein autoubiquitination	GO:BP	6.724E-22	5
GO:0031463	Cul3-RING ubiquitin ligase complex	GO:CC	7.39775E-22	5
GO:0080008	Cul4-RING E3 ubiquitin ligase complex	GO:CC	2.71905E-20	5
GO:0006513	protein monoubiquitination	GO:BP	1.56735E-19	5
GO:0031624	ubiquitin conjugating enzyme binding	GO:MF	1.49994E-17	5
GO:0045116	protein neddylation	GO:BP	1.34997E-16	5
GO:0031145	anaphase-promoting complex-dependent catabolic process	GO:BP	2.85323E-16	5
GO:0070534	protein K63-linked ubiquitination	GO:BP	4.72498E-16	5
GO:0097602	cullin family protein binding	GO:MF	1.555E-15	5
GO:0000338	protein deneddylation	GO:BP	1.60197E-15	5
GO:0031625	ubiquitin protein ligase binding	GO:MF	1.65891E-15	5
GO:0005829	cytosol	GO:CC	8.44774E-15	5
KEGG:04114	Oocyte meiosis	KEGG	4.12366E-13	5
GO:0032436	positive regulation of proteasomal ubiquitin-dependent protein catabolic process	GO:BP	1.16154E-12	5
KEGG:04110	Cell cycle	KEGG	2.15523E-11	5
GO:0031462	Cul2-RING ubiquitin ligase complex	GO:CC	9.22853E-11	5
GO:0005814	centriole	GO:CC	3.62278E-39	6
GO:0000139	Golgi membrane	GO:CC	4.65282E-30	6
KEGG:04145	Phagosome	KEGG	5.84038E-29	6
GO:0005829	cytosol	GO:CC	1.08326E-27	6
GO:0036064	ciliary basal body	GO:CC	8.43303E-27	6
GO:0030992	intraciliary transport particle B	GO:CC	5.84696E-25	6
GO:0006890	retrograde vesicle-mediated transport, Golgi to endoplasmic reticulum	GO:BP	3.50009E-24	6
KEGG:05323	Rheumatoid arthritis	KEGG	3.65796E-22	6
GO:0006891	intra-Golgi vesicle-mediated transport	GO:BP	1.69766E-21	6
GO:0005868	cytoplasmic dynein complex	GO:CC	5.30724E-21	6
KEGG:04966	Collecting duct acid secretion	KEGG	3.26507E-19	6
GO:0006606	protein import into nucleus	GO:BP	1.07736E-18	6
GO:1905515	non-motile cilium assembly	GO:BP	1.79831E-18	6
GO:0008574	ATP-dependent microtubule motor activity, plus-end-directed	GO:MF	1.26522E-17	6

KEGG:04114	Oocyte meiosis	KEGG	2.74289E-17	6
GO:0017056	structural constituent of nuclear pore	GO:MF	2.78962E-17	6
GO:0005484	SNAP receptor activity	GO:MF	3.32593E-17	6
KEGG:03013	RNA transport	KEGG	5.14086E-17	6
GO:0048471	perinuclear region of cytoplasm	GO:CC	9.37772E-17	6
GO:0031201	SNARE complex	GO:CC	1.71428E-15	6
KEGG:04141	Protein processing in endoplasmic reticulum	KEGG	1.85744E-15	6
KEGG:04130	SNARE interactions in vesicular transport	KEGG	1.98208E-15	6
GO:0036038	MKS complex	GO:CC	5.26944E-15	6
GO:0016471	vacuolar proton-transporting V-type ATPase complex	GO:CC	1.38007E-14	6
GO:0051082	unfolded protein binding	GO:MF	1.54638E-14	6
GO:0030127	COPII vesicle coat	GO:CC	2.01788E-14	6
GO:0051085	chaperone cofactor-dependent protein refolding	GO:BP	4.47429E-14	6
GO:0046961	proton-transporting ATPase activity, rotational mechanism	GO:MF	5.68398E-14	6
GO:0005801	cis-Golgi network	GO:CC	1.68174E-13	6
GO:0008536	Ran GTPase binding	GO:MF	4.07657E-13	6
GO:0030126	COPI vesicle coat	GO:CC	4.45379E-13	6
GO:0097431	mitotic spindle pole	GO:CC	5.19437E-13	6
GO:0000132	establishment of mitotic spindle orientation	GO:BP	3.88326E-12	6
GO:0005524	ATP binding	GO:MF	1.10408E-11	6
GO:0005200	structural constituent of cytoskeleton	GO:MF	1.41733E-11	6
KEGG:04721	Synaptic vesicle cycle	KEGG	2.05419E-11	6
GO:0030008	TRAPP complex	GO:CC	3.60976E-11	6
GO:0042613	MHC class II protein complex	GO:CC	3.60976E-11	6
GO:0051087	chaperone binding	GO:MF	5.27637E-11	6
KEGG:04962	Vasopressin-regulated water reabsorption	KEGG	5.93328E-11	6
KEGG:04610	Complement and coagulation cascades	KEGG	3.16091E-63	7
GO:0004252	serine-type endopeptidase activity	GO:MF	6.74752E-36	7
KEGG:00532	Glycosaminoglycan biosynthesis - chondroitin sulfate / dermatan sulfate	KEGG	1.01942E-27	7
KEGG:04512	ECM-receptor interaction	KEGG	1.69734E-27	7
KEGG:00534	Glycosaminoglycan biosynthesis - heparan sulfate / heparin	KEGG	6.39525E-26	7
KEGG:04510	Focal adhesion	KEGG	1.78391E-20	7
GO:0009986	cell surface	GO:CC	3.07314E-19	7
GO:0005509	calcium ion binding	GO:MF	1.00717E-18	7
KEGG:04151	PI3K-Akt signaling pathway	KEGG	1.83507E-17	7
GO:0008083	growth factor activity	GO:MF	2.22395E-16	7
GO:0005178	integrin binding	GO:MF	3.79414E-16	7
GO:0000139	Golgi membrane	GO:CC	6.5928E-16	7
GO:0008201	heparin binding	GO:MF	6.2338E-14	7
GO:0005796	Golgi lumen	GO:CC	1.79033E-13	7
GO:0030206	chondroitin sulfate biosynthetic process	GO:BP	1.88968E-13	7
GO:0004867	serine-type endopeptidase inhibitor activity	GO:MF	2.09105E-13	7
GO:0010951	negative regulation of endopeptidase activity	GO:BP	2.9588E-13	7
GO:0034361	very-low-density lipoprotein particle	GO:CC	3.68281E-13	7
GO:0042157	lipoprotein metabolic process	GO:BP	3.65673E-12	7
KEGG:04630	JAK-STAT signaling pathway	KEGG	1.07998E-76	8
KEGG:04360	Axon guidance	KEGG	1.41949E-62	8
KEGG:04810	Regulation of actin cytoskeleton	KEGG	2.67054E-61	8
KEGG:04014	Ras signaling pathway	KEGG	6.04629E-51	8
GO:0005096	GTPase activator activity	GO:MF	3.69843E-49	8
GO:0035023	regulation of Rho protein signal transduction	GO:BP	1.01309E-45	8
GO:0098978	glutamatergic synapse	GO:CC	2.40537E-44	8
KEGG:04015	Rap1 signaling pathway	KEGG	2.53734E-42	8
GO:0017124	SH3 domain binding	GO:MF	1.52213E-39	8
KEGG:04144	Endocytosis	KEGG	4.72336E-37	8
GO:0008360	regulation of cell shape	GO:BP	5.16487E-36	8
KEGG:04151	PI3K-Akt signaling pathway	KEGG	1.50869E-35	8
KEGG:05200	Pathways in cancer	KEGG	3.54436E-35	8
GO:0048013	ephrin receptor signaling pathway	GO:BP	2.46191E-34	8
KEGG:04010	MAPK signaling pathway	KEGG	9.69731E-34	8
KEGG:04060	Cytokine-cytokine receptor interaction	KEGG	3.77155E-33	8
KEGG:04650	Natural killer cell mediated cytotoxicity	KEGG	4.42502E-33	8
KEGG:04666	Fc gamma R-mediated phagocytosis	KEGG	7.75614E-33	8
KEGG:04012	ErbB signaling pathway	KEGG	3.18239E-32	8
GO:0005829	cytosol	GO:CC	1.54484E-31	8

KEGG:05100	Bacterial invasion of epithelial cells	KEGG	1.12971E-30	8
GO:0005925	focal adhesion	GO:CC	2.62279E-28	8
GO:0008083	growth factor activity	GO:MF	2.93484E-24	8
GO:0046875	ephrin receptor binding	GO:MF	2.45273E-23	8
GO:0005125	cytokine activity	GO:MF	7.57716E-23	8
KEGG:05205	Proteoglycans in cancer	KEGG	1.0735E-22	8
GO:0042531	positive regulation of tyrosine phosphorylation of STAT protein	GO:BP	1.85443E-22	8
KEGG:01521	EGFR tyrosine kinase inhibitor resistance	KEGG	5.23909E-22	8
KEGG:04722	Neurotrophin signaling pathway	KEGG	6.88264E-21	8
GO:0008543	fibroblast growth factor receptor signaling pathway	GO:BP	9.8939E-21	8
KEGG:04510	Focal adhesion	KEGG	6.27243E-20	8
KEGG:05135	Yersinia infection	KEGG	8.64606E-20	8
GO:0001784	phosphotyrosine residue binding	GO:MF	7.00116E-19	8
KEGG:04664	Fc epsilon RI signaling pathway	KEGG	1.27916E-18	8
GO:0004715	non-membrane spanning protein tyrosine kinase activity	GO:MF	1.5423E-18	8
GO:0045499	chemorepellent activity	GO:MF	2.14618E-17	8
KEGG:05218	Melanoma	KEGG	5.64028E-17	8
KEGG:04662	B cell receptor signaling pathway	KEGG	1.63237E-16	8
GO:0031901	early endosome membrane	GO:CC	2.51299E-16	8
GO:0004198	calcium-dependent cysteine-type endopeptidase activity	GO:MF	3.64711E-16	8
GO:0048471	perinuclear region of cytoplasm	GO:CC	7.35179E-16	8
GO:0005070	SH3/SH2 adaptor activity	GO:MF	1.08702E-15	8
KEGG:04660	T cell receptor signaling pathway	KEGG	1.88453E-15	8
GO:0050772	positive regulation of axonogenesis	GO:BP	4.55974E-15	8
GO:0001755	neural crest cell migration	GO:BP	5.84543E-15	8
GO:0038083	peptidyl-tyrosine autophosphorylation	GO:BP	5.85316E-15	8
GO:0030426	growth cone	GO:CC	9.65822E-15	8
KEGG:04072	Phospholipase D signaling pathway	KEGG	6.82377E-14	8
GO:0009897	external side of plasma membrane	GO:CC	8.84506E-14	8
GO:0098685	Schaffer collateral - CA1 synapse	GO:CC	1.4311E-13	8
KEGG:04062	Chemokine signaling pathway	KEGG	3.091E-13	8
GO:0048490	anterograde synaptic vesicle transport	GO:BP	3.99487E-13	8
GO:0005884	actin filament	GO:CC	4.37015E-13	8
GO:0042802	identical protein binding	GO:MF	5.01235E-13	8
KEGG:04659	Th17 cell differentiation	KEGG	8.92994E-13	8
GO:0042169	SH2 domain binding	GO:MF	1.18104E-12	8
KEGG:05226	Gastric cancer	KEGG	1.3373E-12	8
GO:0048268	clathrin coat assembly	GO:BP	1.43866E-12	8
KEGG:04640	Hematopoietic cell lineage	KEGG	2.72132E-12	8
GO:0016601	Rac protein signal transduction	GO:BP	3.16071E-12	8
GO:0048843	negative regulation of axon extension involved in axon guidance	GO:BP	3.18201E-12	8
KEGG:05224	Breast cancer	KEGG	4.97352E-12	8
GO:0005911	cell-cell junction	GO:CC	5.36643E-12	8
KEGG:04721	Synaptic vesicle cycle	KEGG	1.41714E-11	8
KEGG:05206	MicroRNAs in cancer	KEGG	1.58377E-11	8
KEGG:04520	Adherens junction	KEGG	1.86798E-11	8
GO:0005885	Arp2/3 protein complex	GO:CC	4.14516E-11	8
GO:0030336	negative regulation of cell migration	GO:BP	4.49419E-11	8
GO:0043524	negative regulation of neuron apoptotic process	GO:BP	5.02693E-11	8
KEGG:05321	Inflammatory bowel disease (IBD)	KEGG	5.93308E-11	8
GO:0005005	transmembrane-ephrin receptor activity	GO:MF	6.73878E-11	8
GO:0014068	positive regulation of phosphatidylinositol 3-kinase signaling	GO:BP	7.11338E-11	8
KEGG:00190	Oxidative phosphorylation	KEGG	1.0733E-158	9
KEGG:04714	Thermogenesis	KEGG	1.2758E-144	9
KEGG:05012	Parkinson disease	KEGG	1.4315E-140	9
KEGG:05010	Alzheimer disease	KEGG	5.8955E-113	9
KEGG:05016	Huntington disease	KEGG	3.7753E-108	9
KEGG:04932	Non-alcoholic fatty liver disease (NAFLD)	KEGG	1.07598E-92	9
GO:0005747	mitochondrial respiratory chain complex I	GO:CC	6.81037E-83	9
GO:0032981	mitochondrial respiratory chain complex I assembly	GO:BP	5.6372E-67	9
KEGG:01100	Metabolic pathways	KEGG	2.93588E-54	9
GO:0008137	NADH dehydrogenase (ubiquinone) activity	GO:MF	8.89785E-50	9
KEGG:04723	Retrograde endocannabinoid signaling	KEGG	5.36584E-46	9
GO:0004129	cytochrome-c oxidase activity	GO:MF	1.03103E-37	9
KEGG:04260	Cardiac muscle contraction	KEGG	1.03193E-29	9

GO:0006120	mitochondrial electron transport, NADH to ubiquinone	GO:BP	3.65312E-22	9
GO:0000276	mitochondrial proton-transporting ATP synthase complex, coupling factor F(o)	GO:CC	3.79348E-20	9
GO:0046933	proton-transporting ATP synthase activity, rotational mechanism	GO:MF	5.55023E-19	9
GO:0005751	mitochondrial respiratory chain complex IV	GO:CC	1.13322E-16	9
GO:0051537	2 iron, 2 sulfur cluster binding	GO:MF	4.88689E-16	9
KEGG:00860	Porphyrin and chlorophyll metabolism	KEGG	2.93099E-14	9
GO:0051539	4 iron, 4 sulfur cluster binding	GO:MF	1.43684E-13	9
GO:0097428	protein maturation by iron-sulfur cluster transfer	GO:BP	6.2627E-13	9
GO:0005750	mitochondrial respiratory chain complex III	GO:CC	1.03863E-12	9
GO:0006123	mitochondrial electron transport, cytochrome c to oxygen	GO:BP	1.94527E-11	9
KEGG:01100	Metabolic pathways	KEGG	2.1031E-127	10
KEGG:01200	Carbon metabolism	KEGG	2.32431E-74	10
KEGG:01230	Biosynthesis of amino acids	KEGG	7.12848E-62	10
KEGG:00520	Amino sugar and nucleotide sugar metabolism	KEGG	1.03717E-43	10
KEGG:00010	Glycolysis / Gluconeogenesis	KEGG	3.45294E-41	10
KEGG:00020	Citrate cycle (TCA cycle)	KEGG	1.91365E-31	10
KEGG:00030	Pentose phosphate pathway	KEGG	3.69414E-30	10
GO:0006099	tricarboxylic acid cycle	GO:BP	2.35814E-29	10
KEGG:00052	Galactose metabolism	KEGG	4.19773E-28	10
KEGG:00051	Fructose and mannose metabolism	KEGG	2.31084E-27	10
KEGG:01210	2-Oxocarboxylic acid metabolism	KEGG	7.20378E-24	10
KEGG:00250	Alanine, aspartate and glutamate metabolism	KEGG	7.34121E-23	10
KEGG:00220	Arginine biosynthesis	KEGG	2.82275E-21	10
GO:0004364	glutathione transferase activity	GO:MF	2.22827E-18	10
GO:0051287	NAD binding	GO:MF	5.70857E-18	10
GO:0046835	carbohydrate phosphorylation	GO:BP	1.26235E-17	10
KEGG:00531	Glycosaminoglycan degradation	KEGG	1.67224E-17	10
KEGG:00500	Starch and sucrose metabolism	KEGG	1.72077E-17	10
GO:0006002	fructose 6-phosphate metabolic process	GO:BP	6.70684E-17	10
KEGG:00620	Pyruvate metabolism	KEGG	2.06076E-16	10
KEGG:05230	Central carbon metabolism in cancer	KEGG	8.52358E-16	10
GO:0006749	glutathione metabolic process	GO:BP	1.4964E-15	10
GO:0006103	2-oxoglutarate metabolic process	GO:BP	1.06258E-14	10
GO:0042803	protein homodimerization activity	GO:MF	4.02538E-14	10
KEGG:00380	Tryptophan metabolism	KEGG	5.99802E-14	10
GO:0005615	extracellular space	GO:CC	1.82512E-13	10
KEGG:00270	Cysteine and methionine metabolism	KEGG	2.81159E-13	10
KEGG:00480	Glutathione metabolism	KEGG	3.36252E-13	10
KEGG:04066	HIF-1 signaling pathway	KEGG	1.84032E-12	10
KEGG:00350	Tyrosine metabolism	KEGG	2.12536E-12	10
GO:0043209	myelin sheath	GO:CC	3.41447E-12	10
KEGG:04142	Lysosome	KEGG	6.39275E-12	10
GO:0030388	fructose 1,6-bisphosphate metabolic process	GO:BP	1.95471E-11	10
GO:0006000	fructose metabolic process	GO:BP	8.27683E-11	10
KEGG:00562	Inositol phosphate metabolism	KEGG	6.20871E-67	11
KEGG:04070	Phosphatidylinositol signaling system	KEGG	2.37421E-53	11
GO:0098978	glutamatergic synapse	GO:CC	6.28843E-38	11
GO:0032281	AMPA glutamate receptor complex	GO:CC	2.74506E-32	11
GO:0005516	calmodulin binding	GO:MF	5.0898E-24	11
GO:0030054	cell junction	GO:CC	4.07266E-23	11
GO:0046854	phosphatidylinositol phosphorylation	GO:BP	4.28697E-22	11
KEGG:04020	Calcium signaling pathway	KEGG	2.18695E-21	11
GO:0001518	voltage-gated sodium channel complex	GO:CC	1.48734E-20	11
GO:0099061	integral component of postsynaptic density membrane	GO:CC	2.21343E-20	11
GO:0035235	ionotropic glutamate receptor signaling pathway	GO:BP	3.92992E-19	11
GO:0006182	cGMP biosynthetic process	GO:BP	1.50152E-18	11
KEGG:04921	Oxytocin signaling pathway	KEGG	1.50356E-18	11
GO:0017146	NMDA selective glutamate receptor complex	GO:CC	2.47556E-18	11
GO:0008331	high voltage-gated calcium channel activity	GO:MF	2.33057E-17	11
GO:0007168	receptor guanylyl cyclase signaling pathway	GO:BP	8.05375E-15	11
GO:0004438	phosphatidylinositol-3-phosphatase activity	GO:MF	1.9632E-14	11
GO:0004383	guanylate cyclase activity	GO:MF	1.06835E-13	11
GO:0046855	inositol phosphate dephosphorylation	GO:BP	1.19575E-13	11
KEGG:04260	Cardiac muscle contraction	KEGG	1.29619E-13	11
KEGG:04261	Adrenergic signaling in cardiomyocytes	KEGG	1.65764E-13	11

KEGG:04713	Circadian entrainment	KEGG	2.63648E-13	11
KEGG:04724	Glutamatergic synapse	KEGG	6.15453E-13	11
GO:0060076	excitatory synapse	GO:CC	1.13133E-12	11
GO:0019228	neuronal action potential	GO:BP	3.64131E-12	11
GO:0098919	structural constituent of postsynaptic density	GO:MF	7.41366E-12	11
GO:0016941	natriuretic peptide receptor activity	GO:MF	1.42116E-11	11
GO:0016308	1-phosphatidylinositol-4-phosphate 5-kinase activity	GO:MF	1.42116E-11	11
KEGG:04911	Insulin secretion	KEGG	3.28673E-11	11
KEGG:04022	cGMP-PKG signaling pathway	KEGG	4.87749E-11	11
KEGG:05412	Arrhythmogenic right ventricular cardiomyopathy (ARVC)	KEGG	5.57552E-11	11
KEGG:05031	Amphetamine addiction	KEGG	6.66078E-11	11
GO:0030314	junctional membrane complex	GO:CC	9.09328E-11	11
GO:0033017	sarcoplasmic reticulum membrane	GO:CC	9.61372E-11	11
KEGG:04140	Autophagy - animal	KEGG	5.13947E-51	12
GO:0005829	cytosol	GO:CC	1.47135E-41	12
KEGG:04150	mTOR signaling pathway	KEGG	7.40498E-36	12
KEGG:04136	Autophagy - other	KEGG	3.44823E-34	12
KEGG:04350	TGF-beta signaling pathway	KEGG	1.31271E-29	12
KEGG:04390	Hippo signaling pathway	KEGG	2.54739E-28	12
KEGG:04211	Longevity regulating pathway	KEGG	1.04566E-20	12
GO:0010862	positive regulation of pathway-restricted SMAD protein phosphorylation	GO:BP	3.74101E-20	12
KEGG:04392	Hippo signaling pathway - multiple species	KEGG	4.85039E-19	12
GO:0035329	hippo signaling	GO:BP	4.24632E-18	12
KEGG:04137	Mitophagy - animal	KEGG	5.42541E-18	12
GO:0000421	autophagosome membrane	GO:CC	1.61897E-17	12
GO:0030057	desmosome	GO:CC	4.6335E-17	12
GO:0031932	TORC2 complex	GO:CC	5.38975E-17	12
GO:0061952	midbody abscission	GO:BP	7.94381E-17	12
GO:1904262	negative regulation of TORC1 signaling	GO:BP	4.36699E-14	12
KEGG:04152	AMPK signaling pathway	KEGG	4.69541E-14	12
GO:0005923	bicellular tight junction	GO:CC	8.82915E-14	12
KEGG:04530	Tight junction	KEGG	1.19238E-13	12
GO:0042149	cellular response to glucose starvation	GO:BP	2.12413E-13	12
GO:0043162	ubiquitin-dependent protein catabolic process via the multivesicular body sorting pathway	GO:BP	3.42986E-13	12
KEGG:04068	FoxO signaling pathway	KEGG	9.90497E-13	12
GO:0008285	negative regulation of cell population proliferation	GO:BP	1.02327E-12	12
GO:0048185	activin binding	GO:MF	1.17848E-12	12
GO:0034045	phagophore assembly site membrane	GO:CC	1.51418E-12	12
GO:0042803	protein homodimerization activity	GO:MF	1.99144E-12	12
GO:0045324	late endosome to vacuole transport	GO:BP	1.11511E-11	12
KEGG:04213	Longevity regulating pathway - multiple species	KEGG	2.29269E-11	12
GO:0000815	ESCRT III complex	GO:CC	6.57491E-11	12
GO:0000122	negative regulation of transcription by RNA polymerase II	GO:BP	1.8457E-119	13
GO:0001228	DNA-binding transcription activator activity, RNA polymerase II-specific	GO:MF	5.20895E-58	13
GO:0003714	transcription corepressor activity	GO:MF	1.81157E-43	13
GO:0016592	mediator complex	GO:CC	2.45735E-43	13
GO:0042826	histone deacetylase binding	GO:MF	1.47418E-32	13
KEGG:04330	Notch signaling pathway	KEGG	3.60533E-30	13
GO:0008270	zinc ion binding	GO:MF	1.37803E-29	13
GO:0003707	steroid hormone receptor activity	GO:MF	6.9789E-29	13
GO:0046982	protein heterodimerization activity	GO:MF	3.07951E-24	13
GO:0005719	nuclear euchromatin	GO:CC	6.16523E-24	13
KEGG:05034	Alcoholism	KEGG	8.90915E-24	13
GO:0035267	NuA4 histone acetyltransferase complex	GO:CC	1.46728E-22	13
KEGG:05202	Transcriptional misregulation in cancer	KEGG	5.32188E-22	13
GO:0035064	methylated histone binding	GO:MF	1.36316E-20	13
GO:0035102	PRC1 complex	GO:CC	2.32359E-20	13
GO:0000980	RNA polymerase II distal enhancer sequence-specific DNA binding	GO:MF	3.75161E-20	13
GO:0016581	NuRD complex	GO:CC	3.60314E-19	13
KEGG:04919	Thyroid hormone signaling pathway	KEGG	5.90232E-19	13
GO:0016514	SWI/SNF complex	GO:CC	6.73773E-19	13
GO:0071565	nBAF complex	GO:CC	7.05718E-19	13
GO:1990841	promoter-specific chromatin binding	GO:MF	6.87351E-18	13
GO:0035914	skeletal muscle cell differentiation	GO:BP	1.35963E-17	13

GO:0001227	DNA-binding transcription repressor activity, RNA polymerase II-specific	GO:MF	1.77034E-17	13
GO:0043968	histone H2A acetylation	GO:BP	2.00242E-17	13
GO:0042800	histone methyltransferase activity (H3-K4 specific)	GO:MF	2.70255E-17	13
GO:0004879	nuclear receptor activity	GO:MF	5.28162E-17	13
GO:0035098	ESC/E(Z) complex	GO:CC	7.95616E-17	13
GO:0035019	somatic stem cell population maintenance	GO:BP	2.2987E-16	13
GO:0001102	RNA polymerase II activating transcription factor binding	GO:MF	2.59098E-16	13
GO:0006337	nucleosome disassembly	GO:BP	5.37332E-16	13
GO:0043982	histone H4-K8 acetylation	GO:BP	5.37332E-16	13
GO:0043981	histone H4-K5 acetylation	GO:BP	5.37332E-16	13
GO:0008285	negative regulation of cell population proliferation	GO:BP	9.13407E-16	13
GO:0008013	beta-catenin binding	GO:MF	9.35132E-16	13
KEGG:05322	Systemic lupus erythematosus	KEGG	4.03273E-15	13
GO:0016580	Sin3 complex	GO:CC	8.17106E-15	13
GO:0071564	npBAF complex	GO:CC	1.96088E-14	13
GO:0043984	histone H4-K16 acetylation	GO:BP	2.14766E-14	13
GO:0016342	catenin complex	GO:CC	1.27236E-13	13
GO:0071339	MLL1 complex	GO:CC	2.25411E-12	13
KEGG:05203	Viral carcinogenesis	KEGG	4.47553E-12	13
GO:0003151	outflow tract morphogenesis	GO:BP	6.0726E-12	13
GO:1990907	beta-catenin-TCF complex	GO:CC	6.319E-12	13
GO:0016607	nuclear speck	GO:CC	1.45691E-11	13
KEGG:00310	Lysine degradation	KEGG	2.21301E-11	13
GO:0001085	RNA polymerase II transcription factor binding	GO:MF	3.3572E-11	13
GO:0008584	male gonad development	GO:BP	4.07055E-11	13
GO:0032482	Rab protein signal transduction	GO:BP	2.26366E-54	14
GO:0008076	voltage-gated potassium channel complex	GO:CC	4.07326E-49	14
KEGG:04514	Cell adhesion molecules (CAMs)	KEGG	1.76891E-40	14
GO:0005251	delayed rectifier potassium channel activity	GO:MF	3.5572E-40	14
KEGG:04145	Phagosome	KEGG	1.07118E-33	14
GO:0009897	external side of plasma membrane	GO:CC	3.32473E-32	14
GO:0003924	GTPase activity	GO:MF	2.43234E-27	14
GO:0030670	phagocytic vesicle membrane	GO:CC	3.47362E-24	14
GO:0005525	GTP binding	GO:MF	1.62971E-23	14
GO:0042605	peptide antigen binding	GO:MF	2.80517E-22	14
GO:0005892	acetylcholine-gated channel complex	GO:CC	4.04069E-22	14
KEGG:04612	Antigen processing and presentation	KEGG	1.11323E-21	14
GO:0022848	acetylcholine-gated cation-selective channel activity	GO:MF	5.69632E-21	14
GO:0002476	antigen processing and presentation of endogenous peptide antigen via MHC class Ib	GO:BP	1.08901E-20	14
GO:0001916	positive regulation of T cell mediated cytotoxicity	GO:BP	5.02223E-20	14
GO:0002486	antigen processing and presentation of endogenous peptide antigen via MHC class I via ER pathway, TAP-independent	GO:BP	2.70969E-19	14
KEGG:05332	Graft-versus-host disease	KEGG	6.06442E-19	14
GO:0006886	intracellular protein transport	GO:BP	1.8273E-18	14
GO:0045121	membrane raft	GO:CC	4.07633E-18	14
GO:0019003	GDP binding	GO:MF	1.09991E-17	14
GO:0007229	integrin-mediated signaling pathway	GO:BP	2.72593E-17	14
GO:0042166	acetylcholine binding	GO:MF	6.19982E-17	14
GO:0008305	integrin complex	GO:CC	1.00803E-16	14
GO:0051260	protein homooligomerization	GO:BP	2.04054E-16	14
KEGG:05416	Viral myocarditis	KEGG	7.71099E-15	14
GO:0007160	cell-matrix adhesion	GO:BP	1.43136E-14	14
KEGG:05330	Allograft rejection	KEGG	3.86825E-14	14
GO:0098993	anchored component of synaptic vesicle membrane	GO:CC	4.17035E-14	14
GO:0015464	acetylcholine receptor activity	GO:MF	1.0478E-13	14
GO:0017112	Rab guanyl-nucleotide exchange factor activity	GO:MF	1.18263E-13	14
KEGG:04940	Type I diabetes mellitus	KEGG	6.0253E-13	14
GO:0062061	TAP complex binding	GO:MF	1.73885E-12	14
GO:0060079	excitatory postsynaptic potential	GO:BP	3.69826E-12	14
KEGG:05320	Autoimmune thyroid disease	KEGG	6.36752E-12	14
KEGG:05170	Human immunodeficiency virus 1 infection	KEGG	2.06182E-11	14
GO:0007271	synaptic transmission, cholinergic	GO:BP	5.30237E-11	14
GO:0042612	MHC class I protein complex	GO:CC	8.0623E-11	14
GO:0030881	beta-2-microglobulin binding	GO:MF	9.14682E-11	14

GO:0042610	CD8 receptor binding	GO:MF	9.14682E-11	14
GO:0003735	structural constituent of ribosome	GO:MF	1.3222E-129	15
KEGG:03010	Ribosome	KEGG	1.1255E-109	15
GO:0022625	cytosolic large ribosomal subunit	GO:CC	1.66148E-89	15
GO:0003743	translation initiation factor activity	GO:MF	1.16184E-77	15
GO:0022627	cytosolic small ribosomal subunit	GO:CC	2.49863E-66	15
GO:0042788	polysomal ribosome	GO:CC	2.77354E-39	15
KEGG:03013	RNA transport	KEGG	5.70043E-37	15
GO:0001732	formation of cytoplasmic translation initiation complex	GO:BP	7.31361E-29	15
GO:0016282	eukaryotic 43S preinitiation complex	GO:CC	1.81134E-28	15
GO:0033290	eukaryotic 48S preinitiation complex	GO:CC	1.04767E-27	15
GO:0000184	nuclear-transcribed mRNA catabolic process, nonsense-mediated decay	GO:BP	9.99736E-21	15
GO:0043022	ribosome binding	GO:MF	2.68754E-17	15
GO:0001731	formation of translation preinitiation complex	GO:BP	5.04274E-17	15
GO:0000028	ribosomal small subunit assembly	GO:BP	6.32705E-15	15
GO:0071541	eukaryotic translation initiation factor 3 complex, eIF3m	GO:CC	7.67282E-14	15
KEGG:03015	mRNA surveillance pathway	KEGG	6.40058E-13	15
GO:0031369	translation initiation factor binding	GO:MF	1.33208E-12	15
GO:0008250	oligosaccharyltransferase complex	GO:CC	1.23058E-11	15
GO:0000027	ribosomal large subunit assembly	GO:BP	1.57132E-11	15
GO:0006446	regulation of translational initiation	GO:BP	9.80757E-11	15
KEGG:03040	Spliceosome	KEGG	5.2859E-170	16
GO:0016607	nuclear speck	GO:CC	8.76266E-91	16
GO:0071005	U2-type precatalytic spliceosome	GO:CC	1.96064E-68	16
GO:0071007	U2-type catalytic step 2 spliceosome	GO:CC	1.64065E-42	16
KEGG:03015	mRNA surveillance pathway	KEGG	2.05276E-42	16
GO:0005689	U12-type spliceosomal complex	GO:CC	3.03779E-39	16
GO:0046540	U4/U6 x U5 tri-snRNP complex	GO:CC	4.08691E-36	16
KEGG:03022	Basal transcription factors	KEGG	7.56268E-34	16
GO:0005669	transcription factor TFIID complex	GO:CC	2.4552E-31	16
GO:0005686	U2 snRNP	GO:CC	1.16496E-27	16
GO:0000381	regulation of alternative mRNA splicing, via spliceosome	GO:BP	6.66685E-27	16
GO:0005666	RNA polymerase III complex	GO:CC	5.72796E-26	16
GO:0005685	U1 snRNP	GO:CC	1.67814E-25	16
GO:0034719	SMN-Sm protein complex	GO:CC	1.67814E-25	16
KEGG:03020	RNA polymerase	KEGG	1.91397E-25	16
KEGG:03013	RNA transport	KEGG	1.64902E-24	16
GO:0001056	RNA polymerase III activity	GO:MF	1.19241E-22	16
GO:0005682	U5 snRNP	GO:CC	6.57842E-21	16
GO:0015030	Cajal body	GO:CC	4.78117E-20	16
GO:0045292	mRNA cis splicing, via spliceosome	GO:BP	1.45324E-19	16
GO:0071004	U2-type prespliceosome	GO:CC	1.17755E-18	16
GO:0005847	mRNA cleavage and polyadenylation specificity factor complex	GO:CC	1.79593E-16	16
GO:0000974	Prp19 complex	GO:CC	5.07374E-16	16
GO:0048025	negative regulation of mRNA splicing, via spliceosome	GO:BP	8.01165E-16	16
GO:0005687	U4 snRNP	GO:CC	3.07526E-15	16
GO:0005665	RNA polymerase II, core complex	GO:CC	4.53247E-15	16
GO:0032968	positive regulation of transcription elongation from RNA polymerase II promoter	GO:BP	1.02276E-14	16
GO:0000993	RNA polymerase II complex binding	GO:MF	4.81741E-14	16
GO:0033276	transcription factor TFTC complex	GO:CC	7.65021E-14	16
GO:1990446	U1 snRNP binding	GO:MF	1.70251E-13	16
GO:0000124	SAGA complex	GO:CC	8.33367E-13	16
GO:0071006	U2-type catalytic step 1 spliceosome	GO:CC	8.33367E-13	16
GO:0016251	RNA polymerase II general transcription initiation factor activity	GO:MF	8.75725E-13	16
GO:0032797	SMN complex	GO:CC	1.53834E-12	16
GO:0032039	integrator complex	GO:CC	1.68947E-12	16
GO:0036396	RNA N6-methyladenosine methyltransferase complex	GO:CC	7.95599E-12	16
GO:0004402	histone acetyltransferase activity	GO:MF	2.26079E-11	16
GO:0098789	pre-mRNA cleavage required for polyadenylation	GO:BP	5.5435E-11	16
GO:0001055	RNA polymerase II activity	GO:MF	7.93202E-11	16
KEGG:00601	Glycosphingolipid biosynthesis - lacto and neolacto series	KEGG	1.92445E-48	17
KEGG:00512	Mucin type O-glycan biosynthesis	KEGG	1.78461E-47	17
KEGG:01100	Metabolic pathways	KEGG	4.22535E-42	17
GO:0032580	Golgi cisterna membrane	GO:CC	1.1987E-30	17

GO:0004653	polypeptide N-acetylgalactosaminyltransferase activity	GO:MF	1.19924E-27	17
GO:0016021	integral component of membrane	GO:CC	2.17573E-26	17
KEGG:00603	Glycosphingolipid biosynthesis - globo and isoglobo series	KEGG	1.03623E-22	17
KEGG:00533	Glycosaminoglycan biosynthesis - keratan sulfate	KEGG	2.0572E-21	17
KEGG:00604	Glycosphingolipid biosynthesis - ganglio series	KEGG	1.02077E-20	17
GO:0030311	poly-N-acetyllactosamine biosynthetic process	GO:BP	1.64201E-17	17
GO:0008532	N-acetyllactosaminide beta-1,3-N-acetylglucosaminyltransferase activity	GO:MF	7.04575E-16	17
GO:0009312	oligosaccharide biosynthetic process	GO:BP	3.23735E-15	17
GO:0030259	lipid glycosylation	GO:BP	2.40569E-12	17
KEGG:00510	N-Glycan biosynthesis	KEGG	3.6208E-11	17
KEGG:01100	Metabolic pathways	KEGG	2.3168E-201	19
KEGG:00830	Retinol metabolism	KEGG	9.17267E-92	19
GO:0005506	iron ion binding	GO:MF	2.28452E-87	19
KEGG:00140	Steroid hormone biosynthesis	KEGG	3.55263E-76	19
GO:0020037	heme binding	GO:MF	5.18289E-76	19
KEGG:04146	Peroxisome	KEGG	1.35413E-60	19
KEGG:00590	Arachidonic acid metabolism	KEGG	1.10795E-55	19
KEGG:00071	Fatty acid degradation	KEGG	5.7284E-55	19
KEGG:01212	Fatty acid metabolism	KEGG	9.57746E-52	19
KEGG:05204	Chemical carcinogenesis	KEGG	1.13255E-50	19
GO:0070330	aromatase activity	GO:MF	3.99596E-43	19
KEGG:00280	Valine, leucine and isoleucine degradation	KEGG	1.4096E-42	19
GO:0042738	exogenous drug catabolic process	GO:BP	2.5411E-37	19
KEGG:00410	beta-Alanine metabolism	KEGG	3.82289E-33	19
GO:0008392	arachidonic acid epoxygenase activity	GO:MF	5.897E-33	19
KEGG:00650	Butanoate metabolism	KEGG	1.7445E-30	19
KEGG:00980	Metabolism of xenobiotics by cytochrome P450	KEGG	8.4702E-29	19
GO:0019373	epoxygenase P450 pathway	GO:BP	6.87624E-28	19
KEGG:00260	Glycine, serine and threonine metabolism	KEGG	5.08218E-26	19
GO:0042572	retinol metabolic process	GO:BP	3.50718E-24	19
GO:0004745	retinol dehydrogenase activity	GO:MF	5.83583E-24	19
KEGG:00061	Fatty acid biosynthesis	KEGG	6.86238E-23	19
KEGG:00982	Drug metabolism - cytochrome P450	KEGG	8.28631E-23	19
KEGG:00340	Histidine metabolism	KEGG	5.4345E-21	19
KEGG:00062	Fatty acid elongation	KEGG	1.05297E-20	19
KEGG:00591	Linoleic acid metabolism	KEGG	1.17542E-19	19
GO:0005782	peroxisomal matrix	GO:CC	1.53703E-19	19
KEGG:03320	PPAR signaling pathway	KEGG	3.54513E-19	19
KEGG:00120	Primary bile acid biosynthesis	KEGG	4.79717E-19	19
KEGG:00053	Ascorbate and aldarate metabolism	KEGG	2.22441E-18	19
KEGG:00670	One carbon pool by folate	KEGG	3.88539E-18	19
KEGG:01040	Biosynthesis of unsaturated fatty acids	KEGG	1.37057E-17	19
GO:0071949	FAD binding	GO:MF	3.48142E-17	19
KEGG:00640	Propanoate metabolism	KEGG	3.58256E-17	19
GO:0008401	retinoic acid 4-hydroxylase activity	GO:MF	9.4835E-17	19
GO:0003996	acyl-CoA ligase activity	GO:MF	9.4835E-17	19
GO:0051287	NAD binding	GO:MF	1.0396E-16	19
GO:0006695	cholesterol biosynthetic process	GO:BP	2.13654E-16	19
GO:0035999	tetrahydrofolate interconversion	GO:BP	3.43176E-16	19
KEGG:00900	Terpenoid backbone biosynthesis	KEGG	5.14004E-16	19
GO:0033539	fatty acid beta-oxidation using acyl-CoA dehydrogenase	GO:BP	1.25186E-15	19
KEGG:00630	Glyoxylate and dicarboxylate metabolism	KEGG	2.14561E-15	19
KEGG:00380	Tryptophan metabolism	KEGG	2.37498E-15	19
GO:0101020	estrogen 16-alpha-hydroxylase activity	GO:MF	3.93262E-15	19
GO:0051289	protein homotetramerization	GO:BP	2.08035E-14	19
GO:0030170	pyridoxal phosphate binding	GO:MF	3.94949E-14	19
GO:0000062	fatty-acyl-CoA binding	GO:MF	5.6102E-14	19
GO:0035338	long-chain fatty-acyl-CoA biosynthetic process	GO:BP	5.80649E-14	19
GO:0015020	glucuronosyltransferase activity	GO:MF	1.73505E-13	19
KEGG:01200	Carbon metabolism	KEGG	2.75573E-13	19
KEGG:00480	Glutathione metabolism	KEGG	3.62488E-13	19
GO:0042803	protein homodimerization activity	GO:MF	5.16502E-13	19
KEGG:00350	Tyrosine metabolism	KEGG	9.25262E-13	19
KEGG:00072	Synthesis and degradation of ketone bodies	KEGG	1.17356E-12	19
KEGG:00100	Steroid biosynthesis	KEGG	1.56804E-12	19

KEGG:04913	Ovarian steroidogenesis	KEGG	3.67174E-12	19
GO:0102391	decanoate-CoA ligase activity	GO:MF	6.57334E-12	19
KEGG:04726	Serotonergic synapse	KEGG	2.21604E-11	19
GO:0003857	3-hydroxyacyl-CoA dehydrogenase activity	GO:MF	2.99463E-11	19
KEGG:00230	Purine metabolism	KEGG	2.7605E-100	20
KEGG:00240	Pyrimidine metabolism	KEGG	8.85226E-73	20
KEGG:01100	Metabolic pathways	KEGG	4.38655E-69	20
KEGG:00760	Nicotinate and nicotinamide metabolism	KEGG	2.91517E-35	20
GO:0004550	nucleoside diphosphate kinase activity	GO:MF	1.01148E-28	20
KEGG:00983	Drug metabolism - other enzymes	KEGG	3.06396E-20	20
GO:0005524	ATP binding	GO:MF	1.95226E-19	20
GO:0046940	nucleoside monophosphate phosphorylation	GO:BP	3.40244E-19	20
GO:0006165	nucleoside diphosphate phosphorylation	GO:BP	5.40929E-15	20
GO:0004115	3',5'-cyclic-AMP phosphodiesterase activity	GO:MF	2.17394E-14	20
GO:0008253	5'-nucleotidase activity	GO:MF	2.17394E-14	20
KEGG:00730	Thiamine metabolism	KEGG	3.18128E-13	20
KEGG:00740	Riboflavin metabolism	KEGG	4.54761E-13	20
GO:0006183	GTP biosynthetic process	GO:BP	2.14529E-12	20
KEGG:00770	Pantothenate and CoA biosynthesis	KEGG	4.44066E-12	20
GO:0004017	adenylate kinase activity	GO:MF	5.01205E-12	20
GO:0006198	cAMP catabolic process	GO:BP	6.70203E-11	20
GO:0030150	protein import into mitochondrial matrix	GO:BP	4.41987E-24	21
GO:0005742	mitochondrial outer membrane translocase complex	GO:CC	1.98684E-21	21
KEGG:03008	Ribosome biogenesis in eukaryotes	KEGG	3.78769E-98	22
GO:0032040	small-subunit processome	GO:CC	2.52144E-51	22
GO:0030687	preribosome, large subunit precursor	GO:CC	7.01709E-35	22
GO:0000176	nuclear exosome (RNase complex)	GO:CC	1.59014E-22	22
KEGG:03018	RNA degradation	KEGG	9.84716E-22	22
GO:0001682	tRNA 5'-leader removal	GO:BP	9.6304E-19	22
GO:0033204	ribonuclease P RNA binding	GO:MF	1.40756E-18	22
GO:0003724	RNA helicase activity	GO:MF	1.83553E-18	22
GO:0004526	ribonuclease P activity	GO:MF	1.53658E-17	22
GO:0034427	nuclear-transcribed mRNA catabolic process, exonucleolytic, 3'-5'	GO:BP	9.83391E-15	22
GO:0000027	ribosomal large subunit assembly	GO:BP	3.27084E-14	22
GO:0000177	cytoplasmic exosome (RNase complex)	GO:CC	4.20105E-13	22
GO:0001650	fibrillar center	GO:CC	6.65056E-11	22
GO:0034475	U4 snRNA 3'-end processing	GO:BP	9.44435E-11	22
GO:0030020	extracellular matrix structural constituent conferring tensile strength	GO:MF	5.23144E-70	23
KEGG:04974	Protein digestion and absorption	KEGG	1.13188E-51	23
GO:0005615	extracellular space	GO:CC	3.12784E-36	23
GO:0030199	collagen fibril organization	GO:BP	1.29255E-33	23
GO:0004222	metalloendopeptidase activity	GO:MF	2.42713E-25	23
KEGG:04512	ECM-receptor interaction	KEGG	1.36643E-20	23
KEGG:04510	Focal adhesion	KEGG	4.46554E-14	23
GO:0008201	heparin binding	GO:MF	1.87255E-12	23
GO:0048407	platelet-derived growth factor binding	GO:MF	1.98065E-11	23
GO:0005587	collagen type IV trimer	GO:CC	5.05564E-11	23
GO:0005762	mitochondrial large ribosomal subunit	GO:CC	8.8535E-116	27
GO:0003735	structural constituent of ribosome	GO:MF	5.2581E-115	27
KEGG:03010	Ribosome	KEGG	5.50745E-75	27
GO:0005763	mitochondrial small ribosomal subunit	GO:CC	1.36717E-59	27
GO:0019843	rRNA binding	GO:MF	9.54753E-13	27
KEGG:05414	Dilated cardiomyopathy (DCM)	KEGG	1.73736E-34	28
KEGG:05410	Hypertrophic cardiomyopathy (HCM)	KEGG	5.90324E-33	28
GO:0051015	actin filament binding	GO:MF	1.86116E-25	28
GO:0008307	structural constituent of muscle	GO:MF	3.74042E-16	28
GO:0042383	sarcolemma	GO:CC	4.3611E-16	28
KEGG:04260	Cardiac muscle contraction	KEGG	8.71502E-16	28
KEGG:04261	Adrenergic signaling in cardiomyocytes	KEGG	1.02887E-14	28
GO:0030018	Z disc	GO:CC	1.57283E-14	28
GO:0005523	tropomyosin binding	GO:MF	8.1264E-14	28
GO:0005516	calmodulin binding	GO:MF	8.18777E-14	28
KEGG:05412	Arrhythmogenic right ventricular cardiomyopathy (ARVC)	KEGG	2.81566E-13	28
GO:0016012	sarcoglycan complex	GO:CC	2.14984E-12	28
GO:0002026	regulation of the force of heart contraction	GO:BP	4.31058E-12	28

GO:0045214	sarcomere organization	GO:BP	9.06402E-12	28
GO:0055010	ventricular cardiac muscle tissue morphogenesis	GO:BP	2.25329E-11	28
GO:0001725	stress fiber	GO:CC	2.8311E-11	28
GO:0055003	cardiac myofibril assembly	GO:BP	3.39231E-11	28
KEGG:00970	Aminoacyl-tRNA biosynthesis	KEGG	7.5109E-101	29
GO:0005524	ATP binding	GO:MF	3.07667E-38	29
GO:0000049	tRNA binding	GO:MF	1.60655E-29	29
GO:0017101	aminoacyl-tRNA synthetase multienzyme complex	GO:CC	6.54773E-27	29
GO:0002161	aminoacyl-tRNA editing activity	GO:MF	8.04888E-19	29
GO:0106074	aminoacyl-tRNA metabolism involved in translational fidelity	GO:BP	2.84778E-18	29
GO:0004826	phenylalanine-tRNA ligase activity	GO:MF	2.47463E-11	29
KEGG:00564	Glycerophospholipid metabolism	KEGG	8.2299E-97	30
KEGG:00600	Sphingolipid metabolism	KEGG	1.63766E-55	30
KEGG:01100	Metabolic pathways	KEGG	4.46199E-39	30
KEGG:00565	Ether lipid metabolism	KEGG	5.37736E-32	30
KEGG:00561	Glycerolipid metabolism	KEGG	9.86839E-32	30
KEGG:04975	Fat digestion and absorption	KEGG	1.72536E-22	30
KEGG:04071	Sphingolipid signaling pathway	KEGG	3.68926E-17	30
GO:0046512	sphingosine biosynthetic process	GO:BP	2.72443E-15	30
KEGG:00592	alpha-Linolenic acid metabolism	KEGG	6.51306E-14	30
GO:0004622	lysophospholipase activity	GO:MF	7.7005E-14	30
GO:0102567	phospholipase A2 activity (consuming 1,2-dipalmitoylphosphatidylcholine)	GO:MF	1.86789E-13	30
GO:0102568	phospholipase A2 activity consuming 1,2-dioleoylphosphatidylethanolamine)	GO:MF	1.86789E-13	30
KEGG:04972	Pancreatic secretion	KEGG	2.95558E-13	30
GO:0006657	CDP-choline pathway	GO:BP	1.17419E-12	30
GO:0016024	CDP-diacylglycerol biosynthetic process	GO:BP	3.7234E-12	30
GO:0050482	arachidonic acid secretion	GO:BP	2.62062E-11	30
GO:0003841	1-acylglycerol-3-phosphate O-acyltransferase activity	GO:MF	4.36858E-11	30
GO:0009952	anterior/posterior pattern specification	GO:BP	1.00962E-19	33
GO:0000980	RNA polymerase II distal enhancer sequence-specific DNA binding	GO:MF	3.97319E-18	33
GO:0048704	embryonic skeletal system morphogenesis	GO:BP	3.9022E-17	33
KEGG:03018	RNA degradation	KEGG	1.92697E-23	66
GO:0017148	negative regulation of translation	GO:BP	2.16483E-19	66
GO:0004535	poly(A)-specific ribonuclease activity	GO:MF	1.71388E-13	66
GO:0030015	CCR4-NOT core complex	GO:CC	3.18213E-13	66
GO:0031047	gene silencing by RNA	GO:BP	9.9334E-13	66
GO:0090503	RNA phosphodiester bond hydrolysis, exonucleolytic	GO:BP	2.48133E-12	66
GO:0000932	P-body	GO:CC	4.97345E-11	66

Supplementary Table S8. In- and out-degree for each community in the HSA network

community	*in-degree*	*out-degree*	*community*	*in-degree*	*out-degree*
0	29.93	24.40	13	39.43	33.94
1	24.82	19.78	14	33.29	18.48
2	24.09	13.56	15	23.84	30.17
3	15.06	51.87	16	34.40	25.24
4	23.61	57.90	18	23.94	14.85
5	30.05	23.79	19	23.88	32.37
6	34.06	35.12	20	34.89	8.25
7	39.86	32.54	22	15.81	6.03
8	39.89	34.23	23	19.94	31.11
9	36.12	13.91	28	24.34	63.13
10	39.80	17.21	30	25.79	10.52
11	31.00	55.16	33	4.35	4.20
12	25.94	39.87	35	34.37	34.87

Supplementary Table S9. In- and out-degree for each community in the MMU network

community	*in-degree*	*out-degree*	*community*	*in-degree*	*out-degree*
0	19.35	31.81	15	19.30	14.18
1	20.05	73.43	16	26.80	9.26
3	26.44	32.89	17	26.09	11.18
4	28.42	5.44	19	16.47	8.35
5	36.00	35.95	20	19.46	16.83
6	30.18	33.94	21	18.08	0.00
7	22.11	27.98	22	15.99	20.18
8	33.55	14.68	23	20.31	23.46
9	11.36	13.93	27	8.54	32.01
10	20.70	21.58	28	21.66	55.51
11	29.72	11.38	29	15.69	0.00
12	36.73	23.10	30	27.91	5.19
13	35.63	24.51	33	4.89	43.32
14	26.94	8.49	66	44.01	63.83
